# Marked reduction in fertility among African women with urogenital infections: A prospective cohort study

**DOI:** 10.1371/journal.pone.0210421

**Published:** 2019-01-10

**Authors:** K. Perslev, O. A. Msemo, D. T. R. Minja, S. L. Møller, T. G. Theander, J. P. A. Lusingu, I. C. Bygbjerg, B. B. Nielsen, C. Schmiegelow

**Affiliations:** 1 Centre for Medical Parasitology, Department of Immunology and Microbiology, University of Copenhagen, Copenhagen, Denmark; 2 National Institute for Medical Research, Korogwe, Tanga, Tanzania; 3 Division of Global Health, Department of Public Health, Copenhagen University, Copenhagen, Denmark; 4 Department of Obstetrics and Gynaecology, Aarhus University Hospital, Aarhus, Denmark; University of Insubria, ITALY

## Abstract

**Background:**

There is paucity of data on risk factors for reduced fertility in low-income countries.

**Objective:**

To investigate factors associated with fertility among women in rural north eastern Tanzania.

**Subjects and methods:**

A cohort of 1248 non-pregnant women was followed with urine pregnancy testing every third month or more regularly if they reported a missed menstrual period. Pregnancy was confirmed with trans-abdominal ultrasound. Information regarding general health, socioeconomic status and obstetric-gynaecological history was collected. Factors associated with conceiving within 180 days were identified using multivariate logistic regression analyses.

**Results:**

Among the 1248 women, 736 were followed for 180 days and 209 of these had an ultrasound confirmed pregnancy. During the follow-up period, 169/736 women were diagnosed with urogenital infections, including suspected sexually transmitted or reproductive tract infections, urinary tract infection, and vaginal candidiasis. Urogenital infections were significantly associated with reduced odds of conceiving within 180 days (adjusted OR (AOR) 0.21, 95% CI 0.11–0.36). Being above 30 years of age was also negatively associated with odds of conceiving (AOR 0.45, 95% CI 0.26–0.77). In contrast, women who recently stopped using hormonal contraceptives (AOR 2.86, 95% CI 1.45–5.70) and women with low socioeconomic status (AOR 1.56, 95% CI 1.04–2.33) were significantly more likely to become pregnant within 180 days.

**Conclusion:**

Urogenital infection seems to be a major health factor associated with reduced chances of conceiving. Considering the availability of effective treatment options for these diseases, public health authorities should increase awareness of diagnostic tools in settings with limited resources in order to improve fertility.

## Introduction

The World Health Organization (WHO) defines infertility as “*A disease of the reproductive system defined by the failure to achieve a clinical pregnancy after 12 months or more of regular unprotected sexual intercourse*” [1]. During the last three decades there has been an increasing attention on infertility, and fertility is now mentioned in the reproductive rights defined by WHO [2]. There is an increasing demand for assisted reproductive technology and research defining the causes of infertility in high income countries (HIC) [3–5]. However, the causes of infertility in low- and middle-income countries (LMIC) remain poorly defined [6]. In 2002, it was estimated that approximately 186 million women in the reproductive age worldwide were infertile defined as the number of ever married women with no birth the last five years [7]. Due to methodological challenges, an exact estimate of the number of women and couples suffering from infertility is difficult to obtain, but most studies estimate the global infertility rate to be around 10% [8–12]. In Sub-Saharan Africa infertility rates are higher with estimates from 15% to 40% [9, 10, 13, 14].

The high prevalence of infertility in LMIC is a major healt concern due to limited availability of treatment and the social, cultural, and economic consequences of infertility in these regions [9, 14]. Women living in infertile relationships are at risk of experiencing domestic violence, depression, social exclusion, stigmatization, polygamy [15, 16] and it can also dramatically influence the economic situation of families [12, 16].Furthermore infertility can prevent young girls from gaining respect and status as a women in the eyes of the society, which can catch them in a limbo between two stages of life [17].

Identifying factors associated with infertility is an essential step in establishing adequate treatment and preventive strategies. Maternal age [18], smoking [19], alcohol abuse [20], genital infections [21, 22] and body mass index (BMI) [23] have been shown to affect fertility in high income countries (HIC). However, there is a paucity of data on causes for infertility in LMIC, where there is a considerably higher frequency of secondary infertility (inability to become pregnant or to carry a pregnancy to a live birth following a previous pregnancy) [24]. A large older study concluded that infertility in Africa could be attributed to infections in 85% of the cases, and that the majority of these infections were either sexually transmitted infections (STI) or infections acquired following delivery or illegal abortion [25].

The aim of the present study was to define factors associated with the chances of conceiving among women in rural Tanzania. Identification of such factors may be an important step towards implementing health- and socioeconomical actions to reduce infertility.

## Methods

The study was part of the preconceptional and pregnancy cohort study “**Fo**etal exposure and **E**pidemiological **T**ransition: the role of **A**naemia in early **L**ife for **N**on-**C**ommunicable-**D**iseases later in life” (FOETALforNCD) conducted in 48 rural and semi-urban villages of Korogwe and Handeni Districts, Tanga Region, North-east Tanzania from July 2014 until December 2016. The FOETALforNCD study consisted of two sub-studies: a preconceptional and pregnancy cohort study following women before pregnancy as well as throughout pregnancy until delivery if they conceived, and a pregnancy cohort study following women who were < = 14 weeks pregnant at enrolment and followed until delivery. We here present data on the preconceptional part of the study.

### Inclusion and exclusions criteria for FOETALforNCD

Non-pregnant women were enrolled from July 2014 until December 2015. The preconceptional follow-up period was from July 2014 to March 2016 and women found to be pregnant before the 31^st^ of March 2016 were enrolled in the pregnancy part of the study. Inclusion criteria in the preconceptional part were: age 18–40 years, not using modern contraceptive methods except for condoms, not having a child of less than nine months of age, not having tried unsuccessfully to conceive for more than two consecutive years (perceived as sub-fertile), a negative urine pregnancy test (UPT) (HCG, sensitivity of 25 miU/ml HCG, Vista Care Company, Shandong, China), living in an accessible area, and willingness to attend all project activities including giving birth at Korogwe District Hospital if conceiving during the study period. There were no additional exclusion criteria.

### Enrolment

Upon enrolment, a comprehensive questionnaire (S5, S6 and S7 Tables) was filled out including detailed information on demographic data, socioeconomic status, tobacco, alcohol, and caffeine consumption, usage of family planning, gynaecological diseases, menstrual cycle pattern, obstetric history, and medical history including known chronic anaemia, diabetes mellitus, hypertension, kidney disease, cancer, or HIV, and recent anaemia or malaria. A medical examination was conducted including blood pressure (r-champion N, Rudolf Riester, Jungingen, Germany, automated) and anthropometric measurements. Anthropometric measurements included weight measured while barefooted and wearing light clothes (precision 0.1kg, digital weighing scales, Seca GmbH & Co. KG, Hamburg, Germany), height (stadiometer, precision 1 cm, Seca GmbH & Co. KG., Hamburg, Germany), and mid-upper arm circumference (MUAC), waist circumference measured just above the iliac crest in the horizontal plane, and hip circumference measured at the point yielding the maximum circumference over the buttocks, all using a standard measuring tape to the nearest 0.1 cm [26]. Body mass index (BMI) in kg/m^2^ was categorized as underweight (<18.5), normal (18.5–24.9), overweight (25.0–29.9) and obese (≥ 30.0) [27]. Normal cycle length was defined as 25–34 days [28]. Hypertension was defined as systolic blood pressure ≥140 mmHg and/or diastolic blood pressure ≥90 mmHg observed at two different occasions, or as known hypertension before enrolment and on antihypertensive medication. To enable immediate treatment, point-of-care diagnostics were used at enrolment to capture anaemia (HemoCue HB 301 analyzer, HemoCue AB, Angelholm, Sweden), diabetes (HemoCue Glucose 201 analyzer, HemoCue AB, Angelholm, Sweden), malaria (rapid diagnostic test for malaria, ParaHIT, Span Diagnostics, Gujarat India), HIV if the women consented (Rapid diagnostic test for HIV, Alere Determine HIV-1/2 test kit, Alere LTD, Stockport, UK, and confirmed using a Unigold test kit, Trinity Biotech plc, Wicklow, Ireland), and signs of urinary infections (Urine dipstick, Combiscreen 7, Alere, Massachusets, USA). Diagnostics (point-of-care or in centralized laboratory) for other sexually transmitted and reproductive tract infections (STI/RTI) were not available. However, women with relevant gynaecological symptoms were offered gynaecological ultrasound and a gynaecological examination by the study physicians (CS and OA). Furthermore, 15 mL of venous blood was collected in ethylenediamine tetra acetic acid (EDTA) coated and plain serum tubes and transported at 2–8°C to the National Institute for Medical Research (NIMR) Korogwe Research Laboratory and processed within two hours of sample collection. In the NIMR Korogwe Research Laboratory, full haematological blood picture, HbA1c, and C-reactive protein (CRP) were measured using Sysmex KX-21N haematological analyser (Sysmex Corporation Kobe, Japan) and Afinion AS 100 analyser (Axis Shield PoC, AS, Oslo Norway), respectively.

Diabetes mellitus were diagnosed as random blood sugar > 11.1 mmol/L at two different consultations, or fasting blood glucose >7 mmol/L, or HbA1c >6.5% (Afinion AS 100 analyser, Axis Shield PoC, AS, Oslo Norway). Anaemia (hgb concentration <12 g/dL) was treated with combination tablets of FeFo (200 mg ferrous sulfate (~ 43 mg elemental iron) and 400 μg folate per day (Ferrolic–LF, Laboratory and Allied LTD, Mombasa, Kenya) or Hemovi multivitamin syrup containing 200 mg Ferrous sulfate, 0.5 mg B6, 50 μg B_12_, 1.5 mg Folic acid and 2.33 mg zinc sulphate per 5mL (Shelys Pharmaceuticals, Dar es Salaam, Tanzania). Malaria was treated with oral Artemether-lumefantrine (Lumartem 20mg/120mg (CIPLA LTD, Patalganga, India). HIV seropositive women were referred to the governmental Care and Treatment Clinics and managed according to national guidelines. Urinary tract infections (UTI, clinical symptoms and leucocytes and/or nitrite on urine dipstick) were treated with amoxicillin 500 mg three times a day for three days (North China Pharmaceuticals Group Formulation Company Ltd. Shijazhuang, China) and suspected vaginal candidiasis (vaginal itching and whitish curd-like vaginal discharge) with clotrimazole vaginal suppository 100 mg daily for 5 days (Laboratory and Allied LTD, Mombasa, Kenya). Other suspected genital infections were managed with syndromic treatment with ciprofloxacin 500 mg once, doxycycline 100 mg twice a day for 7 days, and 2g metronidazole once (all from Astra lifecare, Ahmedabad, India).

### Follow up

After enrolment, the women were followed every third month with UPT. Women were also encouraged to report before their scheduled visit if they suspected to be pregnant. From March 2015, to avoid overlooking any pregnancies, ultrasound was performed if a menstrual period was missed disregarding a negative UPT. If UPT positive and/or if suspecting to be pregnant, transabdominal ultrasound were performed (5–2 MHz abdominal probe, Sonosite TITAN and Sonosite Turbo, US High resolution, Sonosite, Bothell, WA, USA). Gestational age was estimated using crown rump length in the 1^st^ trimester [29] or head circumference in the 2^nd^ trimester [30] if pregnancy was confirmed. Haemoglobin (hgb) level concentration, urine dipstick, and anthropometric measurements were routinely repeated, and all other tests were repeated based on presented symptoms and at the clinician´s discretion. Any diseases diagnosed at follow up were treated as described under Enrolment.

### Definition of women eligible for inclusion in analyses on fertility

Only women who were confirmed to be truly preconceptional at the time of screening were included. This entailed either 1) having negative UPT at all visits and that a pregnancy was never confirmed with ultrasound, or 2) having a positive UPT at a follow-up visit combined with ultrasound confirming she conceived after enrolment. Women who started using modern family planning, were considered to enter menopause, or withdrew consent during the preconceptional follow-up period were excluded. Finally, the design of the FOETALforNCD study resulted in enrolment at different time points, this gave rise to different follow-up periods, since preconceptional follow-up ended by March 2016 for all enrolled women disregarding the time-point of enrolment. To equalize the probability of becoming pregnant for all women included in the present analyses, only women followed for at least 180 days were considered. Fertility was analysed as a binary outcome defined as having either a clinical pregnancy confirmed with ultrasound or not conceiving within 180 days of follow-up. Women who conceived after the 180 days were kept in the “not conceived” group in all analysis. Biochemical pregnancies (positive UPT, never confirmed with ultrasound) were excluded.

### Ethical considerations

Ethical clearance was granted by the Medical Research Coordinating Committee of the National Institute for Medical Research (reference number NIMR/HQ/R.8a/Vol. IX/1717). Village leaders and community members were explained about the study followed by house-to-house sensitization meetings where couples were explained about the study before the women were invited to the nearby health post for screening and enrolment. Written informed consent (or thumbprints of illiterate women) was obtained from all women prior to enrolment. All procedures were conducted in accordance with the Declaration of Helsinki and Good Clinical and Laboratory Practice. Permission to publish has been given by the National Institute For Medical Research n Tanzania.

### Statistics

Statistical analyses were performed using “R” (R- studio, version 1.1.38). The approach aimed at revealing important explanatory factors to the binary fertility response variable. A variant set of explanatory variables were evaluated including categorical and continuous factors. The continuous variables were analysed as continuous as well as categorized. The categorization was done in clinically relevant groups in order to facilitate detection of possible non-linear relations and to facilitate comparison to clinical literature. An initial univariate analysis was conducted in search of factors with correlation to pregnancy. Contingency tables were created between categorical factors and the response variable and tested for dependence using Fisher’s Exact Test. Students t-test was used for continuous variables being normally distribuated (hgb, hgb change during study, menstrual cycle length and BMI) and Mann-Whitney U test for continuous variabels not normally distributed (age and parity). Variables having a p-value<0.2 in the univariate analyses were included in the initial multiple logistic regression model. Furthermore, variables known to be biologically plausible factors associated with fertility (BMI, current use of contraception, gynaecological diseases, previous abdominal operation) were also included in the multivariate model disregarding the result in the univariate analyses. Based on this a logistic regression model was initially fitted to a manually curated set of explanatory variables. We then performed further model selection through backwards step-wise elimination of variables by comparing the relative quality of candidate models using the Akaike Information Criterion (AIC). AIC estimates the quality of a model by evaluation the goodness of fit against model complexity. Between two equally performing models, AIC penalizes the model of higher complexity (more explanatory variables) relative to the simpler model [31]. We chose the model minimizing AIC using the step algorithm implemented in R. In order to apply such model selection, the full model was fit to only complete samples across the included variables. The final model was fit to the data of 669 women. A p-value <0.05 was considered statistically significant. In order to test the robustness of the model on 180 days follow-up, models for 90 and 360 days follow up was also fitted. (S1 Table).

## Results

**Fig 1:**
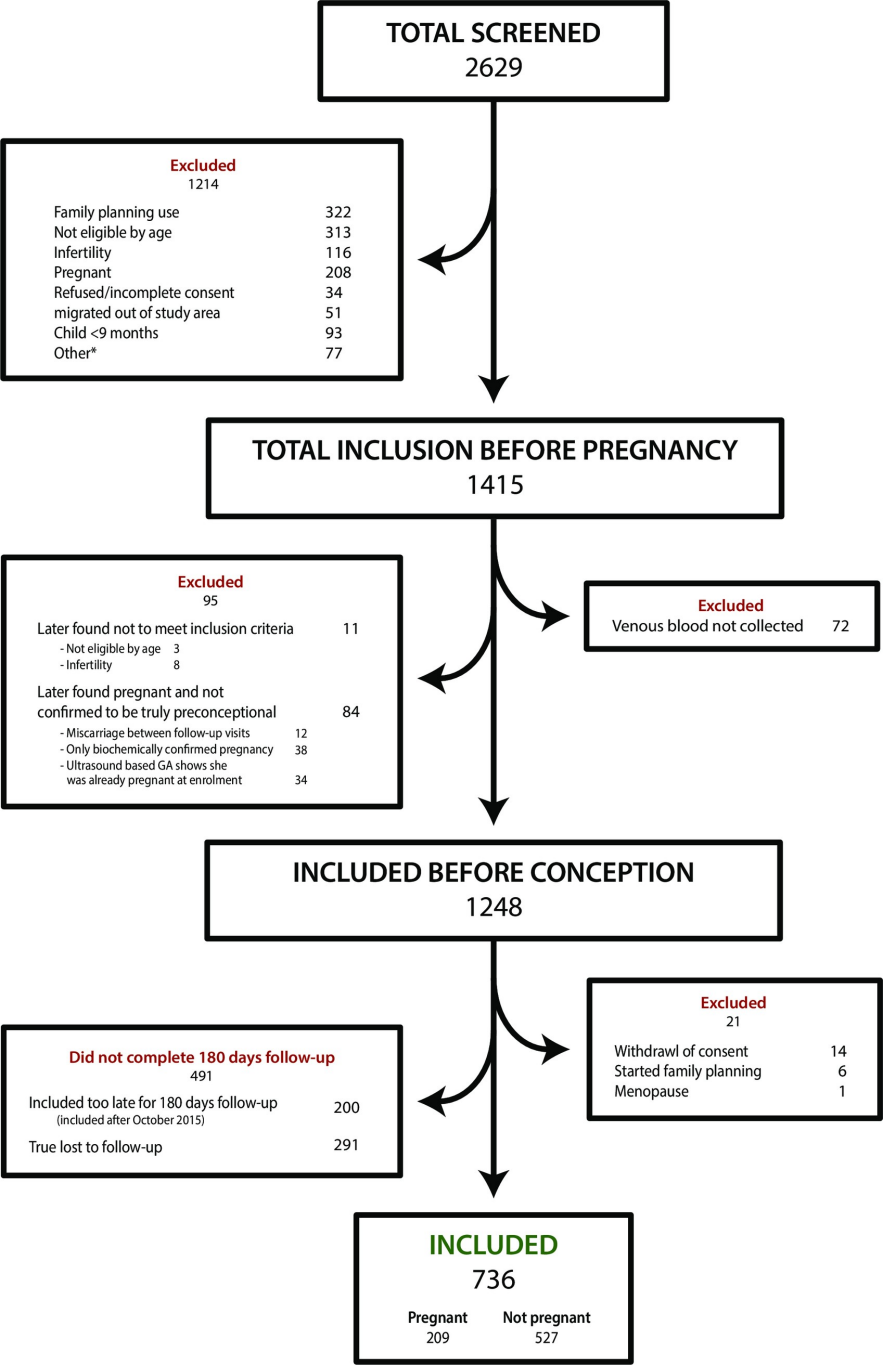
Flowchart of inclusion in study.

A total of 2629 women were screened, 1415 women were enrolled and 1227 women were eligible for analyses (Fig 1). Among the 1227 women, 736 women were followed for at least 180 days, and among these 209 (28%) women conceived. The Grouped characteristics of the 736 women are shown in Table 1.

**Table 1:** Characteristics of 736 women.

Characteristic	Interval	N-total	n (%)
Age	18–25	731	218 (29.8)
	25–30		153 (20.9)
	30–35		154 (21.1)
	35–40		206 (28.2)
Partner status	Living with partner	731	546 (74.7)
	Partner–not living together		87 (11.9)
	No partner		98 (13.4)
Education	Non	736	72 (9.8)
	Primary school partially		562 (76.4)
	Secondary school and higher		102 (13.9)
Job	Professional	736	12 (1.6)
	Business		103 (14.0)
	Farmer		470 (63.9)
	Service		27 (3.7)
	House wife		108(14.7)
	Student		16(2.2)
House roof	Corrugated iron	736	528 (71.7)
	Not corrugated iron		208 (28.3)
BMI	<18.5	725	54 (7.4)
	18.6–24.9		438 (60.4)
	25–29.9		148 (20.4)
	>30		85 (11.7)
Parity	Nullipara (0)	734	103 (14,0)
	Primipara (1)		132 (18.0)
	Multipara (2–4)		373 (50,8)
	Grand multipara (5+)		124 (16.9)
Previous extra-uterine pregnancy		736	5 (0.7)
Length of menstrual cycle	Short	693	25 (3.6)
	Normal		584 (84.3)
	Long		84 (12.1)
Gynecological diseases*		736	21 (2.9)
Abdominal operations**		735	65 (8.8)
HIV seropositive		600	34 (5.7)
Malaria during study		736	85 (11.5)
Urogenital infections during study***		736	169 (23.0)
Hgb at enrolment (g/dL)	<9	736	19 (2.6)
	9–11.9		247 (33.6)
Contraception before study		736	586 (79.6)
Hormonal contraception before study		736	495 (67.3)
Traditional contraception before study		736	278 (37.8)
Condom usage before study		736	178 (24.2)
Termination of hormonal contraception	<3 months	736	58 (7.9)
	3–6 months		68 (9.2)
	6–12 months		77 (10.5)
	>12 months		202 (27.4)
	Never used		241 (32.7)
	Used, term. date unknown		90 (12.2)
Termination of traditional contraception	<3 months	736	7 (1.0)
	3–6 months		11 (1.5)
	6–12 months		15 (2.0)
	>12 months		41 (5.6)
	Never used		458 (62.2)
	Used, term. date unknown		34 (4.6)
	Still using		170 (23.1)
Current use of contraception ****		736	212 (28.8)

N-total is the total number of women with available answers in each category. n is the number of cases.

* Gynecological diseases: fibroma, ovarian cysts, or specifics unknown to the patient.

**Abdominal operations: for extra uterine pregnancies, caesarian sections, or appendicitis.

*** Urogenital infections include suspected sexual transmitted/reproductive tract infections, urinary tract infections, and suspected vaginal candidiasis

**** Current us of contraception: Condom or traditional contraception

There were more women represented in the groups of 18–25 years and of 35–40 years than in the middle age groups. The majority were living with their partner. Educational level was low with only 13.9% having completed secondary school or beyond (Table 1). Lifestyle factors can affect fertility, but the effect of caffeine intake, alcohol and smoking were not investigated due to low daily exposure level in the study population: Only five enjoyed more than one small Coca-Cola, 14 had one or two cups of coffee, and 15 had three or more cups of tea. 31 women drank alcohol, of which only 5 consumed more than 5 per week. Only two smokers were identified in the study.

According to BMI, the majority were normal weight, and the remaining was primarily overweight or obese. In total, 84.3% reported a normal menstrual cycle and only few women (2.9%) had a gynaecological disease diagnosed at enrolment or during follow-up, or had a previous abdominal operation (8.8%). A large proportion of the women had anaemia (36.2%). Urogenital infections were also prevalent in the cohort: at enrolment or during a follow up visit, 29 had a suspected STI/RTI, 98 had suspected vaginal candidiasis, and 86 had an UTI. HIV and malaria were also present (34 women and 85 respectily). Use of contraceptives before enrolment was common, and 28.8% were still using either condom or traditional contraception (periodic abstinence or withdrawal) at enrolment (Table 1).

### Factors associated with fertility–univariate analysis

Increasing age, parity <1 or > = 5, infection with HIV, and urogenital infections were significantly more prevalent among women who did not conceive. Recent termination of hormonal contraceptives and living with a partner were more common among women who conceived. There was a trend towards poor socioeconomic status measures, such as educational level below secondary school, not having a corrugated iron roof, and working in business, as a farmer, or in a service job, were more common among women conceiving.

A number of variables not included in Tables 1 and 2 were also tested and not found significantly associated with conceiving (S2 Table). These included other anthropometric measurements, other socio-economic factors, male factors (age, education, religion), diabetes mellitus, and hypertension. There was no significant association between conceiving and hgb at enrolment or change in hgb during the follow-up period (hgb at enrolment: difference between the two groups: -0.06 g/dl, CI 95% -0.31 to 0.18, p = 0.62; change in hgb during follow-up period: difference between the two groups: 0.14 g/dl, CI 95% -0.51 to 0.22, p = 0.43).

**Table 2:** Factors affecting the chance of conceiving within 180 days.

Characteristic	Interval	N total	N (%) Non preg.	N total	N (%) Pregnant	p-value
Age	18–25	523	143 (27.3)	208	75 (36.1)	0.03*
	25–30		105 (20.1)		48 (23.1) (23.735/208 )	
	30–35		115 (22.0)		39 (18.8)	
	35–40		160 (30.6)		46 (22.1)	
Partner status	Living with partner	523	369 (70.1)	208	177 (85.1)	<0.001***
	Not living with partner		69 (13.2)		18 (8.7)	
	No partner		85 (16.3)		13 (6.3)	
Education	None	527	57 (10.8)	209	15 (7.2)	0.06
	Primary school partial/complete		390 (74.0)		172 (82.3)	
	Secondary school and higher		80 (15.2)		22 (10.5)	
Job	Professional	527	10 (1.9)	209	2 (1.0)	0.06
	Business		72 (13.7)		31 (14.8)	
	Farmer		332 (63.0)		138 (66.0)	
	Service		17 (3.2)		10 (4.8)	
	Housewife		80 (15.2)		28 (13.4)	
	Student		16 (3.0)		0 (0)	
House roof	Corrugated iron	527	388 (73.6)	209	140 (67.0)	0.08
	Not corrugated iron		139 (26.4)		69 (33.0)	
BMI	<18.5	517	41 (7.9)	208	13 (6.3)	0.64
	18.6–24.9		310 (60.0)		128 (61.5)	
	25–29.9		109 (21.1)		39 (18.8)	
	>30		57 (11.0)		28 (13.5)	
Parity	Nullipara (0)	523	82 (15.7)	209	21 (10.0)	0.006**
	Primipara (1)		88 (16.8)		44 (21.1)	
	Multipara (2–4)		250 (47.8)		123 (58.9)	
	Grand multipara (5+)		103 (19.7)		21 (10.0)	
Previous extra-uterine pregnancy		527	4 (0.8)	209	1 (0.5)	1
Length of menstrual cycle	Short	501	22 (4.4)	192	3 (1.6)	0.07
	Normal		413 (82.4)		171 (89.1)	
	Long		66 (13.2)		18 (9.4)	
Gynaecological diseases		522	17 (3.3)	209	4 (1.9)	0.46
Previous abdominal operations		526	51 (9.7)	209	14 (6.7)	0.25
HIV seropositive		447	33 (7.4)	153	1 (0.7)	<0.001***
Malaria during study		527	59 (11.2)	209	26 (12.4)	0.61
Urogenital infections during the study		527	150 (28.5)	209	19 (9.1)	<0.001***
Hgb at enrolment (g/dL)	<9	527	14 (2.7)	209	5 (2.4)	1
	9–11.9		177 (33.6)		70 (33.5)	
Contraception before study		527	412 (78.2)	209	174 (83.3)	0.13
Hormonal contraception before study		527	346 (65.7)	209	149 (71.3)	0.16
Traditional contraception before study		527	205 (38.9)	209	73 (34.9)	0.35
Condom usage before study		527	136 (25.8)	209	42 (20.1)	0.11
Termination of hormonal contraception	<3 months	527	27 (5.1)	209	31 (14.8)	<0.001***
	3–6 months		42 (8.0)		26 (12.4)	
	6–12 months		55 (10.4)		22 (10.5)	
	>12 months		149 (28.3)		53 (25.4)	
	Never used		181 (34.3)		60 (24.9)	
	Used. Term. date unknown		73 (13.9)		17 (28.7)	
Termination of traditional contraception	<3 months	527	3 (0.6)	209	4 (1.9)	0.03*
	3–6 months		7 (1.3)		4 (1.9)	
	6–12 months		12 (2.3)		3 (1.4)	
	>12 months		37 (7.0)		4 (1.9)	
	Never used		322 (61.1)		136 (65.1)	
	Used, Term. date unknown		21 (4.0)		13 (6.2)	
	Still using		125 (23.7)		45 (21.5)	
Current use of contraception		527	161 (30.6)	209	51 (24.4)	0.10

N is the total number of women with answers available in each categories, N (%) not-pregnant is the number and percent of women in the subcategory that did not become pregnant in 180 days, N (%) pregnant is the number and percent of women in the subcategories that became pregnant within 180 days. p-value for each group found with Fisher´s Exact test.

* p-value < 0.05

** p-value < 0.01

*** < 0.001,.

### Factors associated with fertility–multivariate analysis

To assess the ability of different conditions or factors to predict whether a woman became pregnant during follow-up data was fitted to a multivariate logistic regression model (Table 3). Having a urogenital infection significantly decreased the odds of conceiving (adjusted Odds Ratio (AOR) 0.21, 95% CI 0.11–0.36, p-value <0.001)^.^

**Table 3:** Multivariate model for factors significantly affecting change of conceiving.

Characteristic	Interval	OR	95%CI	p-value
Age	18–25	Ref re		
	25–30	0.74	0.44–1.2	0.23
	30–35	0.45	0.26–0.77	0.004**
	35–40	0.43	0.26–0.71	0.001**
Partner status	No partner	Ref		
	Living with partner	3.55	1.84–7.39	<0.001***
	Partner—not living together	1.91	0.83–4.53	0.13
Urogenital infection	No	Ref		
	Yes	0.21	0.11–0.36	<0.001***
House roof	Corrugated iron	Ref		
	Not -corrugated iron	1.56	1.04–2.33	0.04*
Terminated hormonal contraception	Never used	Ref		
	<3 months	2.86	1.45–5.70	0.002**
	3–6 months	1.51	0.78–2.89	0.21
	6–12 months	1.27	0.64–2.46	0.49
	> 12 months	1.11	0.67–1.84	0.70
	Used, term date unknown	0.91	0.45–1.76	0.78

Logistic regression model predicting pregnancy probability from explanatory variables selected from a larger set via backwards step-by step elimination. OR with 95%-CI shown for the different subgroups and Wald test p-values comparing the subgroup with the reference subgroup.

* p-value < 0.05

** p-value < 0.01

*** p-value < 0.001. A similar analysis without grouping of continuous variables can be seen in the supplementary material (S3 Table).

Age was associated with a decreased fertility for the age group 30–35 (AOR 0.45, 95% CI 0.26–0.77, p-value = 0.004) and 35–40 (AOR 0.43, 95% CI 0.26–0.71, p-value = 0.001) compared to the reference group of age 18–24. Living in house without a corrugated iron roof (indicating a low socioeconomic status in this population) increased the chance of conceiving (AOR 1.56, 95% CI 1.04–2.33, p-value 0.04). Women who had stopped using hormonal contraceptives within the last 3 months had significantly higher likehood of becoming pregnant than women with no previous use of hormonal contraception (AOR 2.86, 95% CI 1.45–5.70, p-value = 0.0025). Women living with a partner were significantly more likely to become pregnant than women who reported no current partner (AOR 3.55, 95% CI 1.84–7.39, p-value = 0.0003).

Urogenital infections were defined as suspected STI/RTI, suspected vaginal candidiasis, or UTI. When analysed as separate entities UTI and candida were significantly associated with reduced change of conceiving (OR 0.33, CI95% 0.15–0,68, p-value<0.001 and OR: 0.21, CI95% 0.086–0.45, p-value<0.001, respectively). Eventhough STI/RTI did not show a significant association (OR: 0.68, CI95% 0.19 1.7, p value = 0.51; possibly due to the relative few suspected cases) this variable was retained in the final model since STI/RTI is suspected to have an effect on fertility.

To test the robustness of the conclusions, we performed two variations of the analyses: 1) We considered a follow up period of 90 days and 360 days, respectively (S1 Table). In both models urogenital infections had a significant effect on the likelihood of conceiving. 2) We reanalysed the data as continuous variables for hgb, age, menstruation cycle length, parity, body mass index (S3 Table). This alternative model was constructed with the exact same selection approach described in the method section and shown in Table 3 and resulted in the same conclusions.

## Discussion

### The effect of urogenital infections

Despite treatment being instigated for any suspected infection, the presence of urogenital infections was associated with a marked decrease in chance of conceiving during a follow-up period of 180 days. Sexually transmitted infections (STI) are a known cause of secondary infertility, as well as a known cause of morbidity in LMIC [24, 32, 33]. In Tanzania, previous studies have reported a 43–64% prevalence of STIs [34–36]. However, in this study area, as well as in the rest of rural Tanzania, facilities for bacteria cultivation or PCR diagnosis are not available and the diagnostic possibilities for STIs, RTIs, and vaginal candidiasis are limited to anamnestic questions and urine dipsticks. To minimize the risk of misclassification, we therefore grouped UTI, suspected STI/RTI, and candidiasis as urogenital infections. In support of this approach, an explorative analysis of the individually subgroups (STI/RTI, UTI, or vaginal candidiasis) suggested the three conditions were all negatively correlated with fertility, although this was only stastistically significant for UTI and vaginal candidiasis. To our knowledge previous studies have not shown an association between a single UTI or a candida infection and reduced fertility. Our findings might be explained by clinical misclassification, and it is possible that some of the diagnosed UTI and/or vaginal candidiasis were actually STIs. UTI might have been over-diagnosed since contamination of urine with vaginal secretion would lead to positive urine strips for leucocytes, protein or blood, and mid-stream urine sampling or disinfection of external genitals was not undertaken routinely. This would have been preferable in order to distinguish between the diagnoses [37].

The relative contribution of STI/RTI, vaginal candidiasis, and UTI to reduced conception rates in our setting obviously requires further research, but a change towards more intensive treatment of both suspected STI´s and urogenital infections in areas with limited diagnostic possibilities could be warranted. These conditions are easily treated with antibiotics and antifungals. In addition, better diagnostic tools for chlamydia, gonococcus, and trichomoniasis are urgently needed, since there are no point of care tests at the moment [38] and syndromic treatment has previously been shown to have low sensitivity [39].

### The effect of age, partner status, socioeconomic status, and contraception

As found by Crawford et al [18], age was also statistically significantly associated with reduced fertility in our study. Living with a partner and having a lower socioeconomic status (ranked by type of roof) also exhibited the expected association with increased chances of becoming pregnant. Although use of traditional family planning was not significantly associated with reduced fertility, low socioeconomic status might reflect a poorer pregnancy control based on traditional contraception, as suggested by Ayele et al [40].

Among the women who conceived, 24% used condoms and/or traditional family planning. Others have also reported high rates of conceiving despite usage of condoms and traditional family planning [41]. The high reported usage among women who conceived might indicate that reported use is not equal to actual or constant use. Irregular use of condoms is of particular concern considering the high prevalence of STI observed.

Compared to women with no previous use of hormonal contraceptives, women who recently terminated usage were more likely to conceive, whereas women who terminated hormonal contraceptives >90 days ago were not. Studies on hormonal contraception and fertility have not consistently shown any negative effect on fertility [42] and no study has—to the best of our knowledge—showed increased fertility due to previous use of hormones. The explanation for the observed increase in fertility in the group could be that the women who recently stopped using hormonal contraceptives were actively trying to become pregnant with a higher frequency and timing of sexual intercourse. In contrast, women who had terminated the usage of hormone contraception for a long time and still had not become pregnant before enrolment into our study actually represented a less fertile group.

### The effect of other health factors

Other health factors investigated in this study including malaria, diabetes, anaemia, and hypertension did not show any statistically significant effect on the chances of conceiving in the multivariate model. In contrast to findings in other studies, gynaecological diseases such as fibromas did not show any association with reduced fertility either [43]. This may be due to the low number of women diagnosed with fibroma in our study. In the univariate analysis HIV positive women had a significantly lower chance of conceiving. The total number of HIV positive women in the study was 34 and only one of them became pregnant. The data was therefore too limited to be included in the multivariate analysis. The results, however, show a strong tendency of lower fertility in HIV infected women, which is in accordance with previous reports [44]. Anaemia has in smaller clinical studies, and in animal studies been suspected to reduce fertility [45, 46]. It was not possible to demonstrate this association in the present study, also not when adjusting for changes in Hgb concentration during follow-up.

### Strengths and weaknesses

The follow-up period was not identical for all women. The first women enrolled could potentially be followed for more than 1½ year (July 2014 –March 2016), whereas women enrolled in December 2015 could only be followed for three months. In order to address this, we only included women who were followed for at least 180 days and only included pregnancies that occurred within this period, which meant a considerably reduced sample size. However, the robustness of our approach was confirmed by generating both a 90 days and a 360 days model yielding similar results as obtained with 180 day follow-up.

The present study was part of the FOETALforNCD project, aiming to elucidate the role of maternal anaemia in the development of epigenetic changes in the new-born with possible consequences for the later development of life-style diseases [47, 48]. FOETALforNCD was not designed as a fertility study and inclusion criteria were based on a likelihood of becoming pregnant. Women using traditional contraceptive methods and condoms have a considerable chance of becoming pregnant within a year [41] and were therefore enrolled. Furthermore, in order to avoid disclosing whether the woman had a partner, partner status was not used as an inclusion criterion, and only limited information on the partner was collected after enrolment. The partner could therefore not be evaluated. Information on sexual activity and the desire to conceive was also not available. This could have introduced bias and the results presented here can therefore not be immediately applied to other populations. However, our rigorous classification of preconceptional status using both UPT and gestational age estimated with ultrasound strengthens our study and gives an indication of important factors associated with chances of conceiving in the area. Furthermore, we provide an indication of how many women can be expected to become pregnant within the defined time period, which can aid in planning, designing and executing future preconception and pregnancy studies–and hopefully also aid women (and partners) wanting to plan their families.

## Conclusion

Urogenital infection was the major health factor associated with a reduced chance of conceiving. Considering the availability of effective treatment options for these diseases, public health authorities in Tanzania should increase awareness of diagnostic tools in poor settings in order to improve chances of getting pregnant when desired.

## Supporting information

S1 Table (DOCX)

S2 Table (PDF)

S3 Table (DOCX)

S4 Table (XLSX)

S5 Table (PDF)

S6 Table (PDF)

S7 Table (PDF)
