# Supplementary material for: Marked reduction in fertility among African women with urogenital infections: A prospective cohort study
Source: PLoS One. 2019 Jan 10;14(1):e0210421. doi: 10.1371/journal.pone.0210421 (PMC6328149; doi:10.1371/journal.pone.0210421)
Supplement: S1 Table — (DOCX) [file pone.0210421.s001.docx]

| **Characteristic- 360 days follow up** | **Interval** | **OR** | **95%CI** | **p-value** |
| --- | --- | --- | --- | --- |
| Age | 18-25 | Ref re |  |  |
|  | 25-30 | 0.66 | 0.37-1.16 | 0.14 |
|  | 30-35 | 0.54 | 0.29-0.99 | 0.049* |
|  | 35-40 | 0.34 | 0.19-0.60 | <0.001*** |
| Partner status | No partner | Ref |  |  |
|  | Living with partner | 3.79 | 1.91-7.98 | <0.001*** |
|  | Partner - not living together | 2.18 | 0.93-5.29 | 0.076 |
| Urogenital infection | No | Ref |  |  |
|  | Yes | 0.19 | 0.10-0.32 | <0.001*** |
| Terminated hormonal contraception | Never used | Ref |  |  |
|  | <3 months | 3.78 | 1.62-9.54 | 0.003** |
|  | 3-6 months | 1.47 | 0.71-3.03 | 0.29 |
|  | 6-12 months | 0.93 | 0.46-1.90 | 0.86 |
|  | > 12 months | 1.31 | 0.77-2.25 | 0.32 |
|  | Used, term date unknown | 1.03 | 0.49-2.09 | 0.93 |

| **Characteristic – 90 days follow up** | **Interval** | **OR** | **95%CI** | **p-value** |
| --- | --- | --- | --- | --- |
| Age | 18-25 | Ref re |  |  |
|  | 25-30 | 0.89 | 0.51-1.53 | 0.67 |
|  | 30-35 | 0.54 | 0.29-1.00 | 0.05* |
|  | 35-40 | 0.35 | 0.19-0.64 | 0.001** |
| Partner status | No partner | Ref |  |  |
|  | Living with partner | 6.46 | 2.57-21.8 | <0.001*** |
|  | Partner - not living together | 3.23 | 1.03-12.2 | 0.06 |
| Urogenital infection | No | Ref |  |  |
|  | Yes | 0.09 | 0.02-0.27 | <0.001*** |
| House roof | Corrugated iron | Ref |  |  |
|  | Not- Corrugated iron | 1.71 | 1.02-2.7 | 0.02* |
