# Supplementary material for: Marked reduction in fertility among African women with urogenital infections: A prospective cohort study
Source: PLoS One. 2019 Jan 10;14(1):e0210421. doi: 10.1371/journal.pone.0210421 (PMC6328149; doi:10.1371/journal.pone.0210421)
Supplement: S2 Table — (PDF) [file pone.0210421.s002.pdf]

Parameters	Interval	Non-preg	Preg	P-value
Hip	70-79.9	12	2	0,48
	80-89.9	143	57	
	90-99.9	217	81	
	100-109.9	105	43	
	>110	42	24	
Waist	<70	57	23	1,00
	70-80	200	78	
	80-90	143	58	
	90-100	77	31	
	>100	38	16	
Mid upper arm circumference	<23	23	9	0,80
	23-25	71	23	
	25-27	105	46	
	>27	327	130	
Religion	Islamic	379	156	0,88
	Christian	60	22	
	Other	81	30	
Toilet	Flush	111	48	0,74
	Pit Latrin	410	158	
	No toilet	6	3	
Job – use of chemicals	Yes	9	4	0,77
	No	518	205	
Hypertension	Yes	47	17	0,88
	No	480	192	
Diabetes	Yes	6	1	0,68
	No	521	208	
Male age	<25	24	12	0,30
	25-45	224	112	
	>45	54	17	
Male education	Non	13	7	0,86
	Primary school partly/fully completed	302	144	
	Secondary school and higher	70	30	
Male religion	Islamic	300	142	0,88
	Christian	54	24	
	Other	49	20	
