# Supplementary material for: Marked reduction in fertility among African women with urogenital infections: A prospective cohort study
Source: PLoS One. 2019 Jan 10;14(1):e0210421. doi: 10.1371/journal.pone.0210421 (PMC6328149; doi:10.1371/journal.pone.0210421)
Supplement: S3 Table — (DOCX) [file pone.0210421.s003.docx]

| **Characteristic** | **Interval** | **OR** | **95%CI** | **p-value** |
| --- | --- | --- | --- | --- |
| Age |  | 0.95 | 0.92-0.97 | <0.001*** |
| Partner status | No partner | Ref |  |  |
|  | Living with partner | 3.68 | 1.90-7.69 | <0.001*** |
|  | Partner - not living together | 1.96 | 0.85-4.65 | 0.12 |
| Urogenital infection*** | No | Ref |  |  |
|  | Yes | 0.21 | 0.11-0.36 | <0.001*** |
| House roof | Corrugated iron | Ref |  |  |
|  | Not Corrugated iron | 1.54 | 1.03-2.30 | 0.036* |
| Terminated hormonal contraception | Never used | Ref |  |  |
|  | <3 months | 2.76 | 1.41-5.46 | 0.003** |
|  | 3-6 months | 1.52 | 0.79-2.90 | 0.21 |
|  | 6-12 months | 1.30 | 0.66-2.53 | 0.45 |
|  | > 12 months | 1.11 | 0.67-1.85 | 0.69 |
|  | Used, term date unknown | 0.93 | 0.46-1.80 | 0.83 |
