# Supplementary material for: Marked reduction in fertility among African women with urogenital infections: A prospective cohort study
Source: PLoS One. 2019 Jan 10;14(1):e0210421. doi: 10.1371/journal.pone.0210421 (PMC6328149; doi:10.1371/journal.pone.0210421)
Supplement: S5 Table — (PDF) [file pone.0210421.s005.pdf]

FOETALforNCD – FOetal Exposure and Epidemiological Transitions: the role of Anaemia in early Life for Non-Communicable Diseases in later life

Surveillance forms
3RD MONTHLY VISIT/INVESTIGATION OF SUSPECTED PREGNANCY

1. Clinic location:
☐ Korogwe District Hospital
☐ Ngombezi Dispensary
☐ Majengo Dispensary
☐ Hale Dispensary
☐ Chekelei Dispensary
☐ Kerenge Dispensary
☐ Lwengera Dispensary
☐ Segera Dispensary
☐ Makuyuni Dispensary
☐ Other

1.1.1. If other, specify:____

1.2. **Name of study worker filling q.1-1.10, and q. 1.13-1.14)**:____________________

1.3. Date of filling CRF(*when filling of CRF is started)*: (dd/mm/yyyy) _ _ / _ _ / _ _ _ _

1.4. Woman's surname: ____

1.5. Woman's first and second name: _________________________________

1.6. Type of visit ☐ 3rd monthly visit ☐ Reported herself

1.6.1. Number of this type of visit (incl. today) _ _

1.7. **Pregnancy test (urine) positive** ☐ yes ☐ no

If POSITIVE on pregnancy test fill Inclusion forms in Pregnancy CRF for the cohort study, if NEGATIVE continue with question 1.8

1.8. **Name of study worker filling anthropometry:** ____________________________

1.9. **Number of menstrual periods since last visit** _ _ ☐ Don't know

1.10. Blood sample collected today ☐ yes ☐ no

1.11. **BASELINE HEALTH DATA:**

1.11.1. Hemoglobin level at today's visit (on hemocue machine) (g/dL) _ _ , _

1.11.2. MUAC (cm) _ _ , _

1.11.3. Weight (kg) _ _ _ , _

1.11.4. Waist circumference (cm) (at the top of iliac crest) _ _ _ , _

1.11.5. Hip circumference (cm) (widest portion of the buttocks) _ _ _ , _

HEALTH STATUS today:

1.12. Any illness diagnosed today: ☐ yes ☐ no

1.12.1. If yes, state diagnose: ____

1.12.2. If yes, state all symptoms reported:

____
____
____

1.12.3. If yes, state all clinical findings:

____
____
____

1.12.4. If yes, state treatment prescribed (name of drug, dose, duration of treatment):

____
____
____

1.13. Informed to report in 3 months/if she suspects she is pregnant? ☐ yes ☐ no

DATA ENTRY:

1st entry done by:__________________________Signature:_____________ date:_ _/_ _/_ _ _ _

2nd entry done by:__________________________Signature:_____________ date:_ _/_ _/_ _ _ _
