# Supplementary material for: Marked reduction in fertility among African women with urogenital infections: A prospective cohort study
Source: PLoS One. 2019 Jan 10;14(1):e0210421. doi: 10.1371/journal.pone.0210421 (PMC6328149; doi:10.1371/journal.pone.0210421)
Supplement: S6 Table — (PDF) [file pone.0210421.s006.pdf]

FOETALforNCD – FOetal Exposure and Epidemiological Transitions: the role of Anaemia in early Life for Non-Communicable Diseases in later life

Inclusion form 1, Cohort Study Pre-preg

1. Clinic location:

☐ Korogwe District Hospital ☐ Kerenge Dispensary
☐ Ngombezi Dispensary ☐ Lwengera Dispensary
☐ Majengo Dispensary ☐ Segera Dispensary
☐ Hale Dispensary ☐ Makuyuni Dispensary
☐ Chekelei Dispensary ☐ Other

1.1.1. If other, specify:____
1.2. **Name of field worker/project nurse filling the form (q.1-1.9.10)**:_______________________
1.3. **Name of project nurse filling the form (anthropometry):** ____________________________
1.4. Date of filling CRF(*when filling of CRF is started*): (dd/mm/yyyy) _ _ / _ _ / _ _ _ _
1.5. **Woman's surname**: ______________________
1.6. **Woman's first and second name**: ____
1.7. **Woman's age:** _ _ years ☐ unknown
1.8. Date of birth according to the woman (dd/mm/yyyy) _ _ / _ _ / _ _ _ _
1.8.1. If age/birthday not know, thought to be 18-40 years by interviewer ☐ yes ☐ no

1.9. **ADDRESS**
1.9.1. Street name: ____
1.9.2. Street Chairman: ____
1.9.3. Village: ____
1.9.4. Subvillage: ____
1.9.5. Subvillage chair: ____
1.9.6. District/ward: ____
1.9.7. Ten Cell Leader: ____
1.9.8. Name of Husband/partner if living together:___________________________________
1.9.9. Head of household:____
1.9.9.1. House number:____________________________
1.9.9.2. Known as Mama:___________________________
1.9.10. Woman's or partners Phone number:____

1.10. **BASELINE HEALTH DATA:**

1.10.1.	Hemoglobin level at today's visit (on hemocue machine) (g/dL)	_ _ , _
1.10.2.	MUAC (cm)	_ _ , _
1.10.3.	Height (cm)	_ _ _
1.10.4.	Weight (kg)	_ _ _ , _
1.10.5.	Waist circumference (cm) (at the top of the iliac crest)	_ _ _ , _
1.10.6.	Hip circumference (cm) (at widest portion of the buttocks)	_ _ _ , _
1.10.7.	Pregnancy test (urine) positive	☐ yes ☐ no ☐ not done

1.11. **ELIGIBILITY FOR COHORT study**

1.11.1.	Previously screened for/ included in PONA Cohort	☐ yes ☐ **no**	Exclusion if yes
1.11.2.	Current use of modern family planning	☐ yes ☐ **no**	Exclusion if yes
1.11.3.	Negative pregnancy test	☐ **yes** ☐ no	Exclusion if no
1.11.4.	Wish to become pregnant in near future	☐ **yes** ☐ no	Exclusion if no
1.11.5.	Age between 18 – 40 years	☐ **yes** ☐ no	Exclusion if no
1.11.6.	Informed about pregnancy component	☐ **yes** ☐ no	
1.11.7.	Informed about and consents to participate in Cohort	☐ **yes** ☐ no	Exclusion if no
1.11.8.	Children<9months	☐ yes ☐ **no**	Exclusion if yes
1.11.9.	Tried to become pregnant for >2years	☐ yes ☐ **no**	Exclusion if yes

1.12. ☐ **INCLUSION** ☐ **REFUSAL** ☐ **EXCLUSION**
1.12.1. **In case of inclusion**, included as ☐ case (Hb≤8g/dL)☐ control (Hb>8g/dL) ☐ NA
1.12.2. **Excluded due to imbalance of 1:1 case-control design:** ☐ yes ☐ no ☐ NA
1.12.3. **In case of refusal or exclusion**, state reason: ____________________________
1.12.4. **State CASE-CONTROL ID number: FN _ _ _ _ _** ☐ Do not have

2. Woman Inclusion Form 2, Cohort Study Pre-preg

2.1. Name of study worker filling the form: ______________________________

2.2. Date of filling CRF (dd/mm/yyyy): _ _ / _ _ / _ _ _ _

MATERNAL DEMOGRAPHIC DATA

2.3. **Ethnic** group ☐ Sambaa ☐ Zigua ☐ Pare ☐ Bondei ☐ Other

2.3.1. if other, specify: ______________________________

2.4. **Residence**:

2.4.1. Type of **roof** on the house: ☐ Bati ☐ Tin(madebe) ☐Thatch ☐Mixed Thatch/Tin ☐ Other

2.4.1.1. if other, specify: ______________________________

2.4.2. How **many people** usually sleep in your residence _ _

2.4.3. Who **owns the house** you live in ☐ self/spouse built ☐ inherited ☐ rental ☐ others

2.4.3.1. if others, specify:______________________________

2.4.4. Type of **home toilet**: ☐ flush ☐ pit latrine(choo cha shimo) ☐ no toilet

2.4.5. Source of **water** ☐ tap(bombani) ☐ well (kisima (cha mdundiko) ☐ river/stream (mto) ☐ gutter water in tank (maji ya paa) ☐ pond/pool (bwawa) ☐ other

2.4.5.1. if other, specify: ______________________________

2.4.5.2. if tap, well, or gutter water ☐ Private ☐ Public

2.5. **Education** ☐ none ☐ primary school partially completed ☐ primary school finished ☐ secondary school and higher

2.5.1. **Do you know how to read and write** ☐ yes ☐ no

2.6. **Current occupation** ☐ Professional ☐ Business ☐ Service ☐ Farmer ☐ Housewife/working at home ☐ Other

2.6.1. If other, specify: ______________________________

2.6.2. **Do you use chemicals** for pest/diseases in your work ☐ yes ☐ no

2.6.2.1. If yes, specify:______________________________

2.7. **No. of siblings** (genetic brothers and sisters, same mother/father) _ _ ☐ unknown

2.8. **Residence before age 15** for the majority of the time ☐urban ☐rural ☐urban/rural ☐ unknown

2.9. **Marital status** ☐ Married ☐ Divorced ☐ Separated ☐ Never married ☐ Widow ☐ Refuse answer

2.9.1. If divorced/separated/widow/never married, do you currently have a partner ☐ yes, cohabiting ☐ yes, but not cohabiting ☐ no

2.10. **Religion** ☐ Islamic ☐Catholic ☐ Lutheran ☐ Angikana ☐ Hindu ☐Other

2.10.1. If other, specify:______________________________

DEMOGRAPHIC DATA ON PARTNER (answer if married or having a partner)

2.11. Agree to answer questions about the partner ☐ yes ☐ no

2.12. **Name** of partner:______________________________

2.13. **Age** of partner _ _ years ☐ unknown

2.14. **Ethnic** group ☐ Sambaa ☐ Zigua ☐ Para ☐ Bondei ☐ Other ☐ unknown

2.14.1. if other, specify: ______________________________

2.15. **Education** ☐ none ☐ primary school partially completed ☐ primary school finished ☐ ≥secondary school ☐ unknown

2.15.1. **Does he knows how to read and write** ☐ yes ☐ no ☐ unknown

2.16. **Current occupation** ☐ Professional ☐ Business ☐ Service ☐ Farmer ☐ Other ☐ unknown

2.16.1. If other, specify:______________________________

2.17. **Religion** ☐Islamic ☐Catholic ☐ Lutheran ☐ Angikana ☐ Hindu ☐Other ☐ unknown

2.17.1. if other, specify:______________________________

2.18. **GRAVIDITY:**
2.18.1. Number of pregnancies (gravidae) _ _
2.18.2. Number of deliveries (parity) _ _
2.18.2.1. Number of times delivering twins _ _
2.18.2.2. Nb. of live births (nb of babies born; singleton=1 & twins=2 if both live born) _ _
2.18.2.3. Nb. of still births (nb of babies born; singleton=1 & twins=2 if both stillborn) _ _
2.18.3. Number of interrupted pregnancies (miscarriages) _ _
2.18.4. Number of extrauterine pregnancies (mimba nje ya kizazi) _ _
2.18.5. Date of termination the last pregnancy (incl. delivery, miscarriage, extrauterine)
(dd/mm/yyyy) _ _ /_ _ /_ _ _ _
2.18.6. Date of last delivery (dd/mm/yyyy) _ _ /_ _ /_ _ _ _

NUTRITIONAL AND PHYSICAL HABITS
2.19. Has the “IPAQ questionnaire” been filled ☐ yes ☐ no
2.20. Has the “24hours Recall questionnaire” been filled ☐ yes ☐ no

MEDICAL HISTORY
Previous pregnancies (skip if never been pregnant)
2.21. Maternal disease during previous pregnancies, diagnosed by medical personnel
☐ preeclampsia (dalili za kifafa cha mimba)
☐ pregnancy-induced HT(shinikizo la damu linalotokana na ujauzito)
☐ diabetes (kisukari) ☐ other ☐ severe anaemia (upungufu wa damu kali)
☐ none ☐ Don’t know

2.21.1. If other, specify:____

Menstrual pattern and family planning
2.22. **Age of menarche** (first menstrual period/kuvunja ungo) _ _ ☐ unknown
2.23. **Having her menstrual period today** ☐ yes ☐ no
2.23.1. If Yes, date it started; if no date of last menstrual period(dd/mm/yyyy) _ _ /_ _/ _ _ _ _
2.23.2. Currently having amenorrhea ☐ yes ☐ no
2.23.2.1. If yes, state reason (e.g. since last delivery, recent breast-feeding, just stopped oral contraceptives/remove implant/recent use of injectable):__________________________
____

If having amenorrhea, answer question 2.24-2.26.2 according to menstrual pattern before current amenorrhea

2.24. **Length** of cycle (days) min_ _ _ max_ _ _ ☐ unknown
2.25. **Regular** period (max variation 20days) ☐ yes ☐ no ☐ unknown
2.26. **Days of bleeding** _ _ ☐ unknown
2.26.1. Degree of bleeding day 1 and 2
☐ mild (change pads 1-2 times per day)
☐ moderate (change pads 3-5 times per day)
☐ severe (change pads ≥6 times per day)
☐ Don’t know
2.26.2. Degree of bleeding remaining days
☐ mild (change pads 1-2 times per day)
☐ moderate (change pads 3-5 times per day)
☐ severe (change pads ≥6 times per day)
☐ Don’t know

2.27. **Which methods of family planning have you previously used** (x≥1 if need)
☐ oral contraceptive (vidonge) ☐ condom
☐ hormone injectable (sindano) ☐ implant (kijiti)
☐ periodic abstinence ☐ withdrawal
☐ IUD (kitanzi) ☐ none ☐ other
2.27.1. If other; specify:____

2.27.2. **Used more than one method of family planning** ☐ yes ☐ no

2.27.2.1. If yes, state the most recent modern method:
☐ oral contraceptive (vidonge) ☐ condom ☐ hormone injectable (sindano)
☐ implant (kijiti) ☐ IUD (kitanzi) ☐ other ☐ none

2.27.2.2. If yes, state the most recent traditional method:
☐ periodic abstinence ☐ withdrawal ☐ other ☐ none

2.27.3. **When did you terminate** using the most recent **modern method**_ _ / _ _ / _ _ _ _ ☐ UN
☐ still using (condoms only)

2.27.3.1. **When did you terminate** using the most recent **tradit. method**_ _ / _ _ / _ _ _ _ ☐ UN
☐ still using

If used oral contraceptives, hormone injectable, implant, IUD:

2.27.4. For how long have you used the method _ _ years _ _ months _ _ weeks
2.27.4.1. State method: ________________________
2.27.5. For how long have you used the method _ _ years _ _ months _ _ weeks
2.27.5.1. State method: ________________________
2.27.6. For how long have you used the method _ _ years _ _ months _ _ weeks
2.27.6.1. State method: ________________________
2.27.7. Notes on family planning/menstrual pattern:___________________________________
____
____

2.28. **Currently tried to get pregnant** for how long _ _ years _ _ months _ _ weeks ☐ unknown

2.29. **How many children** do you prefer/would you like to have (ungependa kuwa na watoto wangapi)
_ _ ☐ don't know/refuse to answer

2.30. **Who decide the number** of children you can have
☐ Me and my partner ☐ Me, alone
☐ My partner, alone ☐ Other

2.30.1. If other, specify:____

Anaemia

2.31. **Has chronic anaemia** (upungufu sugu) now (diagnosed by medical staff)
☐ yes, known irreversible cause
☐ yes, lasting>6months
☐ no
☐ don't know/not tested

2.31.1. If yes, cause known ☐ yes ☐ no
2.31.2. If yes, specify (e.g. sickle cell, severe malnutrition)________________________________

2.32. **Temporary anaemia diagnosed within the last 2 months** ☐ yes ☐ no
2.32.1. Nb. of times last 2 months _ _
2.32.2. If yes, state first day of the last episode (dd/mm/yyyy) _ _/_ _/_ _ _ _
2.32.2.1. Hemoglobin measurement done ☐ yes ☐ no
2.32.2.2. If yes, state the value (g/dL) _ _, _ ☐ unknown

2.33. **Received medicine for anaemia the last 2 months** ☐ yes ☐ no
2.33.1. If yes, specify(put >1 if needed): ☐ Iron ☐ Folic
☐ B12 ☐ Anti-helminths
☐ Hemovit ☐ Other
2.33.1.1. If other, specify______________________________________
2.33.2. State when treatment was started _ _ /_ _ /_ _ _ _
2.33.3. No. of tablets/day _ _ ☐ unknown
2.33.4. Dose per tablet _ _ _ _ ☐ unknown
2.33.5. State how long treatment was received: _ _ months _ _ weeks _ _ days☐ unknown
2.33.6. Additional notes:____

Other chronic diseases

2.34. Diagnosed with a chronic illness by medical personnel (ask specifically about: diabetes type I or II (kisukari), kidney (figo), heart (moyo), thyroid (goiter) or lung (mapafu) disease, cancer (saratani), hypertension (shinikizo la damu), epilepsy (kifafa), lymphatic filariasis (matende), gastric ulcer (ulcers/vidonda vya tumbo), autoimmune disorders (e.g. rheumatoid arthritis (rheumotism), chronic diarrhea (kuharisha kwa muda mrefu)
☐ yes ☐ no

2.34.1. if yes, specify____

2.35. **Any close relatives** (genetic sister, brother, father, mother, grandparents, aunt, uncle) with (>1 x if needed)
☐ Diabetes ☐ Cardiac disease
☐ Hypertension
☐ Severe undernutrition (utapiamlo mkali)
☐ Chronic anaemia (e.g. sickle cell, thalassemia)
☐ Other ☐ No known disease

2.35.1. If yes or other, specify which relative and disease________________________________
____

2.36. **Ever diagnosed with a pelvic inflammatory disease** by medical personnel
☐ yes ☐ no

2.37. **Diagnosed with any gynecological disorder** (problem with ovary or uterus) by medical personnel (e.g. endometriosis, fibroma, cysts on the ovary, septum in uterus)
☐ yes ☐ no

2.37.1. If yes, specify:____

2.38. **Ever had abdominal surgery performed** (surgery for appendicitis (kidole tumbo), gall bladder stone (mawe kwenye kibofu cha nyongo), uterus fibroma, ovary cysts, caesarean (kujifungua kwa upasuaji)) ☐ yes ☐ no

2.38.1. If yes, specify:____

2.39. **HIV status** according to the woman:
☐ Unwilling to respond
☐ Positive
☐ Negative, will be re-testing today
☐ Negative, do not want re-testing
☐ Do not know, will be tested today
☐ Do not know, do not want testing

2.39.1. If positive, attending a CTC ☐ yes ☐ no

2.39.1.1. If yes, where:____

Malaria

2.40. **Does she have a bednet yes/no** ☐ yes ☐ no
2.40.1. If yes, did she use it last night? ☐ yes ☐ no
2.40.2. Impregnated bednet ☐ yes ☐ no ☐ unknown
2.40.3. Obtained from national programme ☐ yes ☐ no ☐ unknown

2.41. **Nb. of malaria attacks** during the last 2 months? ☐ 0 ☐ 1 ☐ 2 ☐ 3 ☐ other
2.41.1. If other number, specify: ___
2.41.2. Date of the 1st malaria attack: (dd/mm/yyyy) _ _ / _ _ / _ _ _ _
2.41.3. Date of the 2nd malaria attack:(dd/mm/yyyy) _ _ / _ _ / _ _ _ _
2.41.4. Date of the 3rd malaria attack: (dd/mm/yyyy) _ _ / _ _ / _ _ _ _

2.42. **1st malaria attack:**
2.42.1. Was malaria confirmed with a blood test ☐ yes ☐ no ☐ unknown
2.42.2. What treatment did you take? ☐ Quinine ☐ SP/Metakelfin
☐ Chloroquine ☐ Herbal remedy
☐ None ☐ unknown ☐ other ☐ ALU
2.42.2.1. If other, details: ____

2.43. **2nd malaria attack:**
2.43.1. Was malaria confirmed with a blood test ☐ yes ☐ no ☐ unknown
2.43.2. What treatment did you take? ☐ Quinine ☐ SP/Metakelfin
☐ Chloroquine ☐ Herbal remedy
☐ None ☐ unknown ☐ other ☐ ALU
2.43.2.1. If other, details: ____

2.44. **3rd malaria attack:**
2.44.1. Was malaria confirmed with a blood test ☐ yes ☐ no ☐ unknown
2.44.2. What treatment did you take? ☐ Quinine ☐ SP/Metakelfin
☐ Chloroquine ☐ Herbal remedy
☐ None ☐ unknown ☐ other ☐ ALU
2.44.2.1. If other, details: ____

Current usage of medicine

2.45. Currently taking medication? ☐ yes ☐ no

2.45.1. If yes, give details (≥1 "x"): ☐ antibiotics ☐ antimalarial ☐ iron ☐ antiretrovirals ☐ folic acid ☐ B12 ☐ antihelminths ☐ traditional ☐ Hemovit ☐ antihypertensive ☐ painkillers ☐ other

2.45.1.1. If other or painkillers,specify: ____

2.45.1.2. If, traditional specify: ____

2.45.1.3. Dosage of these drugs: ____

____

____

Substance abuse:

2.46. Smoker ☐ yes ☐ no

2.46.1. If yes, number of cigarettes per _ _ month _ _ week _ _ day

2.47. Usage of alcohol ☐ yes ☐ no

2.47.1. If yes, how many items per _ _ month _ _ week _ _ day

2.48. Usage of caffeine beverages ☐ yes ☐ no

2.48.1. If Coke, how many items (bottles) per _ _ month _ _ week _ _ day

2.48.2. If coffee, how many items (cups) per _ _ month _ _ week _ _ day

2.48.3. If tea, how many item (cups) per _ _ month _ _ week _ _ day

2.48.4. NOTES on substance abuse:____

____

____

MEDICAL EXAMINATION *(all * should be filled by CO/AMO/MD)*

2.49. Skinfold thickness of triceps (mm) _ _ , _

2.50. Blood pressure (BP) (mmHg)

2.50.1. 1st BP, left arm _ _ _ / _ _ _

2.50.2. 1st BP, right arm _ _ _ / _ _ _

2.50.3. Difference in left and right arm (max 20 syst.; 10 diast.) _ _ / _ _

2.50.4. Reference arm (the arm with the highest BP) ☐ Right ☐ Left

2.50.5. Reference arm (the arm with the highest BP), 2nd BP _ _ _ / _ _ _

2.50.6. Mean BP (1st and 2nd BP for reference arm) _ _ _ / _ _ _

2.50.7. Pulse (use last BP measurement) _ _ _

2.51. Axillary temperature (°C) _ _ , _

2.52. * Headache ☐ yes ☐ no

2.53. * Visual disturbances ☐ yes ☐ no

2.53.1. If yes, specify: ____

2.54. * Dizziness ☐ yes ☐ no

2.55. * Denuded/glossy tongue (glossitis) ☐ yes ☐ no

2.56. * Commisura of the lips (cheilosis, fissure) ☐ yes ☐ no

2.57. * Vitiligo ☐ yes ☐ no

2.58. * Pallor (conjunctivae or palms of hands) ☐ yes ☐ no

2.59. * Deformities of the nails (flattening/koilonychia) ☐ yes ☐ no

2.60. * Pitting oedema (swelling of lower/upper limps or/and face) ☐ yes ☐ no

2.61. * Heart palpitations ☐ yes ☐ no

2.62. * Angina pectoris ☐ yes ☐ no

2.63. * Cardiac murmurs ☐ yes ☐ no

2.64. * Dyspnea ☐ yes ☐ no

2.65. * Pulmonary stethoscopic signs of abnormalities ☐ yes ☐ no

2.65.1. If yes, specify:____

2.66. * Nausea and/or vomiting ☐ yes ☐ no

2.67. * Hematemesis ☐ yes ☐ no

2.68. * Severe epigastric pain ☐ yes ☐ no

2.69. * Enlarged spleen ☐ yes ☐ no

2.70. * Melena ☐ yes ☐ no

2.71. * Other symptoms/clinical findings ☐ yes ☐ no

2.71.1. If yes, specify:____

SAMPLES COLLECTED

2.72.	**Malaria RDT**	☐ negative	☐ PF	☐ PAN	☐ PF+PAN	☐ not done		
2.73.	**HIV RDT**		☐ negative	☐ positive	☐ not done			
2.74.	**Venous blood draw**		☐ done	☐ not done				
2.74.1.	if not done, why :		☐ refusal	☐ failure	☐ forgot			
2.75.	CPDA Tube :		☐ done	☐ not done	☐ not applicable			
2.76.	EDTA Tube (6ml + 2ml):		☐ done	☐ not done				
2.77.	Plain Tube (6ml + eppendorf):		☐ done	☐ not done				
2.78.	**Blood sugar**		_ _ , _	☐ not done				
2.78.1.	If measured, used		☐ fingerprick	☐ venous blood				
2.79.	HbA1C		_ _ , _	☐ not done				

2.80. **Urine dipstick**

2.80.1.	Albumin in the urine	☐ 0+	☐ 1+	☐ 2+	☐ 3+			☐ not done
2.80.2.	Sugar in the urine	☐ 0+	☐ 1+	☐ 2+	☐ 3+	☐ 4+	☐ 5+	☐ not done
2.80.3.	Leucocytes in the urine	☐ 0+	☐ 1+	☐ 2+	☐ 3+			☐ not done
2.80.4.	Blood in urine	☐ 0+	☐ 1+	☐ 2+	☐ 3+			☐ not done
2.80.5.	Ketones	☐ 0+	☐ 1+	☐ 2+	☐ 3+			☐ not done
2.80.6.	Nitrite	☐ 0+	☐ 1+	☐ 2+				☐ not done

CONCLUSION ON TODAY’S EXAMINATION

2.81. **Disease diagnosed today** ☐ yes ☐ no

2.81.1. If yes, specify (≥1 “x”)
☐ Anaemia ☐ Malaria ☐ Suspected hypertension
☐ Urinary tract infection ☐ Diabetes
☐ Upper respiratory tract infect. ☐ Syphilis ☐ HIV
☐ Reproductive tract infection ☐ Other

2.81.1.1. If other, specify:______________________________________

2.82. **Medicine prescribed today** ☐ yes ☐ no

2.82.1. If yes, specify (≥1 “x”)
☐ Coartem/ALU ☐ Quinine ☐ anti-helminth
☐ Iron ☐ Folic acid ☐ B12 ☐ Hemovit
☐ Antibiotics ☐ Anti-HT ☐ Painkillers ☐ Other

2.82.1.1. If painkillers or other, specify: ______________________________

2.82.2. Specify name, dosage and duration of treatment: ______________________
____

2.83. **Based on Sysmex results ☐ case (Hb≤8g/dL) ☐ control (Hb>8g/dL) ☐ Not applicable**

2.84. **NOTES:**____
____
____
____
____

Questions marked * filled in by: ______________________________ Signature:______________

DATA ENTRY:

1st entry done by:_________________________________Signature:______________ date:_ _/_ _/_ _ _ _

2nd entry done by:_________________________________Signature:______________ date:_ _/_ _/_ _ _ _

Identification No. : F N _ _ _ _ _ _ _ _ **Version 6 8MAR2015**

3. EXCLUSION FORM Cohort Study Pre-PREG

3.1. Clinic location:

☐ Korogwe District Hospital ☐ Kerenge Dispensary
☐ Ngombezi Dispensary ☐ Lwengera Dispensary
☐ Majengo Dispensary ☐ Segera Dispensary
☐ Hale Dispensary ☐ Makuyuni Dispensary
☐ Chekelei Dispensary ☐ Other

3.1.1. If other, specify :________________________________

3.2. Date : _ _ / _ _ / _ _ _ _ (dd/mm/yyyy)

3.3. Completed by : ____

3.4. EXCLUSION DUE TO :

☐ Moving out of the study area ☐ Lost to follow-up
☐ Refusal/withdraw of consent ☐ Other

3.5. Explanatory notes : ____

____
____
____
____
____
____
____
____

DATA ENTRY:

1st entry done by:____Signature:________________ date:_ _/_ _/_ _ _ _

2nd entry done by:____Signature:________________ date:_ _/_ _/_ _ _ _

Identification No. : F N _ _ _ _ _ _ _ _ **Version 6 8MAR2015**

4. EXTRA VISIT FORM Cohort Study Pre-Preg

4.1. Clinic location:
☐ Korogwe District Hospital
☐ Kerenge Dispensary
☐ Ngombezi Dispensary
☐ Lwengera Dispensary
☐ Majengo Dispensary
☐ Segera Dispensary
☐ Hale Dispensary
☐ Makuyuni Dispensary
☐ Chekelei Dispensary
☐ Other

4.1.1. If other, specify :_________________________________

4.2. Date : _ _ / _ _ / _ _ _ _ (dd/mm/yyyy)

4.2.1. **Number of this type of visit : _ _**

4.3. Completed by : ____

4.4. Extra visit due to:
☐ BP ≥140/90 on inclusion
☐ Glucose ≥11.1mmol/L on inclusion
☐ Other

4.5. BP today (mmHg) on reference arm (the one with the highest BP) :

4.5.1. 1st _ _ _ / _ _ _

4.5.2. 2nd _ _ _ / _ _ _

4.5.3. Mean _ _ _ / _ _ _

4.6. Glucose level today (mmol/L): _ _ , _

4.7. Notes : ____
____
____
____
____
____
____
____
____

DATA ENTRY:

1st entry done by:____Signature:_________________ date:_ _/_ _/_ _ _ _

2nd entry done by:____Signature:_________________ date:_ _/_ _/_ _ _ _

5. BLOOD SAMPLE FORM Cohort Study Pre-Preg

For women having their first 3rd Monthly visit : *This form should be filled for all women previously screened for the pre-pregnancy study, where a complete CRF was filled, but no blood sample was collected. If the woman was screened, but only form 1 was filled (ID≤112 & 200-208) fill a form 2 instead. For all women remember to fill at 3rd Monthly visit form.*

5.1. Clinic location:
- ☐ Korogwe District Hospital
- ☐ Kerenge Dispensary
- ☐ Ngombezi Dispensary
- ☐ Lwengera Dispensary
- ☐ Majengo Dispensary
- ☐ Segera Dispensary
- ☐ Hale Dispensary
- ☐ Makuyuni Dispensary
- ☐ Chekelei Dispensary
- ☐ Other

5.1.1. If other, specify :____

5.2. Date : _ _ / _ _ / _ _ _ _ (dd/mm/yyyy)

5.2.1.1. **Type of visit:**
- ☐ First 3rd monthly for screened in old strategy [7]
- ☐ Normal 3rd monthly [8]
- ☐ Self-reported UPT visit [9]
- ☐ Extra visit (form 4) [11]

5.2.2. **Nb. of this type of visit:** _ _

5.3. Completed by : ____

5.4. 3rd monthly visit form filled ☐ yes ☐ no

CURRENT MEDICAL HISTORY

Recent Anaemia

5.5. **Temporary anaemia diagnosed** within the last 2 months ☐ yes ☐ no

5.5.1. Nb. of times last 2 months _ _

5.5.2. If yes, state first day of the last episode (dd/mm/yyyy) _ _/_ _/_ _ _ _

5.5.2.1. Hemoglobin measurement done ☐ yes ☐ no

5.5.2.2. If yes, state the value (g/dL) _ _, _ ☐ unknown

5.6. **Received medicine for anaemia the last 2 months** ☐ yes ☐ no

5.6.1. If yes, specify(put >1 if needed): ☐ Iron ☐ Folic ☐ B12 ☐ Anti-helminths ☐ Hemovit ☐ Other

5.6.1.1. If other, specify____

5.6.2. State when treatment was started _ _ /_ _ /_ _ _ _

5.6.3. No. of tablets/day _ _ ☐ unknown

5.6.4. Dose per tablet _ _ _ _ ☐ unknown

5.6.5. State how long treatment was received: _ _ months _ _ weeks _ _ days☐ unknown

5.6.6. Additional notes:____

Other current disease

5.7. **HIV status** according to the woman:
- ☐ Unwilling to respond
- ☐ Positive
- ☐ Negative, will be re-testing today
- ☐ Negative, do not want re-testing
- ☐ Do not know, will be tested today
- ☐ Do not know, do not want testing

5.7.1. If positive, attending a CTC ☐ yes ☐ no

5.7.1.1. If yes, where:____

Malaria

5.8. **Does she have a bednet** ☐ yes ☐ no
5.8.1. If yes, did she use it last night? ☐ yes ☐ no
5.8.2. Impregnated bednet ☐ yes ☐ no ☐ unknown
5.8.3. Obtained from national programme ☐ yes ☐ no ☐ unknown

5.9. **Nb. of malaria attacks** during the last 2 months? ☐ 0 ☐ 1 ☐ 2 ☐ 3 ☐ other
5.9.1. If other number, specify: ___
5.9.2. Date of the 1st malaria attack: (dd/mm/yyyy) _ _ / _ _ / _ _ _ _
5.9.3. Date of the 2nd malaria attack:(dd/mm/yyyy) _ _ / _ _ / _ _ _ _
5.9.4. Date of the 3rd malaria attack: (dd/mm/yyyy) _ _ / _ _ / _ _ _ _

5.10. **1st malaria attack:**
5.10.1. Was malaria confirmed with a blood test ☐ yes ☐ no ☐ unknown
5.10.2. What treatment did you take? ☐ Quinine ☐ SP/Metakelfin ☐ Chloroquine ☐ Herbal remedy ☐ None ☐ unknown ☐ other ☐ ALU
5.10.2.1. If other, details: ____

5.11. **2nd malaria attack:**
5.11.1. Was malaria confirmed with a blood test ☐ yes ☐ no ☐ unknown
5.11.2. What treatment did you take? ☐ Quinine ☐ SP/Metakelfin ☐ Chloroquine ☐ Herbal remedy ☐ None ☐ unknown ☐ other ☐ ALU
5.11.2.1. If other, details: ____

5.12. **3rd malaria attack:**
5.12.1. Was malaria confirmed with a blood test ☐ yes ☐ no ☐ unknown
5.12.2. What treatment did you take? ☐ Quinine ☐ SP/Metakelfin ☐ Chloroquine ☐ Herbal remedy ☐ None ☐ unknown ☐ other ☐ ALU
5.12.2.1. If other, details: ____

Current usage of medicine

5.13. Currently taking medication? ☐ yes ☐ no
5.13.1. If yes, give details (≥1 “x”): ☐ antibiotics ☐ antimalarial ☐ iron ☐ antiretrovirals ☐ folic acid ☐ B12 ☐ antihelminths ☐ traditional ☐ Hemovit ☐ antihypertensive ☐ painkillers ☐ other
5.13.1.1. If other or painkillers,specify: ______________________________
5.13.1.2. If, traditional specify: ______________________________
5.13.1.3. Dosage of these drugs: ______________________________
____
____

MEDICAL EXAMINATION (*all * should be filled by CO/AMO/MD*)

5.14. Blood pressure (BP) (mmHg)
5.14.1. 1st BP, left arm _ _ _ / _ _ _
5.14.2. 1st BP, right arm _ _ _ / _ _ _
5.14.3. Difference in left and right arm (max 20 syst.; 10 diast.) _ _ / _ _
5.14.4. Reference arm (the arm with the highest BP) ☐ Right ☐ Left
5.14.5. Reference arm (the arm with the highest BP), 2nd BP _ _ _ / _ _ _
5.14.6. Mean BP (1st and 2nd BP for reference arm) _ _ _ / _ _ _
5.14.7. Pulse (use last BP measurement) _ _ _
5.15. Axillary temperature (°C) _ _ , _
5.16. * Headache ☐ yes ☐ no
5.17. * Visual disturbances ☐ yes ☐ no
5.18. * Dizziness ☐ yes ☐ no

5.19. * Denuded/glossy tongue (glossitis) ☐ yes ☐ no
5.20. * Commisura of the lips (cheilosis, fissure) ☐ yes ☐ no
5.21. * Vitiligo ☐ yes ☐ no
5.22. * Pallor (conjunctivae or palms of hands) ☐ yes ☐ no
5.23. * Deformities of the nails (flattening/koilonychia) ☐ yes ☐ no
5.24. * Pitting oedema (swelling of lower/upper limps or/and face) ☐ yes ☐ no
5.25. * Heart palpitations ☐ yes ☐ no
5.26. * Angina pectoris ☐ yes ☐ no
5.27. * Cardiac murmurs ☐ yes ☐ no
5.28. * Dyspnea ☐ yes ☐ no
5.29. * Pulmonary stethoscopic signs of abnormalities ☐ yes ☐ no
5.29.1. If yes, specify:______________________________
5.30. * Nausea and/or vomiting ☐ yes ☐ no
5.31. * Hematemesis ☐ yes ☐ no
5.32. * Severe epigastric pain ☐ yes ☐ no
5.33. * Enlarged spleen ☐ yes ☐ no
5.34. * Melena ☐ yes ☐ no
5.35. * Other symptoms/clinical findings ☐ yes ☐ no
5.35.1. If yes, specify:______________________________

SAMPLES COLLECTED

5.36. **Malaria RDT** ☐ negative ☐ PF ☐ PAN ☐ PF+PAN ☐ not done
5.37. **HIV RDT** ☐ negative ☐ positive ☐ not done
5.38. **Venous blood** draw ☐ done ☐ not done
5.38.1. if not done, why : ☐ refusal ☐ failure ☐ forgot
5.39. CPDA Tube : ☐ done ☐ not done ☐ not applicable
5.40. EDTA Tube (6ml + 2ml): ☐ done ☐ not done
5.41. Plain Tube (6ml + eppendorf): ☐ done ☐ not done
5.42. **Blood sugar** _ _ , _ ☐ not done
5.42.1. If measured, used ☐ fingerprick ☐ venous blood
5.43. HbA1C _ _ , _ ☐ not done
5.44. **Urine dipstick**
5.44.1. Albumin in the urine ☐ 0+ ☐ 1+ ☐ 2+ ☐ 3+ ☐ not done
5.44.2. Sugar in the urine ☐ 0+ ☐ 1+ ☐ 2+ ☐ 3+ ☐ 4+ ☐ 5+ ☐ not done
5.44.3. Leucocytes in the urine ☐ 0+ ☐ 1+ ☐ 2+ ☐ 3+ ☐ not done
5.44.4. Blood in urine ☐ 0+ ☐ 1+ ☐ 2+ ☐ 3+ ☐ not done
5.44.5. Ketones ☐ 0+ ☐ 1+ ☐ 2+ ☐ 3+ ☐ not done
5.45. Nitrite ☐ 0+ ☐ 1+ ☐ 2+ ☐ not done

CONCLUSION ON TODAY'S EXAMINATION

5.46. **Disease diagnosed today** ☐ yes ☐ no
5.46.1. If yes, specify (≥1 "x") ☐ Anaemia ☐ Malaria ☐ Suspected hypertension
☐ Urinary tract infection ☐ Diabetes
☐ Upper respiratory tract infect. ☐ Syphilis ☐ HIV
☐ Reproductive tract infection ☐ Other
5.46.1.1. If other, specify:______________________________
5.47. **Treatment prescribed today** ☐ yes ☐ no
5.47.1. If yes, specify (≥1 "x") ☐ Coartem/ALU ☐ Quinine ☐ anti-helminth
☐ Iron ☐ Folic acid ☐ B12 ☐ Hemovit
☐ Antibiotics ☐ Anti-HT ☐ Painkillers ☐ Other
5.47.1.1. If painkillers or other, specify: ______________________________
5.47.2. Specify name, dosage and duration of treatment: ______________________________
____
____

5.48. Notes : ______________________________
____
