# Supplementary material for: Marked reduction in fertility among African women with urogenital infections: A prospective cohort study
Source: PLoS One. 2019 Jan 10;14(1):e0210421. doi: 10.1371/journal.pone.0210421 (PMC6328149; doi:10.1371/journal.pone.0210421)
Supplement: S7 Table — (PDF) [file pone.0210421.s007.pdf]

FOETALforNCD – FOetal Exposure and Epidemiological Transitions: the role of Anaemia in early Life for Non-Communicable Diseases in later life

Surveillance forms during Pregnancy, Cohort and Case-Control Study

Visits	Forms completed by :	Date:	Hb (g/dl)
Inclusion (*date pregnancy is confirmed with UPT)*	Nurse/Midwife (NMW) & Clinical Officer (CO)		
Antenatal visit (ANV) 2 - GA 20	NMW & CO		
ANV3 GA 26	NMW & CO		
ANV4 GA 32	NMW & CO		
ANV5 GA 37	NMW & CO		
Extra ANV N°	NMW & CO		
Extra ANV N°	NMW & CO		
Extra ANV N°	NMW & CO		
Extra ANV N°	NMW & CO		
Emergency (EMR) N°	NMW & CO		
EMR N°	NMW & CO		
EMR N°	NMW & CO		
EMR N°	NMW & CO		
Delivery	NMW & CO		
Ultrasound: Incl.	Ultrasonographer		
Ultrasound: Extra GA and UtA	Ultrasonographer		
Ultrasound: GA 20	Ultrasonographer		
Ultrasound: GA 26	Ultrasonographer		
Ultrasound: GA 32	Ultrasonographer		
Ultrasound: GA 37	Ultrasonographer		
Extra ultrasound	Ultrasonographer		
Extra ultrasound	Ultrasonographer		
Extra ultrasound	Ultrasonographer		
Only ultrasound, additional	Ultrasonographer		
Only ultrasound, additional	Ultrasonographer		

Mother's Pregnancy Form 1, Cohort Study PREG

1. **Antenatal Clinic** location (where form is being filled):

☐ Korogwe District Hospital ☐ Kerenge Dispensary
☐ Ngombezi Dispensary ☐ Lwengera Dispensary
☐ Majengo Dispensary ☐ Segera Dispensary
☐ Hale Dispensary ☐ Makuyuni Dispensary
☐ Chekelei Dispensary ☐ Other

1.1.1. If other, specify:____
1.2. **Name of study worker** filling the form:____
1.3. Date of filling CRF(*when filling of CRF is started)*: (dd/mm/yyyy) _ _ / _ _ / _ _ _ _
1.4. **Mother's surname**: ____
1.5. **Mother's first and second name**: ____
1.6. Changed name since inclusion in pre-pregn. part ☐ yes ☐ no
1.6.1. If yes specify name change:____

1.7. **Positive urine pregnancy test** ☐ yes ☐ no
1.8. UPT done by/during ☐ 3rd Monthly/Self-reported ☐ Governm. staff at satellite dispensary
1.8.1. If governmental, specify which satellite dispensary:_______________________________

1.9. **Number of menstrual cycles since last visit** (screening/inclusion/3M) _ _ ☐ Don't know
1.10. Number of pregnancies with miscarriage since screening/inclusion _ _ ☐ Don't know
1.10.1. If miscarriage, diagnosed by medical staff ☐ yes ☐ no
1.10.2. If miscarriage, give details: ____
____

CURRENT PREGNANCY DETAILS (on the day of filling the CRF (date=1.3))
1.10.3. **Hemoglobin level** at today's visit (Haemocue) _ _,_ g/dl
1.10.4. **Intrauterine pregnancy confirmed on ultrasound**
☐ yes ☐ pregnancy not visible ☐ Extrauterine
☐ not done ☐ GA not possible due to lie of foetus
1.10.4.1. If YES, gestational age by ultrasound: _ _ weeks _ days
1.10.4.2. If US not done, state reason:____
1.10.5. **Symphysis-fundal** height _ _ cm ☐ not palpable
1.10.6. **Date of last menstrual period** (LMP): (dd/mm/yyyy) _ _ / _ _ / _ _ _ _ ☐ Unknown
1.10.6.1. Gestational age by last menstrual period (LMP): _ _ weeks _ days
1.10.7. **Is this the first ANC visit** ☐ yes ☐ no
1.10.7.1. If not, how many previous visits (excluding the current visit): _ _

ELIGIBILITY FOR PREGNANCY PART in COHORT STUDY

1.10.8.	Screened/Included for PONA study I/cohort	☐ **yes** ☐ no	Exclusion if no
1.10.9.	Previously included in PONA pregnancy part	☐ yes ☐ **no**	Exclusion if yes
1.10.10.	Foetus visible, but non-viable pregnancy (miscarriage)	☐ yes ☐ **no**	Exclusion if yes
1.10.11.	Agrees to have venous blood drawn	☐ **yes** ☐ no	Exclusion if no
1.10.12.	Delivery planned at Korogwe District Hospital	☐ **yes** ☐ no	Exclusion if no
1.10.13.	Consents to participate in pregnancy part	☐ **yes** ☐ no	Exclusion if no

1.11. ☐ **INCLUSION** ☐ **REFUSAL** ☐ **EXCLUSION**
1.11.1. **In case of refusal or exclusion**, state reason: ______________________________
1.11.1.1. If excluded/refusal accept to have form 2 filled ☐ yes ☐ no
1.11.1.2. If yes, signed ICF cohort or ICF pregnancy ☐ yes ☐ no
1.11.2. **If ultrasound with precise GA not done**, state date of new ultrasound_ _ / _ _ / _ _ _ _
1.11.2.1. If booked for ultrasound, specify where: ______________________________

If UPT pos on <u>first</u> 3rd Monthly, and form 2 for pre-preg is not filled yet ***remember to fill form 12***

DATA ENTRY:
1st entry done by:_____________________________Signature:______________ date:_ _/_ _/_ _ _ _
2nd entry done by:_____________________________Signature:______________ date:_ _/_ _/_ _ _ _

2. Mother's Inclusion Form 2, Cohort Study PREG

2.1. Name of study worker filling the form: ____
2.2. Date of filling CRF: (dd/mm/yyyy) _ _ / _ _ / _ _ _ _

MATERNAL DEMOGRAPHIC DATA

2.3. **Home address** changed since inclusion pre-pregnancy ☐ yes ☐ no

If yes fill in 2.31 – 2.3.10

2.3.1. Street name:____
2.3.2. Street chairman:__ ____
2.3.3. Village: ____
2.3.4. Subvillage:____
2.3.5. Subvillage chair:____
2.3.6. District/ward: ____
2.3.7. Ten Cell leader:____
2.3.8. Name of Husband/partner if living together:__________________________________
2.3.9. Head of household:____
2.3.9.1. House number______________________
2.3.9.2. Known as Mama ____________________
2.3.10. Phone number (woman or partner)____

2.4. **Current residence**:
2.4.1. **Type of roof** on the house: ☐ Bati ☐ Tin (madebe) ☐Thatch ☐ Mixed Thatch/Tin ☐ Other
2.4.1.1. if other, specify: ____
2.4.2. **How many people** usually sleep in your residence _ _
2.4.3. **Who owns the house** you live in ☐ self/spouse – built ☐ inherited ☐ rental ☐ others
2.4.3.1. if others, specify:____
2.4.4. Type of **home toilet**: ☐ flush ☐ pit latrine(choo cha shimo) ☐ no toilet
2.4.5. Source of **water** ☐ tap(bombani) ☐ well (kisima (cha mdundi))) ☐ river/stream (mto) ☐ gutter water in tank (maji ya paa) ☐ pond/pool (bwawa) ☐ other
2.4.5.1. if other, specify: ____
2.4.5.2. if tap, well, or gutter water ☐ Private ☐ Public

2.5. **Current occupation** ☐ Professional ☐ Business ☐ Service ☐ Farmer ☐ Housewife/ working at home ☐ Other
2.5.1. If other, specify: ____
2.5.2. **Do you use chemicals** for pest/diseases <u>in your work</u> ☐ yes ☐ no
2.5.2.1. If yes, specify:____

2.6. **Marital status** ☐ Married ☐ Divorced ☐ Separated ☐ Never married ☐ Widow ☐ Refuse answer
2.6.1. If divorced/separated/widow/never married, do you currently have a partner ☐ yes, cohabiting ☐ yes, but not cohabiting ☐ no

2.7. **Who will take care of you and the newborn** ☐ Myself ☐ Parents ☐ Me and my husband/partner ☐ Others
2.7.1. If others, specify: ____

PATERNAL DEMOGRAPHIC DATA

2.8. Agree to answer questions about the father of the coming newborn ☐ yes ☐ no
2.9. Same person as the partner at during pre-pregn. part ☐ yes ☐ no

If yes go to question 2.15

2.10. **Name** of father of the coming newborn:_________________________________
2.11. **Age** of father of the coming newborn _ _ years ☐ unknown
2.12. **Ethnic group** ☐ Sambaa ☐ Zigua ☐ Para ☐ Bondei ☐ Other ☐ unknown
2.12.1. if other, specify: ____

2.13. **Education** ☐ none ☐ primary school partially completed
☐ primary school finished ☐ ≥secondary school ☐ unknown
2.13.1. Does he knows how to read and write ☐ yes ☐ no ☐ unknown
2.14. **Religion** ☐ Islamic ☐Catholic ☐ Lutheran
☐ Angikana ☐ Hindi ☐Other
2.14.1. If other, specify:____
2.15. **Current occupation** ☐ Professional ☐ Business ☐ Service
☐ Farmer ☐ Other ☐ unknown
2.15.1. If other, specify:____

NUTRITIONAL AND PHYSICAL HABITS
2.16. Has the "IPAQ questionnaire" been filled ☐ yes ☐ no
2.17. Has the "24hours Recall questionnaire" been filled ☐ yes ☐ no

MEDICAL HISTORY
Anaemia (upungufu wa damu)
2.18. **Has chronic anaemia now** (diagnosed by medical staff) ☐ yes, known irreversible cause
☐ yes, lasting>6months
☐ no
☐ don't know/not tested
2.18.1. If yes, cause known ☐ yes ☐ no
2.18.2. If yes, specify (e.g. sickle cell, severe malnutrition)____________________________
2.19. **Temporary anaemia diagnosed** within the last 4 months ☐ yes ☐ no
2.19.1. Nb. of times the last 4 month _ _
2.19.2. If yes, state first day of the last episode (dd/mm/yyyy) _ _/_ _/_ _ _ _
2.19.2.1. Last episode diagnosed by PONA ☐ yes ☐ no
2.19.2.2. Hemoglobin measurement done ☐ yes ☐ no
2.19.2.3. If yes, state the value (g/dL) _ _, _ ☐ unknown
2.20. **Received medicine for anaemia by** PONA last 4 months ☐ yes ☐ no
2.20.1. If yes, specify (put >1 if needed): ☐ Iron ☐ Folic ☐ B12
☐ Anti-helminths ☐ Hemovit
☐ Other
2.20.1.1. If other, specify: ____
2.20.2. State when treatment was started _ _ /_ _ /_ _ _ _
2.20.3. No. of tablets/day _ _ ☐ unknown
2.20.4. Dose per tablet _ _ _ _ ☐ unknown
2.20.5. State how long treatment was received: _ _ months _ _ weeks _ _ days ☐ unknown
2.20.6. Additional notes:____
2.21. **Received medicine for anemia not** by PONA last 4 months
☐ yes ☐ no
2.21.1. If yes, specify(put >1 if needed): ☐ Iron ☐ Folic ☐ B12
☐ Anti-helminths ☐ Hemovit
☐ Other
2.21.1.1. If other, specify____
2.21.2. State when treatment was started _ _ /_ _ /_ _ _ _
2.21.3. No. of tablets/day _ _ ☐ unknown
2.21.4. Dose per tablet _ _ _ _ ☐ unknown
2.21.5. State how long treatment was received: _ _ months _ _ weeks _ _ days ☐ unknown
2.21.6. Additional notes:____

Other chronic diseases
2.22. Since inclusion into pre-pregn. part diagnosed with a chronic illness by medical personnel (ask specifically about: diabetes type I or II (kisukari), kidney (figo), heart (moyo), thyroid (goiter) or lung (mapafu) disease, cancer (saratani), hypertension (shinikizo la damu), epilepsy (kifafa), lymphatic filariasis (matende), gastric ulcer (vidonda vya tumbo), autoimmune disorders (e.g. rheumatoid arthritis (rheumotism), chronic diarrhea (kuharisha kwa muda mrefu))
☐ yes ☐ no
2.22.1. if yes, specify____

2.23. **Gynaecological disorders**

2.23.1. Since inclusion into pre-pregn. part diagnosed with a pelvic inflammatory disease by medical personnel ☐ yes ☐ no

2.23.2. Since inclusion into pre-pregn. part diagnosed with any gynecological disorder by medical personnel (e.g. endometriosis, fibroma, cysts on the ovary, septum in uterus) ☐ yes ☐ no

2.23.2.1. If yes, specify: ____

2.23.3. Since inclusion into pre-pregn. part had abdominal surgery performed (e.g. surgery for appendicitis (kidole tumbo), gall bladder stone (mawe kwenye kibofu cha nyongo), uterus fibroma, ovary cysts) ☐ yes ☐ no

2.23.3.1. If yes, specify: ____

2.24. **HIV status** according to the woman:
☐ Unwilling to respond
☐ Positive
☐ Negative, will be re-testing today
☐ Negative, do not want re-testing
☐ Do not know, will be tested today
☐ Do not know, do not want testing

2.24.1. If positive, attending a CTC ☐ yes ☐ no

2.24.1.1. If yes, where:____

Malaria

2.25. **Does she have a bednet yes/no** ☐ yes ☐ no

2.25.1. If yes, did she use it last night? ☐ yes ☐ no

2.25.2. Impregnated bednet ☐ yes ☐ no ☐ unknown

2.25.3. Obtained from national program ☐ yes ☐ no ☐ unknown

2.26. **Nb. of malaria attacks** since the beginning of the current pregnancy?
☐ 0 ☐ 1 ☐ 2 ☐ 3 ☐ other

2.26.1. If other number, specify: ___

2.26.2. Date of the 1st malaria attack: (dd/mm/yyyy) _ _ / _ _ / _ _ _ _

2.26.3. Date of the 2nd malaria attack: (dd/mm/yyyy) _ _ / _ _ / _ _ _ _

2.26.4. Date of the 3rd malaria attack: (dd/mm/yyyy) _ _ / _ _ / _ _ _ _

2.27. **1st malaria attack:**

2.27.1. Was malaria confirmed with a blood test ☐ yes ☐ no ☐ unspecified

2.27.2. What treatment did you take? ☐ Quinine ☐ SP/ Metakelfin ☐ Chloroquine ☐ Herbal remedy ☐ None ☐ unspecified ☐ other ☐ ALU

2.27.2.1. If other, details: ____

2.28. **2nd malaria attack:**

2.28.1. Was malaria confirmed with a blood test ☐ yes ☐ no ☐ unspecified

2.28.2. What treatment did you take? ☐ Quinine ☐ SP/ Metakelfin ☐ Chloroquine ☐ Herbal remedy ☐ None ☐ unspecified ☐ other ☐ ALU

2.28.2.1. If other, details: ____

2.29. **3rd malaria attack:**

2.29.1. Was malaria confirmed with a blood test ☐ yes ☐ no ☐ unspecified

2.29.2. What treatment did you take? ☐ Quinine ☐ SP/ Metakelfin ☐ Chloroquine ☐ Herbal remedy ☐ None ☐ unspecified ☐ other ☐ ALU

2.29.2.1. If other, details: ____

2.30. **IPT-SP** taken during the current pregnancy ☐ no ☐ 1 dose ☐ 2 doses ☐ 3 doses

2.31. **Other malaria chemoprophylaxis** during current pregnancy? ☐ yes ☐ no

2.31.1. If yes, what treatment was taken? ☐ Chloroquine ☐ Herbal ☐ unspecified ☐ other

2.31.1.1. If other, details : ____

Current usage of medicine

2.32. Currently taking medication? ☐ yes ☐ no

2.32.1. If yes, give details (put >1 if needed): ☐ antibiotics ☐ antimalarial ☐ iron ☐ antiretrovirals ☐ folic acid ☐ B12 ☐ antihelminths ☐ traditional ☐ Hemovit ☐ antihypertensive ☐ painkillers ☐ other

2.32.1.1. If other or painkillers,specify: ____________________

2.32.1.2. If, traditional specify: ____________________

2.32.1.3. Dosage of these drugs: ____________________

____

____

____

____

Substance abuse:

2.33. Smoker ☐ yes ☐ no

2.33.1. If yes, number of cigarettes per _ _ month _ _ week _ _ day

2.34. Usage of alcohol during this pregnancy ☐ yes ☐ no

2.34.1. If yes, how many items per _ _ month _ _ week _ _ day

2.35. Usage of caffeine beverages ☐ yes ☐ no

2.35.1. If Coke, how many items (bottles) per _ _ month _ _ week _ _ day

2.35.2. If coffee, how many items (cups) per _ _ month _ _ week _ _ day

2.35.3. If tea, how many items (cups) per _ _ month _ _ week _ _ day

MEDICAL EXAMINATION *(all * should be filled by CO/AMO/MD)*

2.36. Woman's weight (kg) _ _ _ , _

2.37. Waist circumference (cm) (at top of iliac crest) _ _ _ , _ ☐ not done

2.38. Hip circumference (cm) (widest portion of the buttocks) _ _ _ , _

2.39. MUAC (cm) _ _ , _

2.40. Skinfold thickness of triceps (mm) _ _ , _

2.41. Blood pressure (BP) (mmHg)

2.41.1. 1st BP, left arm _ _ _ / _ _ _

2.41.2. 1st BP, right arm _ _ _ / _ _ _

2.41.3. Difference in left and right arm (max 20 syst.; 10 diast.) _ _ / _ _

2.41.4. Reference arm (the arm with the highest BP) ☐ Right ☐ Left

2.41.5. Reference arm (the arm with the highest BP), 2nd BP _ _ _ / _ _ _

2.41.6. Mean BP (1st and 2nd BP for reference arm) _ _ _ / _ _ _

2.41.7. If, mean BP >140/90 repeat after 4 hours, BP at repeat _ _ _ / _ _ _ ☐ not done

2.41.8. After how many hours was repeat BP performed _ _ ☐ not done

2.41.9. Pulse (use last BP measurement) _ _ _

2.42. Axillary temperature (°C) _ _ , _

2.43. * Headache ☐ yes ☐ no

2.44. * Visual disturbances ☐ yes ☐ no

2.44.1. If yes, specify: ____________________

2.45. * Dizziness ☐ yes ☐ no

2.46. * Denuded/glossy tongue (glossitis) ☐ yes ☐ no

2.47. * Commisura of the lips (cheilosis, fissure) ☐ yes ☐ no

2.48. * Vitiligo ☐ yes ☐ no

2.49. * Pallor (conjunctivae or palms of hands) ☐ yes ☐ no

2.50. * Deformities of the nails (flattening/koilonychia) ☐ yes ☐ no

2.51. * Pitting oedema (swelling of lower/upper limps or/and face) ☐ yes ☐ no

2.52. * Heart palpitations ☐ yes ☐ no

2.53. * Angina pectoris ☐ yes ☐ no

2.54. * Cardiac murmurs ☐ yes ☐ no

2.55. * Dyspnea ☐ yes ☐ no

2.56. * Pulmonary stethoscopic signs of abnormalities ☐ yes ☐ no

2.56.1. If yes, specify:____________________

2.57. * Nausea and/or vomiting ☐ yes ☐ no

2.58. * Hematemesis ☐ yes ☐ no

2.59. * Severe epigastric pain ☐ yes ☐ no
2.60. * Enlarged spleen ☐ yes ☐ no
2.61. * Melena ☐ yes ☐ no
2.62. * Other symptoms ☐ yes ☐ no
2.62.1. If yes, specify:____

SAMPLES COLLECTED

2.63. **Malaria** RDT ☐ negative ☐ PF ☐ PAN ☐ PF+PAN ☐ not done
2.64. **HIV** RDT, Determine ☐ negative ☐ positive ☐ not done
2.65. **Venous blood** draw ☐ done ☐ not done
2.65.1. if not done, why : ☐ refusal ☐ failure ☐ forgot
2.66. CPDA Tube : ☐ done ☐ not done ☐ not applicable
2.67. EDTA Tube (6ml + 2ml): ☐ done ☐ not done
2.68. Plain Tube (6ml + eppendorf): ☐ done ☐ not done
2.69. **Blood group**: ☐ done ☐ to be done
2.69.1. Result: ☐ A+ ☐ A- ☐ B+ ☐ B- ☐ AB+ ☐ AB- ☐ O+ ☐ O-
2.70. **Urine dipstick**
2.70.1. Albumin in the urine ☐ 0+ ☐ 1+ ☐ 2+ ☐ 3+ ☐ not done
2.70.2. Sugar in the urine ☐ 0+ ☐ 1+ ☐ 2+ ☐ 3+ ☐ 4+ ☐ 5+ ☐ not done
2.70.3. Leucocytes in the urine ☐ 0+ ☐ 1+ ☐ 2+ ☐ 3+ ☐ not done
2.70.4. Blood in urine ☐ 0+ ☐ 1+ ☐ 2+ ☐ 3+ ☐ not done
2.70.5. Ketones ☐ 0+ ☐ 1+ ☐ 2+ ☐ 3+ ☐ not done
2.70.6. Nitrite ☐ 0+ ☐ 1+ ☐ 2+ ☐ not done

CONCLUSION ON TODAY'S EXAMINATION

2.71. **Ailment/disease** diagnosed today ☐ yes ☐ no
2.71.1. If yes, specify (≥1 "x") ☐ Anaemia ☐ Malaria ☐ (Suspected) hypertension
☐ Urinary tract infection ☐ Syphilis ☐ HIV
☐ Upper respiratory tract infect. ☐ Diabetes
☐ Reproductive tract infection ☐ Other
2.71.1.1. If other, specify:____
2.72. **Treatment** prescribed today ☐ yes ☐ no
2.72.1. If yes, specify (≥1 "x") ☐ Coartem/ALU ☐ Quinine ☐ anti-helminth
☐ Iron ☐ Folic acid ☐ B12 ☐ Hemovit
☐ Antibiotics ☐ Anti-HT ☐ Painkillers ☐ Other
2.72.1.1. If painkillers or other, specify: ______________________________________
2.72.2. Specify name, dosage and duration of treatment: ____________________________
____
2.73. **Tetanus toxoid immunization** (TT) dose received today ☐ yes ☐ no
2.73.1. **If no, state reason:** ____
2.73.2. Nb of TT doses received until todays visit (excl. todays dose) _ _ ☐ Unknown
2.73.3. Last TT dose received when _ _ / _ _ / _ _ _ _ ☐ Don't know
2.74. **Name or address** changed since pre-pregn. part ☐ yes ☐ no
2.74.1. If yes, new ID card made ☐ yes ☐ no
2.75. Additional notes:____
____
____
____

Next visit booked on: _ _ / _ _ / _ _ _ _, specify visit type: ____________

Questions marked * filled in by (MD, AMO or clinical officer): ______________________________

Signature:_________________

DATA ENTRY:

1st entry done by:____Signature:________________ date:_ _/_ _/_ _ _ _

2nd entry done by:____Signature:________________ date:_ _/_ _/_ _ _ _

Identification No. : F N _ _ _ _ _ _ _ _	Version 9, 1july2016

3. Antenatal (ANV)/Emergency (EMR) visit follow-up form PREG

3.1. Antenatal Clinic location: ☐ Korogwe District hospital ☐ Other

3.1.1. If other, specify:____

3.2. Project staff name: ____

3.3. Date of filling CRF ((dd/mm/yyyy): _ _ / _ _ / _ _ _ _

3.4. Gestational age (by UL): _ _ Weeks _ _ days

3.5. **Type of visit** ☐ ANV ☐ Extra ANV ☐ EMR ☐ Extra US GA 11-14 ☐ only UL

3.5.1. **Number of this type** of visit (incl. today) _ _

If only UL (e.g. AFI control only due to overdue) the rest of the CRF should not be filled. If extra ANV or EMR visit, UL form is only filled and blood sample only taken after individual case evaluation by the clinician in charge. For all questions stating "since your last PONA visits", "last visit" refer to the last visit where form 3 (or 2 or 14) was filled – excluding visits where only UL is performed – and disregarding if blood sample was taken.

3.5.2. **If extra ANV**, specify reason for control: ☐ Anaemia ☐ Diabetes ☐ Malaria ☐ Preeclampsia ☐ Hypertension ☐ IUGR suspected ☐ Other

3.5.2.1. If other, specify: ____

3.5.3. **If EMR**, specify reason:____

____

MEDICAL EXAMINATION *(all * should be filled by CO/AMO/MD)*

3.6.	Woman's weight (kg)	_ _ _ , _	☐ Not done
3.7.	Hip circumference (cm) (widest portion of the buttocks)	_ _ _ , _	☐ Not done
3.8.	MUAC (cm)	_ _ , _	☐ Not done
3.9.	Skinfold thickness of triceps (mm)	_ _ , _	☐ Not done
3.10.	Blood pressure (BP) (mmHg)		
3.10.1.	1st BP (reference arm, see form 2)	_ _ _ / _ _ _	
3.10.2.	2nd BP (reference arm, see form 2)	_ _ _ / _ _ _	
3.10.3.	Mean BP (1st and 2nd BP for reference arm)	_ _ _ / _ _ _	
3.10.4.	If, mean BP ≥140/90 repeat after 4 hours, BP at repeat	_ _ _ / _ _ _	☐ Not done
3.10.5.	After how many hours was repeat BP performed	_ _	☐ Not done
3.10.6.	Pulse (use last BP measurement)	_ _ _	
3.11.	Axillary temperature (°C)	_ _ , _	
3.12.	* Headache	☐ yes	☐ no
3.13.	* Visual disturbances	☐ yes	☐ no
3.13.1.	If yes, specify: ________________________________		
3.14.	* Dizziness	☐ yes	☐ no
3.15.	* Denuded/glossy tongue (glossitis)	☐ yes	☐ no
3.16.	* Commisura of the lips (cheilosis, fissure)	☐ yes	☐ no
3.17.	* Vitiligo	☐ yes	☐ no
3.18.	* Pallor (conjunctivae or palms of hands)	☐ yes	☐ no
3.19.	* Deformities of the nails (flattening/koilonychia)	☐ yes	☐ no
3.20.	* Pitting oedema (swelling of lower/upper limps or/and face)	☐ yes	☐ no
3.21.	* Heart palpitations	☐ yes	☐ no
3.22.	* Angina pectoris	☐ yes	☐ no
3.23.	* Cardiac murmurs	☐ yes	☐ no
3.24.	* Dyspnea	☐ yes	☐ no
3.25.	* Pulmonary stethoscopic signs of abnormalities	☐ yes	☐ no
3.25.1.	If yes, specify:________________________________		
3.26.	* Nausea and/or vomiting	☐ yes	☐ no
3.27.	* Hematemesis	☐ yes	☐ no
3.28.	* Severe epigastric pain	☐ yes	☐ no
3.29.	* Enlarged spleen	☐ yes	☐ no
3.30.	* Melena	☐ yes	☐ no
3.31.	* Other symptoms	☐ yes	☐ no
3.31.1.	If yes, specify:________________________________		

3.32. **Symphysis-fundus length** (cm) _ _ , _

If EMR visit or signs of severe illness answer the following questions as well:

3.33. * Feverishness in the last 48hrs ☐ yes ☐ no
3.34. * Convulsions ☐ yes ☐ no
3.35. * Shivering ☐ yes ☐ no
3.36. * Comatose or sub-comatose ☐ yes ☐ no
3.37. * Abdominal pain ☐ yes ☐ no
3.38. * Bleeding ☐ yes ☐ no
3.39. * Other clinical signs:____

____

3.40. * Pregnancy at risk ☐ yes ☐ no

NUTRITIONAL AND PHYSICAL HABITS

3.41. Has the "IPAQ questionnaire" been filled (only ANV) ☐ yes ☐ no
3.42. Has the "24hours Recall questionnaire" been filled (only ANV) ☐ yes ☐ no

MEDICAL EMERGENCIES AND TREATMENT since last PONA visit

3.43. **Since your last PONA visit** did you seek medical help elsewhere (not PONA staff) because you **suspected anaemia** (upungufu wa damu)? ☐ yes ☐ no
3.43.1. If yes, when? _ _ / _ _ / _ _ _ _
3.43.2. Was it confirmed with Hb measurement ☐ yes ☐ no ☐ unspecified
3.43.2.1. If yes, specify Hb (g/dL) _ _ , _ ☐ unspecified
3.43.3. Medication (put >1 "x" if needed) ☐ iron ☐ Folic ☐ B12 ☐ antihelminths ☐ Hemovit ☐ unspecified ☐ other ☐ none
3.43.3.1. If other, specify:____
3.43.4. No. of tablets/day _ _ ☐ unknown
3.43.5. Dose per tablet _ _ _ _ ☐ unknown
3.43.6. State how long treatment was received: _ _ months _ _ weeks _ _ days ☐ unknown
3.44. **Since your last PONA visit** did you seek medical help (not PONA staff) because you **suspected malaria**? ☐ yes ☐ no
3.44.1. If yes, when? _ _ / _ _ / _ _ _ _
3.44.2. Did you have fever? ☐ yes ☐ no ☐ unspecified
3.44.3. Was malaria confirmed with a blood test ☐ yes ☐ no ☐ unspecified
3.44.4. Medication (put >1 "x" if needed) ☐ SP/Metakelfin ☐ Coartem/ALU ☐ quinine ☐ unspecified ☐ other ☐ none
3.44.4.1. If other, specify: ____
3.45. **Since your last visit have you acquired a bednet?** ☐ yes ☐ no
3.46. **Did you use a bednet last night?** ☐ yes ☐ no
3.46.1. Supplied by the national programme ☐ yes ☐ no ☐ unknown
3.46.2. Is it an insecticide-impregnated net? ☐ yes ☐ no ☐ unknown

3.47. **Since your last PONA visit, have you visited another health center/pharmacy** for other reasons than anaemia and malaria: ☐ yes ☐ no
3.47.1. Date (dd/mm/yyyy): _ _ / _ _ / _ _ _ _
3.47.2. Why? ____
3.47.3. Diagnosed with: ____
3.47.4. Medication prescribed:___________________________________
3.48. **Since your last visit have you attended a CTC** ☐ yes ☐ no ☐ not relevant, HIV negative
3.48.1. If yes, which one:____

MEDICINE USAGE since last PONA visit

Since your last PONA visit (any visit Form 2 or 3 was filled), have you taken any of the following (prescribed by the project, other health facility and/or pharmacy):

3.49. SP for IPTp ☐ yes ☐ no ☐ unspecified
3.50. SP other than for IPT ☐ yes ☐ no ☐ unspecified
3.51. Other anti-malaria drugs ☐ yes ☐ no ☐ unspecified
3.51.1. If yes, specify: ____
3.52. Folic acid ☐ yes ☐ no ☐ unspecified
3.52.1. No. of tablets/day _ _ ☐ unknown
3.52.2. Dose per tablet _ _ _ _ ☐ unknown
3.52.3. State how long treatment was received: _ _ weeks _ _days ☐ unknown
3.53. Iron (regular doses) ☐ yes ☐ no ☐ unspecified
3.53.1. No. of tablets/day _ _ ☐ unknown
3.53.2. Dose per tablet _ _ _ _ ☐ unknown
3.53.3. State how long treatment was received: _ _ weeks _ _days ☐ unknown
3.54. Hemovit ☐ yes ☐ no ☐ unspecified
3.54.1. mL per day _ _ _ ☐ unknown
3.54.2. State how long treatment was received: _ _ weeks _ _days ☐ unknown
3.55. B12 ☐ yes ☐ no ☐ unspecified
3.55.1. No. of tablets/day _ _ ☐ unknown
3.55.2. Dose per tablet _ _ _ _ ☐ unknown
3.55.3. State how long treatment was received: _ _ weeks _ _days ☐ unknown
3.56. Other vitamins (not iron, folic and B12): ☐ yes ☐ no ☐ unspecified
3.56.1. No. of tablets/day _ _ ☐ unknown
3.56.2. Dose per tablet _ _ _ _ ☐ unknown
3.56.3. State how long treatment was received: _ _ weeks _ _days ☐ unknown
3.57. Anti-retroviral ☐ yes ☐ no ☐ unspecified
3.57.1. Type of medicine and dose:__________________________________
3.58. Anti-helminth: ☐ yes ☐ no ☐ unspecified
3.58.1. No. of tablets/day _ _ ☐ unknown
3.58.2. Dose per tablet _ _ _ _ ☐ unknown
3.58.3. State how long treatment was received: _ _ weeks _ _days ☐ unknown
3.59. Anti hypertensives : ☐ yes ☐ no ☐ unspecified
3.59.1. Type of medicine and dose:__________________________________
3.59.2. No. of tablets/day _ _
3.59.3. State how long treatment was received: _ _ weeks _ _days ☐ unknown
3.60. Antibiotics: ☐ yes ☐ no ☐ unspecified
3.60.1. No. of tablets/day _ _ ☐ unknown
3.60.2. Dose per tablet _ _ _ _ ☐ unknown
3.60.3. State how long treatment was received: _ _ weeks _ _days ☐ unknown
3.61. Painkillers (e.g. paracetamol) ☐ yes ☐ no ☐ unspecified
3.61.1. If painkillers, specify type:___________________________________
3.62. Other medicine ☐ yes ☐ no ☐ unspecified
3.62.1. If other, specify:_______________________________________
3.63. Any medication taken within 48hours prior to this visit ☐ yes ☐ no
3.63.1. If yes, which (traditional remedy, drugs): ___________________________
____

SAMPLES COLLECTED

3.64. **Malaria** RDT ☐ negative ☐ PF ☐ PAN ☐ PF+PAN ☐ not done
3.65. **HIV** RDT, Determine ☐ negative ☐ positive ☐ not done
3.66. **Venous blood** draw ☐ done ☐ not done
3.66.1. if not done, why : ☐ refusal ☐ failure ☐ forgot
3.67. CPDA Tube: ☐ done ☐ not done ☐ not applicable
3.68. EDTA Tube (6ml): ☐ done ☐ not done ☐ not applicable
3.69. EDTA Tube (2ml): ☐ done ☐ not done ☐ not applicable
3.70. Plain Tube (6ml): ☐ done ☐ not done ☐ not applicable
3.71. Plain Tube (eppendorf): ☐ done ☐ not done ☐ not applicable

3.72. **OGTT**	☐ done	☐ not done		☐ not applicable
3.72.1. If done; BS at time 0	_ _ , _	☐ fingerprick		☐ venous
3.72.2. If done; BS at time 1hours	_ _ , _	☐ fingerprick		☐ venous
3.72.3. If done; BS at time 2hours	_ _ , _	☐ fingerprick		☐ venous
3.73. HbA1C			_ _ , _	☐ not done

3.74. **Urine dipstick**

3.74.1. Albumin in the urine	☐ 0+	☐ 1+	☐ 2+	☐ 3+			☐ not done
3.74.2. Sugar in the urine	☐ 0+	☐ 1+	☐ 2+	☐ 3+	☐ 4+	☐5+	☐ not done
3.74.3. Leucocytes in the urine	☐ 0+	☐ 1+	☐ 2+	☐ 3+			☐ not done
3.74.4. Blood in urine	☐ 0+	☐ 1+	☐ 2+	☐ 3+			☐ not done
3.74.5. Ketones	☐ 0+	☐ 1+	☐ 2+	☐ 3+			☐ not done
3.74.6. Nitrite	☐ 0+	☐ 1+	☐ 2+				☐ not done

CONCLUSION ON TODAYS EXAMINATION

3.75. **IPTp** taken during this ANC visit? ☐ no ☐ 1st ☐ 2nd ☐ 3rd ☐ 4th ☐ 5th Dose
3.75.1. If yes, prescribed by PONA ☐ yes ☐ no
3.75.2. If not taken IPTp, why: ☐ Cotrimoxazole usage ☐ allergy
☐ too early or late in pregnancy
☐ completed IPTp regime ☐ refuse
☐ <1 month since last dose ☐ Other
3.75.2.1. If other, specify:____
3.76. Nb of IPTp doses received until todays visit (excl. todays dose) _ _ ☐ unknown
3.77. **Tetanus toxoid** immunization (TT) dose received ☐ yes ☐ no
3.77.1. If no, state reason: ____
3.77.2. Nb of TT doses received until todays visit (excl. todays dose) _ _ ☐ unknown
3.77.3. Last TT dose received when _ _ / _ _ / _ _ _ _ ☐ Don't know
3.78. **Ailment/disease** diagnosed today ☐ yes ☐ no
3.78.1. If yes, specify (≥1 "x") ☐ Anaemia ☐ Malaria ☐ Hypertension
☐ Urinary tract infection ☐ Preeclampsia
☐ Upper respiratory tract infect. ☐ Syphilis
☐ Reproductive tract infection ☐ Diabetes
☐ HIV ☐ Other
3.78.1.1. If other, specify:____
3.79. **Treatment** prescribed today ☐ yes ☐ no
3.79.1. If yes, specify (≥1 "x") ☐ Coartem/ALU ☐ Quinine ☐ anti-helminth
☐ Iron ☐ Folic acid ☐ B12
☐ Hemovit ☐ Antibiotics ☐ Anti-HT
☐ Painkillers ☐ Other
3.79.1.1. If other or painkillers, specify: ____
3.80. Specify name, dosage and duration of treatment:
3.80.1. 1st ____
3.80.2. 2nd ____
3.80.3. 3rd ____
3.80.4. 4th ____
3.81. **Ultrasound** performed ☐ yes ☐ no
3.82. Notes____
____
____

Next visit booked on: _ _ / _ _ / _ _ _ _, specify visit type: ____________

Questions marked * filled in by (MD, AMO or clinical officer): ________________________________
Signature:__________________

DATA ENTRY:
1st entry done by:________________________Signature:__________________ date:_ _/_ _/_ _ _ _
2nd entry done by:________________________Signature:__________________ date:_ _/_ _/_ _ _ _

4. Delivery Form PREG (ALWAYS FILL IN)

4.1. **Maternity service location (on examination)** ☐Korogwe District hospital ☐ Home ☐ Other Dispensary ☐ Other

4.1.1. If other dispensary/other, specify:____

4.2. Date of filling CRF (dd/mm/yyyy) (**q. 4.10-4.75.4)** _ _ / _ _ / _ _ _ _

4.3. Time of filling CRF (**q. 4.10-4.75.4)** _ _ : _ _

4.4. Name of midwife/nurse/auxiliary worker: ____

4.5. **Place of delivery** ☐Korogwe District hospital ☐ Home ☐ Other Dispensary ☐ Other

4.5.1. If other dispensary/other, specify:____

4.6. **Mother referred to another facility** after delivery ☐ yes ☐ no

4.6.1. if yes, details: ☐ Korogwe District hospital ☐ KCMC ☐ Bombo referral Hospital ☐ Other

4.6.2. if referred, state reasons:____

4.7. **Date of delivery** (dd/mm/yyyy) _ _ / _ _ / _ _ _ _

4.8. **Time of delivery** __ : __ ☐ Unknown

4.9. **Gestational age** at delivery (calculated using US, form 8) _ _week _ _ days

In all questions stating “since your last PONA visits”, “last visit” refer to the last visit where form 3 (or 2 or 14) was filled – excluding visits where only UL is performed.

MEDICAL EXAMINATION *(all * should be filled by CO/AMO/MD)* ***(done until 2 weeks after delivery)***

4.10.	Woman's weight (kg)	_ _ _ , _	☐ Not done
4.11.	Hip circumference (cm) (widest portion of the buttocks)	_ _ _ , _	☐ Not done
4.12.	MUAC (cm)	_ _ , _	☐ Not done
4.13.	Skinfold thickness of triceps (mm)	_ _ , _	☐ Not done
4.14.	Blood pressure (BP) (mmHg)		
4.14.1.	1st BP (reference arm, see form 2)	_ _ _ / _ _ _	☐ Not done
4.14.2.	2nd BP (reference arm, see form 2)	_ _ _ / _ _ _	☐ Not done
4.14.3.	Mean BP (1st and 2nd BP for reference arm)	_ _ _ / _ _ _	☐ Not done
4.14.4.	If, mean BP >140/90 repeat after 4 hours, BP at repeat	_ _ _ / _ _ _	☐ Not done
4.14.5.	After how many hours was repeat BP performed	_ _	☐ Not done
4.14.6.	Pulse (use last BP measurement)	_ _ _	☐ Not done
4.15.	Axillary temperature (°C)	_ _ , _	☐ Not done
4.16.	* Headache	☐ yes	☐ no
4.17.	* Visual disturbances	☐ yes	☐ no
4.17.1.	If yes, specify: ________________________________		
4.18.	* Dizziness	☐ yes	☐ no
4.19.	* Denuded/glossy tongue (glossitis)	☐ yes	☐ no
4.20.	* Commisura of the lips (cheilosis, fissure)	☐ yes	☐ no
4.21.	* Vitiligo	☐ yes	☐ no
4.22.	* Pallor (conjunctivae or palms of hands)	☐ yes	☐ no
4.23.	* Deformities of the nails (flattening/koilonychia)	☐ yes	☐ no
4.24.	* Pitting oedema (swelling of lower/upper limps or/and face)	☐ yes	☐ no
4.25.	* Heart palpitations	☐ yes	☐ no
4.26.	* Angina pectoris	☐ yes	☐ no
4.27.	* Cardiac murmurs	☐ yes	☐ no
4.28.	* Dyspnea	☐ yes	☐ no
4.29.	* Pulmonary stethoscopic signs of abnormalities	☐ yes	☐ no
4.29.1.	If yes, specify:________________________________		
4.30.	* Nausea and/or vomiting	☐ yes	☐ no
4.31.	* Hematemesis	☐ yes	☐ no
4.32.	* Severe epigastric pain	☐ yes	☐ no
4.33.	* Enlarged spleen	☐ yes	☐ no
4.34.	* Melena	☐ yes	☐ no
4.35.	* Other symptoms	☐ yes	☐ no
4.35.1.	If yes, specify:________________________________		
4.36.	**Symphysis-fundus length** (cm) (if examined before delivery)	_ _	☐ Not done

NUTRITIONAL AND PHYSICAL HABITS

4.37. Has the "IPAQ questionnaire" been filled ☐ yes ☐ no
4.38. Has the "24hours Recall questionnaire" been filled ☐ yes ☐ no

MEDICAL EMERGENCIES AND TREATMENT since last PONA visit

4.39. **Since your last PONA visit** did you seek medical help because you **suspected anaemia (upungufu wa damu)?** ☐ yes ☐ no ☐ not asked
4.39.1. If yes, when? _ _ / _ _ / _ _ _ _
4.39.2. Was it confirmed with Hb measurement ☐ yes ☐ no ☐ unspecified
4.39.2.1. If yes, specify Hb _ _ , _ ☐ unspecified
4.39.3. Medication (put >1 "x" if needed) ☐ iron ☐ Folic ☐ B12 ☐ antihelminths ☐ Hemovit ☐ unspecified ☐ other ☐ none
4.39.3.1. If other, specify:____
4.39.4. No. of tablets/day _ _ ☐ unknown
4.39.5. Dose per tablet _ _ ☐ unknown
4.39.6. State how long treatment was received: _ _ weeks _ _days ☐ unknown
4.40. **Since your last PONA visit** did you seek medical help because you **suspected malaria**? ☐ yes ☐ no ☐ not asked
4.40.1. If yes, when? _ _ / _ _ / _ _ _ _
4.40.2. Did you have fever? ☐ yes ☐ no ☐ unspecified
4.40.3. Was malaria confirmed with a blood test ☐ yes ☐ no ☐ unspecified
4.40.4. Medication (put >1 "x" if needed) ☐ coartem/ALU ☐ SP/Metakelfin ☐ quinine ☐ unspecified ☐ other ☐ none
4.40.5. If other, specify: ____
4.41. **Since your last visit have you acquired a bednet?** ☐ yes ☐ no ☐ not asked
4.42. **Did you use a bednet last night?** ☐ yes ☐ no ☐ not asked
4.42.1. Supplied by the national programme ☐ yes ☐ no ☐ unknown
4.42.2. Is it an insecticide-impregnated net? ☐ yes ☐ no ☐ unknown

4.43. **Since your last PONA visit, have you visited another health center/pharmacy** for other reasons than anaemia and malaria: ☐ yes ☐ no ☐ not asked
4.43.1. Date (dd/mm/yyyy): _ _ / _ _ / _ _ _ _
4.43.2. Why? ____
4.43.3. Diagnosed with: ____
4.43.4. Medication prescribed:____

4.43.5. **Since you last PONA visit have you attended a CTC** ☐ yes ☐no ☐not asked ☐ not relevant, HIV negative
4.43.5.1. If yes, which one:____

MEDICINE USAGE since last PONA visit

Since your last PONA visit (Inclusion, ANV, extra ANV, EMR where Form 3 was filled), have you taken any of the following (prescribed by the project, other health facility and/or pharmacy):

4.44. SP for IPTp ☐ yes ☐ no ☐ unspecified
4.45. SP other than for IPTp ☐ yes ☐ no ☐ unspecified
4.46. Other anti-malaria drugs ☐ yes ☐ no ☐ unspecified
4.46.1. If yes, specify: ____
4.47. Folic acid ☐ yes ☐ no ☐ unspecified
4.47.1. No. of tablets/day _ _ ☐ unknown
4.47.2. Dose per tablet _ _ _ _ ☐ unknown
4.47.3. State how long treatment was received: _ _ weeks _ _days ☐ unknown
4.48. Iron (regular doses) ☐ yes ☐ no ☐ unspecified
4.48.1. No. of tablets/day _ _ ☐ unknown
4.48.2. Dose per tablet _ _ _ _ ☐ unknown
4.48.3. State how long treatment was received: _ _ weeks _ _days ☐ unknown

4.49.	Hemovit	☐ yes ☐ no	☐ unspecified
4.49.1.	mL per day	_ _ _	☐ unknown
4.49.2.	State how long treatment was received:	_ _ weeks _ _days	☐ unknown
4.50.	B12	☐ yes ☐ no	☐ unspecified
4.50.1.	No. of tablets/day	_ _	☐ unknown
4.50.2.	Dose per tablet	_ _ _ _	☐ unknown
4.50.3.	State how long treatment was received:	_ _ weeks _ _days	☐ unknown
4.51.	Other vitamins (not iron, folic and B12):	☐ yes ☐ no	☐ unspecified
4.51.1.	No. of tablets/day	_ _	☐ unknown
4.51.2.	Dose per tablet	_ _ _ _	☐ unknown
4.51.3.	State how long treatment was received:	_ _ weeks _ _days	☐ unknown
4.52.	Anti-retroviral	☐ yes ☐ no	☐ unspecified
4.52.1.	Type of medicine and dose:____		
4.53.	Anti-helminth:	☐ yes ☐ no	☐ unspecified
4.53.1.	No. of tablets/day	_ _	☐ unknown
4.53.2.	Dose per tablet	_ _ _ _	☐ unknown
4.53.3.	State how long treatment was received:	_ _ weeks _ _days	☐ unknown
4.54.	Anti hypertensives :	☐ yes ☐ no	☐ unspecified
4.54.1.	Type of medicine and dose:____		
4.54.2.	No. of tablets/day	_ _	
4.54.3.	State how long treatment was received:	_ _ weeks _ _days	☐ unknown
4.55.	Antibiotics:	☐ yes ☐ no	☐ unspecified
4.55.1.	No. of tablets/day	_ _	☐ unknown
4.55.2.	Dose per tablet	_ _ _ _	☐ unknown
4.55.3.	State how long treatment was received:	_ _ weeks _ _days	☐ unknown
4.56.	Painkillers (e.g. paracetamol)	☐ yes ☐ no	☐ unspecified
4.56.1.	If painkillers, specify type:____		
4.57.	Other medicine	☐ yes ☐ no	☐ unspecified
4.57.1.	If other, specify:____		
4.58.	Any medication taken within 48hours prior to this visit	☐ yes	☐ no ☐ unspecified
4.58.1.	If yes, which (traditional remedy, drugs): __________________________________ ____		

MEDICINE USE & VISITS DURING PREGNANCY (*ask the woman & control previous visit forms*)

4.59.	**Nb of IPTp doses** received during pregnancy in total?		_ _
4.59.1.	If not completed (at least 2 for all women, 3 for HIV positive not on cotrimoxazole), why:		
		☐ Cotrimoxazole usage	☐ allergy
		☐ missed ANV with IPTp	☐ refused
		☐ Other	
4.59.1.1.	If other, specify:____		
4.60.	**Tetanus toxoid immunization** (TT) dose received today	☐ yes	☐ no
4.60.1.	If no, state reason: ____		
4.60.2.	Nb of TT doses received until todays visit (excl. todays dose)	_ _	☐ UN
4.60.3.	Last TT dose received when	_ _ / _ _ / _ _ _ _	☐ Don't know
4.61.	**Total number of TT doses** received during this pregnancy	_ _	☐ UN
4.62.	**Completed all ANV** (incl, GA 20, GA 26, GA 32, GA 37)	☐ yes	☐ no

SAMPLE COLLECTION (collected until 2 weeks after delivery)

4.63.	**Date of sample collection** (dd/mm/yyyy)			_ _ / _ _ / _ _ _ _	☐ Not Done
4.64.	**Time of sample collection**			_ _ : _ _	
4.65.	Samples collected during	☐ latent phase<br>☐ after delivery		☐ active phase (>4cm dilated)	
4.66.	Malaria RDT ☐ negative	☐ PF	☐ PAN	☐ PF+PAN	☐ not done
4.67.	HIV RDT, Determine	☐ negative	☐ positive	☐ not done	
4.68.	**Venous blood** draw	☐ done	☐ not done		
4.68.1.	if not done, why :	☐ refusal	☐ failure	☐ forgot	
4.69.	CPDA Tube :	☐ done	☐ not done	☐ not applicable	
4.70.	EDTA Tube (6ml + 2ml):	☐ done	☐ not done		
4.71.	Plain Tube (6ml + eppendorf):	☐ done	☐ not done		

4.72.	**Urine dipstick**							
4.72.1.	Albumin in the urine	☐ 0+	☐ 1+	☐ 2+	☐ 3+			☐ not done
4.72.2.	Sugar in the urine	☐ 0+	☐ 1+	☐ 2+	☐ 3+	☐ 4+	☐5+	☐ not done
4.72.3.	Leucocytes in the urine	☐ 0+	☐ 1+	☐ 2+	☐ 3+			☐ not done
4.72.4.	Blood in urine	☐ 0+	☐ 1+	☐ 2+	☐ 3+			☐ not done
4.72.5.	Ketones	☐ 0+	☐ 1+	☐ 2+	☐ 3+			☐ not done
4.72.6.	Nitrite	☐ 0+	☐ 1+	☐ 2+				☐ not done

CONCLUSION ON TODAYS EXAMINATION

4.73. **Ailment/disease** diagnosed today ☐ yes ☐ no

4.73.1. If yes, specify (≥1 "x")
☐ Anaemia ☐ Malaria ☐ Hypertension
☐ Urinary tract infection ☐ Preeclampsia
☐ Upper respiratory tract infect. ☐ Syphilis
☐ Reproductive tract infection ☐ Diabetes
☐ HIV ☐ Other

4.73.1.1. If other, specify:______________________________

4.74. **Treatment** prescribed today ☐ yes ☐ no

4.74.1. If yes, specify (≥1 "x")
☐ Coartem/ALU ☐ Quinine ☐ anti-helminth
☐ Iron ☐ Folic acid ☐ B12
☐ Hemovit ☐ Antibiotics ☐ Anti-HT
☐ Painkillers ☐ Other

4.74.1.1. If other or painkillers, specify: ______________________________

4.75. Specify name, dosage and duration of treatment:

4.75.1. 1st ______________________________

4.75.2. 2nd ______________________________

4.75.3. 3rd ______________________________

4.75.4. 4th ______________________________

OUTCOME OF DELIVERY (filled after delivery)

4.76.	Mother alive	☐ yes	☐ no	
4.77.	Baby alive	☐ yes	☐ no	
4.77.1.	If no, fresh or macerated stillbirth	☐ macerated	☐ fresh	
4.78.	Foetal distress (based on FHR during delivery)	☐ yes ☐ no	☐ unknown	
4.79.	Twins	☐ yes	☐ no	
4.79.1.	Type of Twins	☐ Mono-	☐ Di-chorial	
4.79.2.	Type of Twins cont.	☐ Mono-	☐ Di-amniotic	

4.79.3. Describe which twin came out first: ______________________________

____

4.80. Caesarian delivery ☐ yes ☐ no

4.80.1. If yes, specify why:______________________________

____

4.81.	Presentation of baby upon delivery	☐ head	☐ breech	☐ UN
4.81.1.	If twin, presentation of 2nd Newborn delivered	☐ head	☐ breech	☐ UN
4.82.	Pre-partum bleeding	☐ yes	☐ no	☐ UN
4.83.	Post-partum bleeding (>500ml)	☐ yes	☐ no	☐ UN
4.84.	Interventions **during** labour	☐ oxytocin<br>☐ instrumental delivery<br>☐ other	 <br>☐ None	☐ UN

4.84.1. If other, specify: ______________________________

4.85. Interventions **after** delivery ☐ oxytocin ☐ other ☐ none ☐ UN

4.85.1. If other, specify:______________________________

4.86. Other obstetrical problems: ______________________________

____

4.87. Other notes on delivery:______________________________

____

4.88. **Date** **(dd/mm/yyyy)/ time mother left** maternity ward: _ _ / _ _ / _ _ _ _ _ _ : _ _ ☐ UN/NA

4.89. **Mother's weight after delivery (kg):** _ _ _ , _ ☐ Not done

5. NEWBORNS PREG (ALWAS FILL IN)

5.1.	Date of investigation (dd/mm/yyyy)	_ _ / _ _ / _ _ _ _	☐ Not done
5.2.	Time of investigation	__ : __	
5.3.	Name of midwife/nurse/auxiliary worker: ________________________________		

5.4. <u>1st newborn (the one delivered first) (Data always collected)</u>

5.4.1.	Sex	☐ M	☐ F	☐ Unknown
5.4.2.	Weight (g) *(50g)*	1st _ _ _ _	2nd _ _ _ _	☐ Not done
5.4.3.	Length (infantometer) (cm) *(7mm)*	1st _ _ , _	2nd _ _ ,_	☐ Not done
5.4.4.	Head circumference (cm) *(5mm)*	1st _ _ , _	2nd _ _ ,_	☐ Not done
5.4.5.	Chest circumference (cm) *(5mm)*	1st _ _ , _	2nd _ _ ,_	☐ Not done
5.4.6.	Abdominal circumference (cm) *(5mm)*	1st _ _ , _	2nd _ _ ,_	☐ Not done
5.4.7.	Umbilical circumference (cm) *(5mm)*	1st _ _ , _	2nd _ _ ,_	☐ Not done
5.4.8.	Upper arm circumference (cm) *(2mm)*	1st _ _ , _	2nd _ _ ,_	☐ Not done
5.4.9.	Skinfold thickness triceps <u>(mm)</u> *(0,4mm)*	1st _ _ , _	2nd _ _ ,_	☐ Not done
5.4.10.	Skinfold thickness biceps <u>(mm)</u> *(0,4mm)*	1st _ _ , _	2nd _ _ ,_	☐ Not done
5.4.11.	Skinfold thickness subscapular <u>(mm)</u> *(0,4mm)*	1st _ _ , _	2nd _ _ ,_	☐ Not done
5.4.12.	Skinfold thickness thigh <u>(mm)</u> *(0,4mm)*	1st _ _ , _	2nd _ _ ,_	☐ Not done
5.4.13.	Length of foot (cm) *(2mm)*	1st _ _ , _	2nd _ _ ,_	☐ Not done
5.4.14.	APGAR score			
5.4.14.1.	1 min	_ _	☐ Not done	
5.4.14.2.	5 min	_ _	☐ Not done	
5.4.14.3.	10 min	_ _	☐ Not done	
5.4.15.	Appearance of amniotic fluid:	☐ Clear ☐ Bloody ☐ Opaque ☐ Tar-like/green (meconium) ☐ Unspecified		
5.4.16.	Malformations/ congenital disease	☐ yes	☐ no	☐ Unspecified
5.4.16.1.	If yes, details : ____			

5.5. <u>2nd newborn (if twins) (the one delivered last)</u>

5.5.1.	Sex	☐ M	☐ F	☐ Not known
5.5.2.	Weight (g) *(50g)*	1st _ _ _ _	2nd _ _ _ _	☐ Not done
5.5.3.	Length (infantometer) (cm) *(7mm)*	1st _ _ , _	2nd _ _ ,_	☐ Not done
5.5.4.	Head circumference (cm) *(5mm)*	1st _ _ , _	2nd _ _ ,_	☐ Not done
5.5.5.	Chest circumference (cm) *(5mm)*	1st _ _ , _	2nd _ _ ,_	☐ Not done
5.5.6.	Abdominal circumference (cm) *(5mm)*	1st _ _ , _	2nd _ _ ,_	☐ Not done
5.5.7.	Umbilical circumference (cm) *(5mm)*	1st _ _ , _	2nd _ _ ,_	☐ Not done
5.5.8.	Upper arm circumference (cm) *(2mm)*	1st _ _ , _	2nd _ _ ,_	☐ Not done
5.5.9.	Skinfold thickness triceps <u>(mm)</u> *(0,4mm)*	1st _ _ , _	2nd _ _ ,_	☐ Not done
5.5.10.	Skinfold thickness biceps <u>(mm)</u> *(0,4mm)*	1st _ _ , _	2nd _ _ ,_	☐ Not done
5.5.11.	Skinfold thickness subscapular <u>(mm)</u> *(0,4mm)*	1st _ _ , _	2nd _ _ ,_	☐ Not done
5.5.12.	Skinfold thickness thigh <u>(mm)</u> *(0,4mm)*	1st _ _ , _	2nd _ _ ,_	☐ Not done
5.5.13.	Length of foot (cm) *(2mm)*	1st _ _ , _	2nd _ _ ,_	☐ Not done
5.5.14.	APGAR score			
5.5.14.1.	1 min	_ _	☐ Not done	
5.5.14.2.	5 min	_ _	☐ Not done	
5.5.14.3.	10 min	_ _	☐ Not done	
5.5.15.	Appearance of amniotic fluid:	☐ Clear ☐ Bloody ☐ Opaque ☐ Tar-like/green (meconium) ☐ Unspecified		
5.5.16.	Malformations/ congenital disease	☐ yes	☐ no	☐ Unspecied
5.5.16.1.	If yes, details: ____			

DATA ENTRY:
1st entry done by:____Signature:_________________ date:_ _/_ _/_ _ _ _
2nd entry done by:____Signature:_________________ date:_ _/_ _/_ _ _ _

6. MACROSCOPICAL APPEARANCE OF PLACENTA PREG

6.1. Date of investigation/filling questionnaire (dd/mm/yyyy) _ _ / _ _ / _ _ _ _
6.2. **Time of delivery of Placenta** _ _ **:** _ _ ☐ Not known
6.2.1. **Time of delivery of 2nd placenta if twins** _ _ **:** _ _ ☐ Not known

6.3. Name of midwife/nurse/auxiliary worker: ____________________________________

6.4. Placenta processed (gross examination, and/or blood sampling and/or biopsies) ☐ yes ☐ no
6.4.1. If not, specify why ☐ Home delivery ☐ KCMC delivery ☐ Other
6.4.2. If other, specify: ____
6.4.3. **Start time of processing placenta** _ _ **:** _ _ ☐ Not appplicable

6.5. Placenta originating from a twin pregnancy ☐ yes ☐ no
6.5.1. If originating from a twin placenta, twin 1 processed ☐ yes ☐ no
6.5.2. twin 2 processed ☐ yes ☐ no

If originating from a twin placenta, go to form 7 for placental examination.

6.6. **Membranes** ☐ NOT DONE
6.6.1. Normal appearance: ☐ yes ☐ no
6.6.2. Colour : ☐ normal clear ☐ white ☐ yellow
6.6.3. Other observations: ____

6.7. **Umbilical cord** ☐ NOT DONE
6.7.1. Insertion: ☐ central/eccentric ☐ marginal ☐ velamentous
6.7.2. Distance from edge: _ _ cm
6.7.3. Length: _ _ cm
6.7.4. Diameter average thickness _ _ **mm**
6.7.5. Number of umbilical vessels: ☐ 2 ☐ 3
6.7.6. Spirality : ☐ 0 spirals of few ☐ normal ☐ many
6.7.7. Lesions (haematoma, thrombose, knot etc.): ______________________________

6.8. **Placenta** ☐ NOT DONE
6.8.1. Size (largest diameter): _ _ cm
6.8.2. Placental weight excluding membranes, cord, haemotom: _ _ _ _ g
6.8.3. Thickness of the placenta (measure in the center) _ _,_ cm ☐ big variation (not done)
6.8.4. Normal appearance ☐ yes ☐ no
6.8.5. If no, abnormal appear.: ☐ Extra-chorial ☐ Other shape
6.8.5.1. If extra-chorial : ☐ circummarginal ☐ circumvallate
6.8.5.2. If other shape (round/oval is normal), specify:_________________________
6.8.6. Torn ☐ yes ☐ no

6.9. **Chorial plate (fetal side)** ☐ NOT DONE
6.9.1. Colour: ☐ Blue ardoise ☐ Yellow ☐ Green
6.9.2. Cysts: ☐ yes ☐ no
6.9.3. Amniotic knotty (amnion nodosum): ☐ yes ☐ no
6.9.4. The configuration of the blood vasculature: ☐ normal ☐ abnormal
6.9.5. Other features: ________________________________

6.10. **Basal plate (maternal side)** ☐ NOT DONE
6.10.1. Intact (no cotyledons missing): ☐ yes ☐ no
6.10.2. Torn: ☐ yes ☐ no
6.10.3. Colour of red: ☐ dark ☐ whitish ☐ yellowish
6.10.4. Calcifications: ☐ yes ☐ no
6.10.5. Haematomes : ☐ yes ☐ no
6.10.6. Infarcts: ☐ yes ☐ no
6.10.7. Impression (sequelae after retroplacental infarct) ☐ yes ☐ no
6.10.8. Other features:____

6.11. **Placental parachyma (inside placenta tissue after cutting)** ☐ NOT DONE
6.11.1. Focal lesions (e.g. infarct) ☐ yes ☐ no
6.11.1.1. If yes, describe: ____
____

6.12. **Samples collected** ☐ NOT DONE
6.12.1. Umbilical cord blood collected in ☐EDTA 6ml ☐ Plain 6ml ☐EDTA 2ml ☐ Paxgene ☐ not done

6.12.1.1. Umbilical cord blood collected ☐ Before delivery of placenta ☐ After delivery of placenta

6.12.2. Placental impression smear ☐ done ☐ not done
6.12.3. Placental blood collected in ☐EDTA 2ml ☐ CPDA ☐ not done
CPDA only collected if venous mRDT positive
6.12.4. **Stereology/Epigenetics biopsies** ☐ done ☐ not done
6.12.5. Number of slices _ _
6.12.6. Total length of the added slices _ _ _ cm
6.12.6.1. Number circled on the random table _ _
6.12.7. Two cross sections of the umbilical cord (length 1-2cm), 2 and 10 cm above the placental disc ☐ yes ☐ no
6.12.8. Free membranes, a section of approximately 5 x 10 cm ☐ yes ☐ no
6.12.9. Biopsies collected if formalin (patoanatomy/stereology) ☐ yes ☐ no
6.12.9.1. If yes, number of sampled blocks _ _

6.12.10. Placental biopsies snap frozen in liquid nitrogen (epigenetics) ☐ yes ☐ no
6.12.10.1. **Time of epigenetics biopsy samples put in liquid nitrogen** _ _ **:** _ _
6.12.10.2. **Time of epigenetics biopsy samples put in -80 freezer** _ _ **:** _ _
6.12.10.3. **Date of transfer:** _ _ **/** _ _ **/**_ _ _ _´

6.12.11. 4 biopsies from normal tissue taken for **Centaflow container** ☐ yes ☐ no
6.12.12. Nb of biopsies from focal lesions for **Centaflow container** : _ _
6.12.12.1. Which lesions did you collect from: ____________________________
____
____

6.13. Notes:

____
____
____
____
____

6.14. **End time of processing of placenta:** _ _ **:** _ _

DATA ENTRY:

1st entry done by:______________________________Signature:_________________ date:_ _/_ _/_ _ _ _

2nd entry done by:______________________________Signature:_________________ date:_ _/_ _/_ _ _ _

7. MACROSCOPICAL APPEARANCE OF PLACENTA, if TWINS PREG

7.1. Appearance of the placenta ☐ two separate placenta ☐ one placental disc
7.1.1. If one placenta disc: ☐ two amniotic sacs ☐ one amniotic sac

IF one disc:
7.2. Placental weight, without membranes, cord, haematome _ _ _ _ g
7.3. Size of the placenta disc (if one disc) _ _ cm x _ _ cm
7.4. Thickness of the placenta (measured in the center) (if one disc) _ _ , _ cm
☐ big variation (not done)

Twin 1 = the twin delivered first, Twin 2 = the twin delivered last. The cords are marked during delivery as Twin 1 and Twin 2. During pregnancy Twin 1 will be the one most to the left, and twin 2 the one most to the right. Which twin that was leading in the birth channel during pregnancy, and therefore delivered first during vaginal delivery is marked at the last ultrasound scan.

IF two disc:
7.5. Placental weight, without membranes, cord, haematome (twin 1) _ _ _ _ g
7.6. Placental weight, without membranes, cord, haematome (twin 2) _ _ _ _ g
7.7. Size of the largest diameter (twin 1) _ _ cm
7.8. Size of the largest diameter (twin 2) _ _ cm
7.9. Thickness of the placenta (measured in the center) (twin 1) _ _, _ cm
☐ big variation (not done)
7.10. Thickness of the placenta (measured in the center) (twin 2) _ _ ; _ cm
☐ big variation (not done)

TWIN 1 PLACENTA:
Membranes
7.11. Normal appearance ☐ yes ☐ no
7.12. Colour ☐ normal clear ☐ white ☐ yellow
7.13. Other observations: ____________________
Umbilical cord
7.14. Insertion ☐central/eccentric ☐ marginal ☐ velamentous ☐ in septum
7.15. Distance from edge _ _cm
7.16. Length _ _ cm
7.17. Diameter, average thickness _ _ mm
7.18. Number of umbilical vessels ☐ 2 ☐ 3
7.19. Spirality ☐ 0 spirals or few ☐ normal ☐ many
7.20. Lesions (haematoma, thrombosis, knot etc): ____________________
Placenta
7.21. Normal appearance ☐ yes ☐ no
7.21.1. If no, abnormal appear: ☐ Extra-chorial ☐other shape
7.21.1.1. If extra-chorial: ☐ circummarginal ☐circumvallat
7.21.1.2. If other shape (round/oval is normal), specify:____________________
7.22. Torn ☐ yes ☐ no
Chorial plate (fetal side)
7.23. Colour ☐ Blue ardoise ☐ yellow ☐ green
7.24. Cysts ☐ yes ☐ no
7.25. Amniotic knotty (amnion nodosum) ☐ yes ☐ no
7.26. The configuration of the blood vasculature ☐ normal ☐ abnormal
7.27. Other features: ____________________
Basal plate (maternal side)
7.28. Intact (no cotelydons are missing) ☐ yes ☐ no
7.29. Torn ☐ yes ☐ no
7.30. Colour of red: ☐ dark ☐ whitish ☐ yellowish
7.31. Calcifications ☐ yes ☐ no
7.32. Haematomes: ☐ yes ☐ no
7.33. Infarcts ☐ yes ☐ no
7.34. Impression (sequelae after retroplacental infarct) ☐ yes ☐ no
7.35. Other features: ____________________

Placental parenchyma inside placenta tissue after cutting

7.36. Focal lesions (e.g. infarcts) ☐ yes ☐ no
7.36.1. If yes, describe: ____________________

TWIN 2 PLACENTA:

Membranes

7.37. Normal appearance ☐ yes ☐ no
7.38. Colour ☐ normal clear ☐ white ☐ yellow
7.39. Other observations: ____________________

Umbilical cord

7.40. Insertion ☐central/eccentric ☐ marginal ☐ velamentous ☐ in septum
7.41. Distance from edge _ _cm
7.42. Length _ _ cm
7.43. Diameter, average thickness _ _ mm
7.44. Number of umbilical vessels ☐ 2 ☐ 3
7.45. Spirality ☐ 0 spirals or few ☐ normal ☐ many
7.46. Lesions (haematoma, thrombosis, knot etc): ____________________

Placenta

7.47. Normal appearance ☐ yes ☐ no
7.47.1. If no, abnormal appear: ☐ Extra-chorial ☐other shape
7.47.1.1. If extrachorial: ☐ circummarginal ☐circumvallat
7.47.1.2. If other shape (round/oval is normal), specify:____________________
7.48. Torn ☐ yes ☐ no

Chorial plate (fetal side)

7.49. Colour ☐ Blue ardoise ☐ yellow ☐ green
7.50. Cysts ☐ yes ☐ no
7.51. Amniotic knotty (amnion nodosum) ☐ yes ☐ no
7.52. The configuration of the blood vasculature ☐ normal ☐ abnormal
7.53. Other features: ____________________

Basal plate (maternal side)

7.54. Intact (no cotelydons are missing) ☐ yes ☐ no
7.55. Torn ☐ yes ☐ no
7.56. Colour of red: ☐ dark ☐ whitish ☐ yellowish
7.57. Calcifications ☐ yes ☐ no
7.58. Haematomes: ☐ yes ☐ no
7.59. Infarcts ☐ yes ☐ no
7.60. Impression (sequelae after retroplacental infarct) ☐ yes ☐ no
7.61. Other features: ____________________

Placental parenchyma inside placenta tissue after cutting

7.62. Focal lesions (e.g. infarcts) ☐ yes ☐ no
7.62.1. If yes, describe: ____________________

SAMPLES COLLECTED

If one disc (document cord blood in section TWIN1 and TWIN2 below):

7.63. Section of septum ☐ yes ☐ no
7.64. Placental impression smear ☐ done ☐ not done
7.64.1. Placental blood collected in ☐ EDTA 2ml ☐ CPDA ☐ not done
CPDA only collected if venous mRDT positive
7.65. Tick which biopsies has been collected
☐ Umbilical cord (2+10cm above insertion)
☐ Membranes 5*10cm
☐ Two placenta random blocks in formalin
☐ Blocks for epigenetics in liquid nitrogen
7.66. Epigenetics biopsies,
7.66.1. **time of put in liquid nitrogen** _ _ : _ _
7.66.2. **time of transfer to -85 freezer** _ _ : _ _
7.66.3. **Date of transfer _ _ / _ _ / _ _ _ _**

If TWO DISC:

TWIN 1

7.67. Umbilical cord blood collected in ☐ EDTA 6ml ☐ Plain 6ml ☐ EDTA 2ml ☐ Paxgene ☐ not done

7.67.1. Umbilical cord blood collected ☐ Before delivery of placenta ☐ After delivery of placenta

7.68. Placental impression smear ☐ done ☐ not done

7.69. Placental blood collected in ☐ EDTA 2ml ☐ CPDA ☐ not done

CPDA only collected if venous mRDT positive

7.70. Tick which biopsies has been collected ☐ Umbilical cord (2+10cm above insertion) ☐ Membranes 5*10cm

7.71. **For stereology:**

7.71.1. Number of slices _ _

7.71.2. Total length of the added slices _ _ _ cm

7.71.2.1. Number circled on the random table _ _

7.71.3. Biopsies collected in formalin (patoanatomy/stereology) ☐ yes ☐ no

7.71.3.1. If yes, number of sampled blocks _ _

7.71.4. Placental biopsies snap frozen in liquiq nitrogen (epigenetics

7.71.4.1. **time of epigenetics biopsy samples put in liquid nitrogen** _ _ : _ _

7.71.4.2. **time of epigenetics biopsy samples transfer to -85 freezer** _ _ : _ _

7.71.4.3. **Date of transfer _ _ / _ _ / _ _ _ _**

7.72. Notes on Twin 1 placenta:____

____

____

____

TWIN 2

7.73. Umbilical cord blood collected in ☐ EDTA 6ml ☐ Plain 6ml ☐ EDTA 2ml ☐ Paxgene ☐ not done

7.73.1. Umbilical cord blood collected ☐ Before delivery of placenta ☐ After delivery of placenta

7.74. Placental impression smear ☐ done ☐ not done

7.75. Placental blood collected in ☐ EDTA 2ml ☐ CPDA ☐ not done

CPDA only collected if venous mRDT positive

7.76. Tick which biopsies has been collected ☐ Umbilical cord (2+10cm above insertion) ☐ Membranes 5*10cm

7.77. **For stereology:**

7.77.1. Number of slices _ _

7.77.2. Total length of the added slices _ _ _ cm

7.77.2.1. Number circled on the random table _ _

7.77.3. Biopsies collected in formalin (patoanatomy/stereology) ☐ yes ☐ no

7.77.3.1. If yes, number of sampled blocks _ _

7.77.4. Placental biopsies snap frozen in liquiq nitrogen (epigenetics

7.77.4.1. **time of epigenetics biopsy samples put in liquid nitrogen** _ _ : _ _

7.77.4.2. **time of epigenetics biopsy samples transfer to -85 freezer** _ _ : _ _

7.77.4.3. **Date of transfer _ _ / _ _ / _ _ _ _**

7.78. Notes on Twin 2 placenta:____

____

____

____

7.79. **End time of processing of placenta :** _ _ : _ _

8. Obstetric ultrasound form for GA estimation/uterine artery PREG

8.1. Clinic location: ☐ Korogwe District hospital ☐ Kerenge Dispensary ☐ Ngombezi Dispensary ☐ Lwengera Dispensary ☐ Majengo Dispensary ☐ Segera Dispensary ☐ Hale Dispensary ☐ Makuyuni Dispensary ☐ Chekelei Dispensary ☐ Other

8.1.1. If other, specify:____

8.2. Date of visit (dd/mm/yyyy) _ _ / _ _ / _ _ _ _

8.3. Filled in by : ____

8.4. Type of visit ☐ GA UL ☐ EMR ☐ Uterine artery + GA estimation

8.4.1. Nb of this type of visit _ _

8.5. Has CRF3 been filled ☐ yes ☐ no

8.6. Ultrasound (if abdominal, fill bladder prior to examination): ☐ vaginal ☐ abdominal

8.7. **GESTATION SAC** (only in very early pregnancy) ☐ done ☐ not done

8.7.1. Number : ☐ 1 ☐ 2 ☐ 3 ☐ 4

8.7.2. Appearance : ☐ vital ☐ non-vital

8.7.3. Second sac, appearance: ☐ vital ☐ non-vital

8.7.4. Diameter (Length+width-depth/3) (mm): _ _ , _

8.7.5. 2nd sac diameter (Length+width+depth/3) (mm): _ _ , _

8.7.6. Location : ☐ intra ☐ extra-uterine

8.7.7. 2nd sac location : ☐ intra ☐ extra-uterine

8.8. **1st EMBRYO (most to the left)**

8.8.1. Cardiac activity : ☐ yes ☐ no

8.8.2. Active movements : ☐ yes ☐ no

8.8.3. CRL (crown-rump length) (GA≤14) (mm) _ _ , _ ☐ not done

8.8.4. BPD (biparietal) (mm): _ _ ☐ not done

8.8.5. OFD (occiput-frontal diameter) (mm): _ _ ☐ not done

8.8.6. HC (head circumference) (mm): _ _ _ ☐ not done

8.8.7. TROPHOBLAST (placental tissue visualized): ☐ anterior ☐ posterior ☐ fundal

8.9. **2nd EMBRYO (most to the right)**

8.9.1. Cardiac activity: ☐ yes ☐ no

8.9.2. Active movements: ☐ yes ☐ no

8.9.3. CRL (crown-rump length) (GA≤14) (mm) _ _ ☐ not done

8.9.4. BPD (biparietal) (mm): _ _ ☐ not done

8.9.5. OFD (occiput-frontal diameter) (mm): _ _ ☐ not done

8.9.6. HC (head circumference) (mm): _ _ _ ☐ not done

8.9.7. TROPHOBLAST (placental tissue visualized): ☐ anterior ☐ posterior ☐ fundal

8.10. **DOPPLER (GA ≥11)**:

8.10.1.	Uterine artery		
8.10.1.1.	Pulsatile index (PI), right	_ , _ _	☐ not possible
8.10.1.1.1.	Resistance Index (RI), right	_ , _ _	☐ not possible
8.10.1.1.2.	S/D, right	_ , _ _	☐ not possible
8.10.1.2.	Right (notch):	☐ Yes	☐ No
8.10.1.3.	Measured at cervico-corporal site	☐ Yes	☐ No
8.10.1.4.	Pulsatile index (PI), left	_ , _ _	☐ not possible
8.10.1.4.1.	Resistance Index (RI), left	_ , _ _	☐ not possible
8.10.1.4.2.	S/D, left	_ , _ _	☐ not possible
8.10.1.5.	Left (notch):	☐ Yes	☐ No
8.10.1.6.	Measured at cervico-corporal site	☐ Yes	☐ No

8.11. GA based on ultrasound estimate today (*use CRL until GA 14*): _ _ weeks _ _ Days

8.12. FINAL GA: (leave blank if add. GA US is needed) _ _ weeks _ _ Days

8.12.1.	Pregnancy visible:	☐ yes	☐ no	
8.12.1.1.	If yes, foetus visible	☐ yes	☐ no	
8.12.1.2.	If foetus visible, FHR observed:	☐ yes	☐ no	
8.12.1.3.	If foetus visible, precise GA possible	☐ yes	☐ no	
8.12.2.	Twin pregnancy	☐ yes	☐ no	
8.12.2.1.	If yes, choriosity	☐ mono	☐ di	
8.12.2.2.	If yes, amniosity	☐ mono	☐ di	
8.12.3.	UtA evaluation possible (GA 11+0 - 14+0)	☐ yes	☐ no	
8.12.4.	Ectopic pregnancy :	☐ yes	☐ no	
8.12.5.	Other : ___________________________			
8.13.	Take a decision :			
8.13.1.	Excluded due to miscarriage/ectopic pregn.	☐ yes	☐ no	
8.13.2.	Excluded due to GA≥14 weeks at incl. (Case-control)	☐ yes	☐ no	☐NA
8.13.3.	Normal follow-up	☐ yes	☐ no	
8.13.4.	Repeat ultrasound necessary :	☐ yes	☐ no	
8.13.5.	Refer to specialist :	☐ yes	☐ no	

8.14. **CONCLUSIONS :** ____

____

____

If this is the first ultrasound performed in pregnancy:

All women without visible pregnancy (foetus) but positive UPT should be booked after 6 weeks for a new ultrasound, and a new form 8 will be filled. At this visit form 3 will also be filled, and a blood sample collected.

All women with a visible pregnancy, but GA<11 should be booked for a new ultrasound at GA 11-14 for accurate estimation of GA and for UtA evaluation, and a new form 8 will be filled. At this visit a form 3 will also be filled, and blood sample collected.

All women in the Cohort study with a GA =>20 weeks should also have a form 9 filled in at today's visit for estimation of foetal weight. Use the same measurements for variables present in both forms.

If ultrasound is not done at first contact in pregnancy or precise GA is not possible due to lie of foetus:

If US was not done or US indicates a GA≥11 (rough GA on US), but where it is not possible to get a precise GA due to lie of the fetus, the woman should be asked to come back the next day for a new ultrasound. Form 8 is only filled in the next day. In form 1 (Cohort study) and form 13 (Case-control study) it should be documented if ultrasound was not done or GA not possible due to lie of fetus.

If the women is already included in the study in very early pregnancy, but will be excluded at today's visit (2nd ultrasound for GA) due to miscarriage or ectopic pregnancy remember to fill Exclusion form 11 (both Case-control and Cohort study).

Next visit booked on: _ _ / _ _ / _ _ _ _, specify visit type: _____________

9. Obstetric ultrasound form PREG

This form should be used for ANV at GA 20, 26, 32 and 37, UL controls, as well as for all extra ANV and EMR visit were UL is indicated. Finally, at inclusion in Cohort study if GA is >=20 weeks.

9.1. Antenatal Clinic location:
- ☐ Korogwe District hospital ☐ Kerenge Dispensary
- ☐ Ngombezi Dispensary ☐ Lwengera Dispensary
- ☐ Majengo Dispensary ☐ Segera Dispensary
- ☐ Hale Dispensary ☐ Makuyuni Dispensary
- ☐ Chekelei Dispensary ☐ Other

9.1.1. If other, specify:____

9.2. Ultrasonographer's name: ____

9.3. Date (dd/mm/yyyy): _ _ / _ _ / _ _ _ _

9.4. **Gestational age**

9.4.1. By UL: _ _ Weeks _ _ days

9.4.2. By LMP: _ _ Weeks _ _ days

9.5. **Type of visit** ☐ ANV ☐ Extra ANV ☐ Incl. Cohort Study ☐ EMR ☐ only UL

9.5.1. **Number of this type of visit** (incl. today) _ _

9.5.2. **Specify reason for UL if not regular ANV** (GA 20 26, 32, 37):
- ☐ flow ctr. ☐ AFI ctr. ☐ overdue ☐ctr. praevia
- ☐ IUGR suspected (EFW/flow/AFI) ☐ EFW ctr. maternal disease
- ☐ vaginal bleeding ☐ decreased fetal movement
- ☐ abdominal trauma ☐ other

9.5.2.1. If other, specify: ____

9.6. Has CRF3 been filled ☐ yes ☐ no

9.6.1. Centaflow done today ☐ yes ☐ no

9.7. Ultrasound (if abdominal, fill bladder prior to examination): ☐ vaginal ☐ abdominal

9.8. **Number of foetuses** : _

9.8.1. If twin pregnancy, choriosity ☐ mono ☐ di

9.8.2. If twin pregnancy, amniosity ☐ mono ☐ di

9.9. **<u>1st FOETUS (the one most to the left)</u>**

9.9.1. Position/presentation ☐ Breech ☐ Head ☐ Limb

9.9.2. Fetal lie: ☐ longitudinal ☐ oblique ☐ transverse

9.9.3. Cardiac activity : ☐ yes ☐ no

9.9.4. Active movements : ☐ yes ☐ no

<u>1ST FOETUS BIOMETRICS</u>

9.9.5.	BPD (biparietal diameter) :	_ _ _ mm
9.9.6.	OFD (Occipito-frontal diameter):	_ _ _ mm
9.9.7.	HC (Head circumference):	_ _ _ mm
9.9.8.	TTD (abdominal transverse diameter):	_ _ _ mm
9.9.9.	APTD (abdominal diameter anterior - posterior) :	_ _ _ mm
9.9.10.	AC (abdominal circumference) :	_ _ _ mm
9.9.11.	FL (femur length) :	_ _ mm
9.9.12.	Foetal weight (Hadlock I – HC, AC, FL):	_ _ _ _ g
9.9.13.	Deviation from mean (if below mean)	_ _ , _ %

9.10. **<u>2nd FOETUS (the one most to the right)</u>**

9.10.1. Position/presentation ☐ Breech ☐ Head ☐ Limb

9.10.2. Fetal lie: ☐ longitudinal ☐ oblique ☐ transverse

9.10.3. Cardiac activity : ☐ yes ☐ no

9.10.4. Active movements : ☐ yes ☐ no

**2ND FOETUS BIOMETRICS**		
9.10.5.	BPD (biparietal diameter) :	_ _ _ mm
9.10.6.	OFD (Occipito-frontal diameter):	_ _ _ mm
9.10.7.	HC (Head circumference):	_ _ _ mm
9.10.8.	TTD (abdominal transverse diameter):	_ _ _ mm
9.10.9.	APTD (abdominal diameter anterior - posterior)	_ _ _ mm
9.10.10.	AC (abdominal circumference) :	_ _ _ mm
9.10.11.	FL (femur length) :	_ _ mm
9.10.12.	Foetal weight (Hadlock I – HC, AC, FL):	_ _ _ _ g
9.10.13.	Deviation from mean (if below mean)	_ _ , _ %

9.11. **PLACENTA :**
9.11.1. Position : ☐ anterior ☐ posterior ☐ fundal
9.11.2. If dichorionic gemelli, 2nd fetus position : ☐ anterior ☐ posterior ☐ fundal

9.12. **AMNIOTIC FLUID**:
9.12.1. Amniotic fluid: ☐ oligo ☐ normal ☐ polyhydram
9.12.1.1. 1st quadrant (right, upper): _ _ , _ cm
9.12.1.2. 2nd quadrant (left, upper): _ _ , _ cm
9.12.1.3. 3rd quadrant (rigth, lower): _ _ , _ cm
9.12.1.4. 4th quadrant (left, lower): _ _ , _ cm
9.12.2. Total: _ _ , _ cm
9.12.3. If diamniotic gemelli, AMNIOTIC FLUID, 2nd fetus: ☐ oligo ☐ normal ☐ polyhydram.
9.12.3.1. 1st quadrant (right, upper): _ _ , _ cm
9.12.3.2. 2nd quadrant (left, upper): _ _ , _ cm
9.12.3.3. 3rd quadrant (right, lower): _ _ , _ cm
9.12.3.4. 4th quadrant (left, lower): _ _ , _ cm
9.12.4. Total: _ _ , _ cm

9.13. **DOPPLER**:
9.13.1. Uterine artery
9.13.1.1. Pulsatile index (PI), right _ , _ _ ☐ not possible
9.13.1.1.1. Resistance Index (RI), right _ , _ _ ☐ not possible
9.13.1.1.2. S/D, right _ , _ _ ☐ not possible
9.13.1.2. Right (notch): ☐ Yes ☐ No
9.13.1.3. Measured at cervico-corporal site ☐ Yes ☐ No
9.13.1.4. Pulsatile index (PI), left _ , _ _ ☐ not possible
9.13.1.4.1. Resistance Index (RI), right _ , _ _ ☐ not possible
9.13.1.4.2. S/D, right _ , _ _ ☐ not possible
9.13.1.5. Left (notch): ☐ Yes ☐ No
9.13.1.6. Measured at cervico-corporal site ☐ Yes ☐ No
9.13.2. Umbilical artery pulsatile index (PI) (not at ANV GA 20) _ , _ _ ☐ not possible
9.13.3. if twins, 2nd fetus, Umbilical artery pulsatile index (PI) _ , _ _ ☐ not possible
9.13.3.1. Absent end diastolic flow (flow class 2b) ☐ Yes ☐ No
9.13.3.2. Absent flow in entire diastole(flow class 3a) ☐ Yes ☐ No
9.13.3.3. Reversed diastolic flow (flow class 3b) ☐ Yes ☐ No
9.13.4. UA Resistance Index (RI), right _ , _ _ ☐ not possible
9.13.5. UA S/D, right _ , _ _ ☐ not possible
9.13.6. If twin, 2nd fetus UA Resistance Index (RI), left _ , _ _ ☐ not possible
9.13.7. If twin, 2nd fetus S/D, left _ , _ _ ☐ not possible

9.14. **CONCLUSIONS :**____

9.15. Leading twin (A), note if it is twin 1(left) or 2 (right) _
9.16. Note any abnormalities:
9.16.1. Foetal death in utero : ☐ yes ☐ no
9.16.2. Placental insertion low (placenta praevia) ☐ yes ☐ no
9.16.3. Abnormal volume of amniotic fluid : ☐ yes ☐ no
9.16.4. SGA diagnosed (›- 15% of expected weight) ☐ yes ☐ no
9.16.5. Other: ________________________
9.17. Take a decision:
9.17.1. Normal follow-up : ☐ yes ☐ no
9.17.2. Repeat ultrasound necessary : ☐ yes ☐ no
9.17.3. Refer to specialist : ☐ yes ☐ no

Next visit booked on: _ _ / _ _ / _ _ _ _, specify visit type: ____________

10. REFERRAL FORM REF / / PREG

10.1. Clinic location : ☐ Korogwe District Hospital ☐ Other
10.1.1. If other, specify: ____
10.2. Form completed by : ____
10.3. Date of referral (dd/mm/yyyy): _ _ / _ _ / _ _ _ _
10.4. Referral clinic/hospital ☐ Korogwe District Hospital ☐ KCMC
☐ Bombo Referral hospital ☐ Other
10.4.1. If other, give details :____
10.5. Referral for:
☐ admission, medical treatment ☐ 2nd opinion
☐ admission, surgery ☐ other
10.6. Details :____
____
____
____
____
____
____
____
____
____
____
10.7. Outcome of referral :
____
10.8. Results from additional tests :
____
____
____
____
____
____
____
____
____
____
10.9. Confirmed final diagnosis :____
10.10. Treatment on discharge :
____
____
____
____
____
____
____
____
____
____
10.11. PONA follow-up: ☐ Mother delivered ☐ continue ☐ excluded
10.12. Date of discharge (dd/mm/yyyy): _ _/_ _/_ _ _ _

DATA ENTRY:
1st entry done by:___________________________Signature:_______________ date:_ _/_ _/_ _ _ _
2nd entry done by:___________________________Signature:_______________ date:_ _/_ _/_ _ _ _

11. EXCLUSION FORM PREG

11.1. Clinic location: ☐ Korogwe District Hospital ☐ Other

11.1.1. If other, specify :________________________________

11.2. Date (dd/mm/yyyy): _ _ / _ _ / _ _ _ _

11.3. Completed by : ____

11.4. EXCLUSION DUE TO :

☐ Moving out of the study area ☐ Lost to follow-up

☐ Medical reason ☐ Refusal/withdraw of consent

☐ Other

☐ US with GA>14 weeks at inclusion (case-control study only, US done another day than inclusion. If US is done on the same day as inclusion exclusion due to GA >14 is only documented in form 13.

11.4.1. If exclusion due to medical reason, give details :

☐ Miscarriage before GA 11-14 scan ☐ Miscarriage after GA 11-14 scan

☐ Mother has died ☐ Other

11.5. Explanatory notes : ____

____

____

____

____

____

____

____

____

DATA ENTRY:

1st entry done by:____Signature:________________ date:_ _/_ _/_ _ _ _

2nd entry done by:____Signature:________________ date:_ _/_ _/_ _ _ _

12. SCREENED WOMEN NOT HAVING FORM 2 FROM PRE-PREGN. PART (PREG)

12.1. Name of study worker filling the form:____

12.2. Date of filling CRF: (dd/mm/yyy) _ _ / _ _ / _ _ _ _

MATERNAL DEMOGRAPHIC DATA

12.3. **Ethnic** group ☐ Sambaa ☐ Zigua ☐ Pare ☐ Bondei ☐ Other

12.3.1. if other, specify: ____

12.4. **Education** ☐ none ☐ primary school partially completed ☐ primary school finished ☐ secondary school and higher

12.4.1. Do you know how to read and write ☐ yes ☐ no

12.5. **No. of siblings** (genetic brothers and sisters, same father/mother)_ _ ☐ unknown

12.6. **Residence before age 15** for the majority of the time ☐urban ☐rural ☐urban/rural ☐ unknown

12.7. **Religion** ☐ Islamic ☐Catholic ☐ Lutheran ☐ Angikana ☐ Hindi ☐Other

12.7.1. If other, specify:____

12.8. **GRAVIDITY** (including the present pregnancy): _ _

12.8.1. Number of previous pregnancies (gravidae) _ _

12.8.2. Number of previous deliveries (parity) _ _

12.8.2.1. Nb. of times delivering twins _ _

12.8.2.2. Nb. of live births (nb of babies born; singleton=1 & twins=2 if both live born) _ _

12.8.2.3. Nb. of still births (nb of babies born; singleton=1 & twins=2 if both stillborn) _ _

12.8.3. Number of interrupted pregnancies (miscarriages) _ _

12.8.4. Number of extrauterine pregnancies (mimba nje ya kizazi) _ _

12.8.5. Date of termination of the last pregnancy (incl. delivery, miscarriage, extrauterine) (dd/mm/yyyy) _ _ /_ _ /_ _ _ _

12.8.6. Date of last delivery (dd/mm/yyyy) _ _ /_ _ /_ _ _ _

MEDICAL HISTORY and EXAMINATION

Previous pregnancies (skip if never been pregnant before)

12.9. Maternal disease during previous pregnancies, diagnosed by medical personnel (≥1 "x")

☐ preeclampsia (dalili za kifafa cha mimba)
☐ pregnancy-induced HT(shinikizo la damu linalotokana na ujauzito)
☐ diabetes (kisukari) ☐ other ☐ severe anaemia (upungufu wa damu kali)
☐ none ☐ Don't know

12.9.1. If other, specify:____

Menstrual pattern and family planning <u>BEFORE</u> getting pregnant

12.10. Age of menarche (first menstrual period/ kuvunja ungo) _ _ ☐ unknown

12.10.1. Had amenorrhea up to getting pregnant ☐ yes☐ no

12.10.1.1. If yes, state reason (e.g. since last delivery, recent breast-feeding, just stopped family planning):____

____

Answer the following questions according to menstrual pattern before pregnancy/amenorrhea

12.11. Length of cycle before getting pregnant (days) min_ _ _ max_ _ _ ☐ unknown

12.12. Regular period (max variation 20days) ☐ yes ☐ no ☐ unknown

12.13. Days of bleeding _ _ ☐ unknown

12.13.1. Degree of bleeding day 1 and 2
☐ mild (change pads 1-2 times per day)
☐ moderate (change pads 3-5 times per day)
☐ severe (change pads ≥6 times per day)
☐ Don't know

12.13.2. Degree of bleeding remaining days
☐ mild (change pads 1-2 times per day)
☐ moderate (change pads 3-5 times per day)
☐ severe (change pads ≥6 times per day)
☐ Don't know

12.14. **Which methods of family planning** have you previously used (use (x≥1 if applicable)
☐ oral contraceptive (vidonge) ☐ condom
☐ hormone injectable (sindano) ☐ implant (kitiji)
☐ periodic abstinence ☐ withdrawal
☐ IUD (kitanzi) ☐ none ☐ other

12.14.1. If other, specify: ____

12.14.2. Used more than one method of family planning ☐ yes ☐ no

12.14.2.1. If more than one method, state the most recent modern method:
☐ oral contraceptive (vidonge) ☐ condom ☐ hormone injectable (sindano)
☐ implant (kitiji) ☐ IUD (kitanzi) ☐ other ☐none

12.14.2.2. If more than one method, state the most recent traditional method:
☐ periodic abstinence ☐ withdrawal ☐ other ☐none

12.14.3. When did you terminate using the most recent modern method_ _ / _ _ / _ _ _ _ ☐ UN
☐ Did not stop, got pregnant while using condoms)

12.14.3.1. When did you terminate using the most recent traditional method _ _ / _ _ / _ _ _ _ ☐ UN
☐ Did not stop, got pregnant while using traditional methods

If used oral contraceptives, hormone injectable, implant, IUD:

12.14.4. For how long have you used the method _ _ years _ _ months _ _ weeks
12.14.4.1. State method: ______________________
12.14.5. For how long have you used the method _ _ years _ _ months _ _ weeks
12.14.5.1. State method: ______________________
12.14.6. For how long have you used the method _ _ years _ _ months _ _ weeks
12.14.6.1. State method: ______________________
12.14.7. Notes on family planning/menstrual pattern:______________________________

____
____
____
____

12.15. **<u>Currently</u> tried to get pregnant** for how long _ years _ _ months _ _ weeks ☐ unknown

12.16. **How many children** do you prefer/would you like to have (ungependa kuwa na watoto wangapi)
_ _ ☐ don't know/refuse to answer

12.17. Who decide the number of children you can have
☐ Me and my partner ☐ Me, alone
☐ My partner, alone ☐ Other

12.17.1. If other, specify: ____

Other chronic diseases

12.18. Any close relatives with a chronic disease (genetic sister, brother, father, mother, grandparents, aunt, uncle) with (put >1 "x" if needed)
☐ Diabetes (kisukari) ☐ Cardiac disease
☐ Hypertension (shinikiza la damu) ☐ Severe undernutrition (utapiamlo mkali)
☐ Chronic anaemia(e.g.sickle cell, thalassemia)(upungufu wa damu kwa muda mrefu)
☐ Other ☐ No known disease

12.18.1. If yes or other, specify which relative and disease__________________________
____

DATA ENTRY:
1st entry done by:______________________________________Signature:_________________ date:_ _/_ _/_ _ _ _
2nd entry done by:______________________________________Signature:_________________ date:_ _/_ _/_ _ _ _

13. Mother's Inclusion Form 13, Case-Control Study (PREG)

13.1. Antenatal Clinic location (where CRF is filled):

☐ Korogwe District Hospital ☐ Kerenge Dispensary
☐ Ngombezi Dispensary ☐ Lwengera Dispensary
☐ Majengo Dispensary ☐ Segera Dispensary
☐ Hale Dispensary ☐ Makuyuni Dispensary
☐ Chekelei Dispensary ☐ Other

13.1.1. If other, specify:____

13.2. Name of study worker filling the form:______________________________

13.3. Date of filling CRF(*when filling of CRF is started)*: (dd/mm/yyyy) _ _ / _ _ / _ _ _ _

13.4. Mother's surname: ____

13.5. Mother's first and second name: ____________________________

13.6. Mother's age: _ _ years ☐ unknown

13.7. Date of birth according to the woman(dd/mm/yyyy) _ _ / _ _ / _ _ _ _

13.8. Positive urine pregnancy test ☐ yes ☐ no

13.9. UPT done at satellite dispensary/field site and referred ☐ yes ☐ no

13.9.1. If yes, state name of satellite dispensary/field site: ______________________

CURRENT PREGNANCY DETAILS (on the day of filling the CRF (date=1.3))

13.10. **Hemoglobin level** at today's visit (on hemocue) _ _,_ g/dl

13.11. **Intrauterine pregnancy confirmed on ultrasound**

☐ yes ☐ pregnancy not visible ☐ Extrauterine
☐ US not done ☐ GA not possible due to lie of foetus

13.11.1. If YES, gestational age by ultrasound: _ _ weeks _ days

13.11.2. If US not done, state reason:______________________________

13.12. **Symphysis-fundal height** _ _ cm ☐ not palpable

13.13. **Date of last menstrual period** (LMP): (dd/mm/yyyy) _ _ / _ _ / _ _ _ _ ☐ Unknown

13.13.1. Gestational age by last menstrual period (LMP): _ _ weeks _ days

13.14. **Is this the first ANC visit** ☐ yes ☐ no

13.14.1. If not, how many previous visits (excluding the current visit): _ _

ELIGIBILITY FOR CASE-CONTROL STUDY

13.15.	Previously included in PONA Study I/Cohort study	☐ yes ☐ **no**	Exclusion if yes
13.16.	Previously included in PONA Case-control Study	☐ yes ☐ **no**	Exclusion if yes
13.17.	Urine pregnancy test positive	☐ **yes** ☐ no	
13.18.	Balanced 1:1:1 Case:Control ratio	☐ **yes** ☐ no	Exclusion if no
13.19.	Gestational age	☐ **≤14** ☐ >14	Exclusion if >14
13.20.	Foetus visible, but non-viable pregnancy (miscarriage)	☐ yes ☐ **no**	Exclusion if yes
13.21.	Delivery planned at the Hospital	☐ **yes** ☐ no	Exclusion if no
13.22.	Consents to participate in the Case-control study	☐ **yes** ☐ no	Exclusion if no

13.23. ☐ **INCLUSION** ☐ **REFUSAL** ☐ **EXCLUSION**

In case of inclusion,

13.23.1. Included as ☐ Group A (Hb≤8g/dL) ☐ Group B (8.1-10.9g/dL)☐ control (Hb≥11g/dL)

13.23.2. **If ultrasound with precise GA not done,** state date of new ultrasound _ _ / _ _ / _ _ _ _

13.23.2.1. If booked for ultrasound, specify where: ______________________

In case of exclusion/refusal,

13.23.3. **Excluded due to imbalance of 1:1:1 case-control design:** ☐ yes ☐ no

13.23.4. **In case of refusal or exclusion**, state reason: ______________________

13.24. **STATE COHORT STUDY ID NUMBER: FN _ _ _ _ _** ☐ Do not have

DATA ENTRY:

1st entry done by:________________________Signature:__________ date:_ _/_ _/_ _ _ _

2nd entry done by:________________________Signature:__________ date:_ _/_ _/_ _ _ _

14. Mother's Inclusion Case-Control Study (PREG)

14.1. Name of study worker filling the form: ____
14.2. Date of filling CRF: (dd/mm/yyyy) _ _ / _ _ / _ _ _ _

MATERNAL DEMOGRAPHIC DATA

14.3. **Ethnic group** ☐ Sambaa ☐ Zigua ☐ Pare ☐ Bondei ☐ Other
14.3.1. If other, specify: ____
14.4. **Home address**
14.4.1. Street name:____
14.4.2. Street chairman:____
14.4.3. Village: ____
14.4.4. Subvillage:____
14.4.5. Subvillage chair:____
14.4.6. District/ward: ____
14.4.7. Ten Cell leader:____
14.4.8. Name of Husband/partner if living together:________________________________
14.4.9. Head of household:____
14.4.9.1. House number:________________________________
14.4.9.2. Known as Mama________________________________
14.4.10. Phone number (woman or partner)________________________________
14.5. **Residence:**
14.5.1. Type of roof on the house: ☐ Bati ☐ Tin (madebe) ☐Thatch ☐ Mixed Thatch/Tin ☐ Other
14.5.1.1. if other, specify: ____
14.5.2. How many people usually sleep in your residence _ _
14.5.3. Who owns the house you live in ☐ self/spouse – built ☐ inherited ☐ rental ☐ others
14.5.3.1. if others, specify:____
14.5.4. Type of home toilet: ☐ flush ☐ pit latrine(choo cha shimo) ☐ no toilet
14.5.5. Source of water ☐ tap(bombani) ☐ well (kisima cha mdundiko) ☐ river/stream (mto) ☐ gutter water in tank (maji ya paa) ☐ pond/pool (bwawa) ☐ other
14.5.5.1. if other, specify: ____
14.5.5.2. if tap, well, or gutter water ☐ Private ☐ Public
14.6. **Education** ☐ none ☐ primary school partially completed ☐ primary school finished ☐ secondary school and higher
14.6.1. Do you know how to read and write ☐ yes ☐ no
14.7. **Current occupation** ☐ Professional ☐ Business ☐ Service ☐ Farmer ☐ Housewife/ working at home ☐ Other
14.7.1. If other, specify: ____
14.7.2. Do you use chemicals for pest/diseases in your work ☐ yes ☐ no
14.7.2.1. If yes, specify:____
14.8. **No. of siblings** (genetic brothers/sisters, same father/mother) _ _ ☐ unknown
14.9. **Residence before age 15** for the majority of the time ☐urban ☐rural ☐urban/rural ☐ unknown
14.10. **Marital status** ☐ Married ☐ Divorced ☐ Separated ☐ Never married ☐ Widow ☐ Refuse answer
14.10.1. If divorced/separated/widow/never married, do you currently have a partner
☐ yes, cohabiting
☐ yes, but not cohabiting
☐ no
14.11. **Religion** ☐ Islamic ☐Catholic ☐ Lutheran ☐ Angikana ☐ Hindi ☐Other
14.11.1. If other, specify:____
14.12. **Who will take care of you and the newborn** ☐ Myself ☐ Parents
☐ Me and my husband/partner
☐ Others
14.12.1. If others, specify: ____

PATERNAL DEMOGRAPHIC DATA

14.13. Agree to answer questions about the father of the coming newborn ☐ yes ☐ no
14.14. Name of father of the coming newborn:______________________________
14.15. Age of father of the coming newborn _ _ years ☐ unknown
14.16. **Ethnic group** ☐ Sambaa ☐ Zigua ☐ Para ☐ Bondei ☐ Other ☐ unknown
14.16.1. if other, specify: ______________________________
14.17. **Education** ☐ none ☐ primary school partially completed ☐ primary school finished ☐ ≥secondary school ☐ unknown
14.17.1. Does he knows how to read and write ☐ yes ☐ no ☐ unknown
14.18. **Religion** ☐ Islamic ☐ Catholic ☐ Lutheran ☐ Angikana ☐ Hindi ☐Other
14.18.1. If other, specify:______________________________
14.19. **Current occupation** ☐ Professional ☐ Business ☐ Service ☐ Farmer ☐ Other ☐ unknown
14.19.1. If other, specify:______________________________

14.20. **GRAVIDITY** (including the present pregnancy): _ _
14.20.1. Number of previous pregnancies (gravidae) _ _
14.20.2. Number of previous deliveries (parity) _ _
14.20.2.1. Nb. of times delivering twins _ _
14.20.2.2. Nb. of live births (nb of babies born; singleton=1 & twins=2 if both live born) _ _
14.20.2.3. Nb. of still births (nb of babies born; singleton=1 & twins=2 if both stillborn) _ _
14.20.3. Number of interrupted pregnancies (miscarriages) _ _
14.20.4. Number of extrauterine pregnancies (mimba nje ya kizazi) _ _
14.20.5. Date of termination of the last pregnancy (incl. delivery, miscarriage, extrauterine) (dd/mm/yyyy) _ _ /_ _ /_ _ _ _
14.20.6. Date of last delivery (dd/mm/yyyy) _ _ /_ _ /_ _ _ _

NUTRITIONAL AND PHYSICAL HABITS

14.21. Has the “IPAQ questionnaire” been filled ☐ yes ☐ no
14.22. Has the “24hours Recall questionnaire” been filled ☐ yes ☐ no

MEDICAL HISTORY

Previous pregnancies (skip if never been pregnant before)

14.23. Maternal disease during previous pregnancies, diagnosed by medical personnel (≥1 “x”)
☐ preeclampsia (dalili za kifafa cha mimba)
☐ pregnancy-induced HT(shinikizo la damu linalotokana na ujauzito)
☐ diabetes (kisukari) ☐ other ☐ severe anaemia (upungufu wa damu kali) ☐ none ☐ Don’t know
14.23.1. If other, specify:______________________________

Menstrual pattern and family planning <u>BEFORE</u> getting pregnant

14.24. **Age of menarche** (first menstrual period/ kuvunja ungo) _ _ ☐ unknown
14.25. Had amenorrhea up to getting pregnant ☐ yes ☐ no
14.25.1. If yes, state reason (e.g. since last delivery, recent breast-feeding, just stopped family planning):______________________________

Answer the following questions according to menstrual pattern before pregnancy/amenorrhea

14.26. **Length of cycle** before getting pregnant (days) min_ _ _ max_ _ _ ☐ unknown
14.27. **Regular period** (max variation 20days) ☐ yes ☐ no ☐ unknown
14.28. **Days of bleeding** _ _ ☐ unknown
14.28.1. Degree of bleeding day 1 and 2
☐ mild (change pads 1-2 times per day)
☐ moderate (change pads 3-5 times per day)
☐ severe (change pads ≥6 times per day)
☐ Don’t know

14.28.2. Degree of bleeding remaining days
☐ mild (change pads 1-2 times per day)
☐ moderate (change pads 3-5 times per day)

☐ severe (change pads ≥6 times per day)
☐ Don't know

14.29. **Which methods of family planning** have you previously used (use (x≥1 if applicable)
☐ oral contraceptive (vidonge) ☐ condom
☐ hormone injectable (sindano) ☐ implant (kitiji)
☐ periodic abstinence ☐ withdrawal
☐ IUD (kitanzi) ☐ none ☐ other

14.29.1. If other, specify: ____________________

14.30. **Used more than one method of family planning** ☐ yes ☐ no

14.30.1. If more than one method, state the most **recent <u>modern</u> method**:
☐ oral contraceptive (vidonge) ☐ condom ☐ hormone injectable (sindano)
☐ implant (kitiji) ☐ IUD (kitanzi) ☐ other ☐none

14.30.2. If more than one method, state the most **recent <u>traditional</u> method**:
☐ periodic abstinence ☐ withdrawal ☐ other ☐none

14.31. **When did you terminate using the most recent modern method** _ _ / _ _ / _ _ _ _
☐ Did not stop, got pregnant while using modern methods

14.32. **When did you terminate using the most recent traditional method** _ _ / _ _ / _ _ _ _
☐ Did not stop, got pregnant while using traditional methods

<u>If used oral contraceptives, hormone injectable, implant, IUD:</u>

14.33. For how long have you used the method _ _ years _ _ months _ _ weeks

14.33.1. State method: ____________________

14.34. For how long have you used the method _ _ years _ _ months _ _ weeks

14.34.1. State method: ____________________

14.35. For how long have you used the method _ _ years _ _ months _ _ weeks

14.35.1. State method: ____________________

14.36. Notes on family planning/menstrual pattern:____________________

____

____

14.37. **<u>Currently</u> tried to get pregnant** for how long_ _ years _ _ months _ _ weeks ☐ unknown

14.38. **How many children do you prefer/would** you like to have (ungependa kuwa na watoto wangapi) _ _ ☐ don't know/refuse to answer

14.39. **Who decide the number of children** you can have
☐ Me and my partner ☐ Me, alone
☐ My partner, alone ☐ Other

14.39.1. If other, specify: ____________________

Anaemia (upungufu wa damu)

14.40. Has chronic anaemia now (diagnosed by medical staff) ☐ yes, known irreversible cause
☐ yes, lasting>6months
☐ no
☐ don't know/not tested

14.40.1. If yes, cause known ☐ yes ☐ no

14.40.2. If yes, specify (e.g. sickle cell, severe malnutrition)____________________

14.41. Temporary anaemia diagnosed within the last 4 months ☐ yes ☐ no

14.41.1. Nb. of times the last 4 month _ _

14.41.2. If yes, state first day of the last episode (dd/mm/yyyy) _ _/_ _/_ _ _ _

14.41.2.1. Hemoglobin measurement done ☐ yes ☐ no

14.41.2.2. If yes, state the value (g/dL) _ _, _ ☐ unknown

14.42. Received treatment for anemia within the last 4 months ☐ yes ☐ no

14.42.1. If yes, specify(put >1 if needed): ☐ Iron ☐ Folic ☐ B12
☐ Anti-helminths ☐ Hemovit
☐ Other

14.42.1.1. If other, specify____________________

14.42.2. State when treatment was started _ _ /_ _ /_ _ _ _

14.42.3. No. of tablets/day _ _ ☐ unknown

14.42.4. Dose per tablet _ _ _ _ ☐ unknown

14.42.5. State how long treatment was received: _ _ months _ _ weeks _ _ days☐ unknown

14.42.6. Additional notes:____________________

Other chronic diseases

14.43. **Diagnosed with a chronic illness** by medical personnel (ask specifically about: diabetes type I or II (kisukari), kidney (figo), heart (moyo), thyroid (goiter) or lung (mapafu) disease, cancer (saratani), hypertension (shinikizo la damu), epilepsy (kifafa), lymphatic filariasis (matende), gastric ulcer (vidonda vya tumbo), autoimmune disorders (e.g. rheumatoid arthritis (rheumotism), chronic diarrhea (kuharisha kwa muda mrefu))

☐ yes ☐ no

14.43.1. if yes, specify____

14.44. **Any close relatives with a chronic disease** (genetic sister, brother, father, mother, grandparents, aunt, uncle) with (put >1 "x" if needed)

☐ Diabetes (kisukari) ☐ Cardiac disease
☐ Hypertension (shinikiza la damu) ☐ Severe undernutrition (utapiamlo mkali)
☐ Chronic anaemia(e.g.sickle cell, thalassemia)(upungufu wa damu kwa muda mrefu)
☐ Other ☐ No known disease

14.44.1. If yes or other, specify which relative and disease__________________________

____

14.45. **Gynaecological disorders**

14.45.1. **Ever diagnosed with a pelvic inflammatory disease** by medical personnel

☐ yes ☐ no

14.45.2. **Diagnosed with any gynecological disorder** by medical personnel (e.g. endometriosis, fibroma, cysts on the ovary, septum in uterus)

☐ yes ☐ no

14.45.2.1. If yes, specify: ____

14.45.3. **Ever had abdominal surgery performed** (e.g. surgery for appendicaitis (kidole tumbo), gall bladder stone (mawe kwenye kibofu cha nyongo), uterus fibroma, ovary cysts, caesarean (kujifungua kwa upasuaji) ☐ yes ☐ no

14.45.3.1. If yes, specify: ____

14.46. **HIV status** according to the woman:

☐ Unwilling to respond
☐ Positive
☐ Negative, will be re-testing today
☐ Negative, do not want re-testing
☐ Do not know, will be tested today
☐ Do not know, do not want testing

14.46.1. If positive, attending a CTC ☐ yes ☐ no

14.46.1.1. If yes, where:____

Malaria

14.47. **Does she have a bednet yes/no** ☐ yes ☐ no

14.47.1. If yes, did she use it last night? ☐ yes ☐ no

14.47.2. Impregnated bednet ☐ yes ☐ no ☐ unknown

14.47.3. Obtained from national program ☐ yes ☐ no ☐ unknown

14.48. **Nb. of malaria attacks since the beginning of the current pregnancy?**

☐ 0 ☐ 1 ☐ 2 ☐ 3 ☐ other

14.48.1. If other number, specify: ___

14.48.2. Date of the 1st malaria attack: (dd/mm/yyyy) _ _ / _ _ / _ _ _ _

14.48.3. Date of the 2nd malaria attack: (dd/mm/yyyy) _ _ / _ _ / _ _ _ _

14.48.4. Date of the 3rd malaria attack: (dd/mm/yyyy) _ _ / _ _ / _ _ _ _

14.49. 1st malaria attack:

14.49.1. Was malaria confirmed with a blood test ☐ yes ☐ no ☐ unspecified

14.49.2. What treatment did you take?

☐ Quinine ☐ SP/ Metakelfin
☐ Chloroquine ☐ Herbal remedy
☐ None ☐ unspecified ☐ other ☐ ALU

14.49.2.1. If other, details: ____

14.50. 2nd malaria attack:

14.50.1. Was malaria confirmed with a blood test ☐ yes ☐ no ☐ unspecified

14.50.2. What treatment did you take?

☐ Quinine ☐ SP/ Metakelfin
☐ Chloroquine ☐ Herbal remedy
☐ None ☐ unspecified ☐ other ☐ ALU

14.50.2.1. If other, details: ____

14.51. 3rd malaria attack:

14.51.1. Was malaria confirmed with a blood test ☐ yes ☐ no ☐ unspecified
14.51.2. What treatment did you take? ☐ Quinine ☐ SP/ Metakelfin ☐ Chloroquine ☐ Herbal remedy ☐ None ☐ unspecified ☐ other ☐ ALU
14.51.2.1. If other, details: ____________________

14.52. **IPT-SP** taken during the current pregnancy ☐ no ☐ 1 dose ☐ 2 doses ☐ 3 doses

14.53. **Other malaria chemoprophylaxis** during current pregnancy? ☐ yes ☐ no
14.53.1. If yes, what treatment was taken (≥1)? ☐ Chloroquine ☐ Herbal ☐ unspecified ☐ other
14.53.1.1. If other, details : ____________________

Current usage of medicine
14.54. Currently taking medication? ☐ yes ☐ no
14.54.1. If yes, give details (≥1 “x”): ☐ antibiotics ☐ antimalarial ☐ iron ☐ antiretrovirals ☐ folic acid ☐ B12 ☐ antihelminths ☐ traditional ☐ Hemovit ☐ antihypertensive ☐ painkillers ☐ other
14.54.1.1. If other or painkillers,specify: ____________________
14.54.1.2. If, traditional specify: ____________________
14.54.1.3. Dosage of these drugs: ____________________
____

Substance abuse:
14.55. Smoker ☐ yes ☐ no
14.55.1. If yes, number of cigarettes per _ _ month _ _ week _ _ day
14.56. Usage of alcohol during this pregnancy ☐ yes ☐ no
14.56.1. If yes, how many items per _ _ month _ _ week _ _ day

14.57. Usage of caffeine beverages ☐ yes ☐ no
14.57.1. If Coke, how many items (bottles) per _ _ month _ _ week _ _ day
14.57.2. If coffee, how many items (cups) per _ _ month _ _ week _ _ day
14.57.3. If tea, how many items (cups) per _ _ month _ _ week _ _ day

MEDICAL EXAMINATION *(all * should be filled by CO/AMO/MD)*
14.58. Woman’s height (cm) _ _ _
14.59. Woman's weight (kg) _ _ _ , _
14.60. Waist circumference (cm) (at top of iliac crest) _ _ _ , _
14.61. Hip circumference (cm) (widest portion of the buttocks) _ _ _ , _
14.62. MUAC (cm) _ _ , _
14.63. Skinfold thickness of triceps (mm) _ _ , _
14.64. Blood pressure (BP) (mmHg)
14.64.1. 1st BP, left arm _ _ _ / _ _ _
14.64.2. 1st BP, right arm _ _ _ / _ _ _
14.64.3. Difference in left and right arm (max 20 syst.; 10 diast.) _ _ / _ _
14.64.4. Reference arm (the arm with the highest BP) ☐ Right ☐ Left
14.64.5. Reference arm (the arm with the highest BP), 2nd BP _ _ _ / _ _ _
14.64.6. Mean BP (1st and 2nd BP for reference arm) _ _ _ / _ _ _
14.64.6.1. If, mean BP >140/90 repeat after 4 hours, BP at repeat _ _ _ / _ _ _ ☐ not done
14.64.6.2. After how many hours was repeat BP performed _ _ ☐ not done
14.64.7. Pulse (use last BP measurement) _ _ _
14.65. Axillary temperature (°C) _ _ , _
14.66. * Headache ☐ yes ☐ no
14.67. * Visual disturbances ☐ yes ☐ no
14.67.1. If yes, specify: ____________________
14.68. * Dizziness ☐ yes ☐ no
14.69. * Denuded/glossy tongue (glossitis) ☐ yes ☐ no
14.70. * Commisura of the lips (cheilosis, fissure) ☐ yes ☐ no
14.71. * Vitiligo ☐ yes ☐ no
14.72. * Pallor (conjunctivae or palms of hands) ☐ yes ☐ no

14.73. * Deformities of the nails (flattening/koilonychia) ☐ yes ☐ no
14.74. * Pitting oedema (swelling of lower/upper limps or/and face) ☐ yes ☐ no
14.75. * Heart palpitations ☐ yes ☐ no
14.76. * Angina pectoris ☐ yes ☐ no
14.77. * Cardiac murmurs ☐ yes ☐ no
14.78. * Dyspnea ☐ yes ☐ no
14.79. * Pulmonary stethoscopic signs of abnormalities ☐ yes ☐ no
14.79.1. If yes, specify:____
14.80. * Nausea and/or vomiting ☐ yes ☐ no
14.81. * Hematemesis ☐ yes ☐ no
14.82. * Severe epigastric pain ☐ yes ☐ no
14.83. * Enlarged spleen ☐ yes ☐ no
14.84. * Melena ☐ yes ☐ no
14.85. * Other symptoms ☐ yes ☐ no
14.85.1. If yes, specify:____

SAMPLES COLLECTED

14.86. Malaria RDT ☐ negative ☐ PF ☐ PAN ☐ PF+PAN ☐ not done
14.87. HIV RDT, Determine ☐ negative ☐ positive ☐ not done
14.88. **Venous blood** draw ☐ done ☐ not done
14.88.1. if not done, why : ☐ refusal ☐ failure ☐ forgot
14.89. CPDA Tube : ☐ done ☐ not done ☐ not applicable
14.90. EDTA Tube (6ml + 2ml): ☐ done ☐ not done
14.91. Plain Tube (6ml + eppendorf): ☐ done ☐ not done
14.92. **Blood group:** ☐ done ☐ to be done
14.92.1. Result: ☐ A+ ☐ A- ☐ B+ ☐ B- ☐ AB+ ☐ AB- ☐ O+ ☐ O-
14.93. **Urine dipstick**
14.93.1. Albumin in the urine ☐ 0+ ☐ 1+ ☐ 2+ ☐ 3+ ☐ not done
14.93.2. Sugar in the urine ☐ 0+ ☐ 1+ ☐ 2+ ☐ 3+ ☐ 4+ ☐ 5+ ☐ not done
14.93.3. Leucocytes in the urine ☐ 0+ ☐ 1+ ☐ 2+ ☐ 3+ ☐ not done
14.93.4. Blood in urine ☐ 0+ ☐ 1+ ☐ 2+ ☐ 3+ ☐ not done
14.93.5. Ketones ☐ 0+ ☐ 1+ ☐ 2+ ☐ 3+ ☐ not done
14.93.6. Nitrite ☐ 0+ ☐ 1+ ☐ 2+ ☐ not done

CONCLUSION ON TODAY'S EXAMINATION

14.94. **Ailment/disease** diagnosed today ☐ yes ☐ no
14.94.1. If yes, specify (≥1 "x") ☐ Anaemia ☐ Malaria ☐ (Suspected) hypertension
☐ Urinary tract infection ☐ Syphilis ☐ HIV
☐ Upper respiratory tract infect. ☐ Diabetes
☐ Reproductive tract infection ☐ Other
14.94.1.1. If other, specify:____
14.95. **Treatment/Plan** prescribed today ☐ yes ☐ no
14.95.1. If yes, specify (≥1 "x") ☐ Coartem/ALU ☐ Quinine ☐ anti-helminth
☐ Iron ☐ Folic acid ☐ B12 ☐ Hemovit
☐ Antibiotics ☐ Anti-HT ☐ Painkillers ☐ Other
14.95.1.1. If painkillers or other, specify: ______________________________
14.95.2. Specify name, dosage and duration of treatment: ____________________
____
14.96. **Tetanus toxoid** immunization (TT) dose received today ☐ yes ☐ no
14.96.1. If no, state reason: ____
14.96.2. Nb of TT doses received until todays visit (excl. todays dose) _ _ ☐ Unknown
14.96.3. Last dose of TT received when _ _ / _ _ / _ _ _ _ ☐ Don't know
14.97. Additional notes:____
____

Next visit booked on: _ _ / _ _ / _ _ _ _, specify visit type: _____________

15. PATERNAL FORM (PREG)

15.1. Place of interview ☐ Korogwe District Hospital ☐ Home ☐ Other
15.1.1. If other, specify: ____________________
15.2. Name of study worker filling the form: ____________________
15.3. Date of filling CRF: (dd/mm/yyyy) _ _ / _ _ / _ _ _ _
15.4. **ID number and initials of mother:** FN _ _ _ _ _ _ _ _
15.5. Consent form signed by father ☐ yes ☐ no
15.6. Name of father (three names): ____________________

DEMOGRAPHIC DATA

15.7. Age _ _ years ☐ unknown
15.8. **Ethnic group** ☐ Sambaa ☐ Zigua ☐ Para ☐ Bondei ☐ Other ☐ unknown
15.8.1. if other, specify: ____________________
15.9. **Education** ☐ none ☐ primary school partially completed ☐ primary school finished ☐ ≥secondary school ☐ unknown
15.9.1. Do you know how to read and write ☐ yes ☐ no ☐ unknown
15.10. **Religion** ☐ Islamic ☐ Catholic ☐ Lutheran ☐ Angikana ☐ Hindi ☐Other
15.10.1. If other, specify:____________________
15.11. **Current occupation** ☐ Professional ☐ Business ☐ Service ☐ Farmer ☐ Other ☐ unknown
15.11.1. If other, specify:____________________
15.12. **No. of siblings** (genetic brothers/sisters, same father/mother) _ _ ☐ unknown
15.13. **Residence before age 15** for the majority of the time ☐urban ☐rural ☐urban/rural ☐ unknown
15.14. **Marital status** ☐ Married ☐ Divorced ☐ Separated ☐ Never married ☐ Widow ☐ Refuse answer

15.15. Living together with mother: ☐ yes ☐ no
If yes, go to question 15.17, if no answer question 15.16-15.16.7.2
15.16. **Residence:**
15.16.1. Village:____________________
15.16.2. Subvillage:____________________
15.16.3. Type of roof on the house: ☐ Bati ☐ Tin (madebe) ☐Thatch ☐ Mixed Thatch/Tin ☐ Other
15.16.3.1. if other, specify: ____________________
15.16.4. How many people usually sleep in your residence _ _
15.16.5. Who owns the house you live in ☐ self/spouse – built ☐ inherited ☐ rental ☐ others
15.16.5.1. if others, specify:____________________
15.16.6. Type of home toilet: ☐ flush ☐ pit latrine(choo cha shimo) ☐ no toilet
15.16.7. Source of water ☐ tap(bombani) ☐ well (kisima cha mdundiko) ☐ river/stream (mto) ☐ gutter water in tank (maji ya paa) ☐ pond/pool (bwawa) ☐ other
15.16.7.1. if other, specify: ____________________
15.16.7.2. if tap, well, or gutter water ☐ Private ☐ Public

Other chronic diseases

15.17. Diagnosed with a chronic illness by medical personnel (ask specifically about: diabetes type I or II (kisukari), kidney (figo), heart (moyo), thyroid (goiter) or lung (mapafu) disease, cancer (saratani), hypertension (shinikizo la damu), epilepsy (kifafa), lymphatic filariasis (matende), gastric ulcer (vidonda vya tumbo), autoimmune disorders (e.g. rheumatoid arthritis (rheumotism), chronic diarrhea (kuharisha kwa muda mrefu), chronic anaemia (upungufu ya damu kwa muda mrefu)
☐ yes ☐ no
15.17.1. if yes, specify____________________

15.18. **Any close relatives with a chronic disease** (genetic sister, brother, father, mother, grandparents, aunt, uncle) with (put >1 "x" if needed)
☐ Diabetes (kisukari) ☐ Cardiac disease
☐ Hypertension (shinikiza la damu) ☐ Severe undernutrition (utapiamlo mkali)
☐ Chronic anaemia(e.g.sickle cell, thalassemia)(upungufu wa damu kwa muda mrefu)
☐ Other ☐ No known disease

15.18.1. If yes or other, specify which relative and disease__________________________
____

15.19. **HIV status** according to the man:
☐ Unwilling to respond
☐ Positive
☐ Negative, will be re-testing today
☐ Negative, do not want re-testing
☐ Do not know, will be tested today
☐ Do not know, do not want testing

15.19.1. If positive, attending a CTC ☐ yes ☐ no

15.19.1.1. If yes, where:____

MEDICAL EXAMINATION *(all * should be filled by CO/AMO/MD)*

15.20. Blood pressure (BP) (mmHg)

15.20.1.	1st BP, left arm	_ _ _ / _ _ _
15.20.2.	1st BP, right arm	_ _ _ / _ _ _
15.20.3.	Difference in left and right arm (max 20 syst.; 10 diast.)	_ _ / _ _
15.20.4.	Reference arm (the arm with the highest BP)	☐ Right ☐ Left
15.20.5.	Reference arm (the arm with the highest BP), 2nd BP	_ _ _ / _ _ _
15.20.6.	Mean BP (1st and 2nd BP for reference arm)	_ _ _ / _ _ _
15.20.7.	Pulse (use last BP measurement)	_ _ _

SAMPLES COLLECTED

15.21. HIV RDT, Determine	☐ negative	☐ positive	☐ not done
15.21.1. Malaria RDT	☐ negative	☐ positive	☐ not done
15.22. **Venous blood** draw	☐ done	☐ not done	
15.22.1. if not done, why :	☐ refusal	☐ failure	☐ forgot
15.23. EDTA Tube (8ml, one tube):	☐ done	☐ not done	
15.24. **Blood sugar**	_ _ , _	☐ not done	
15.24.1. If measured, used	☐ fingerprick	☐ venous blood	
15.25. **Blood group:**	☐ to be done (lab) ☐ will not be done (lab)		
15.25.1. Result:	☐ A+ ☐ A- ☐ B+ ☐ B- ☐ AB+ ☐ AB- ☐ O+ ☐ O-		

CONCLUSION ON TODAY'S EXAMINATION

15.26. **Ailment/disease** diagnosed today ☐ yes ☐ no

15.26.1. If yes, specify (≥1 "x")
☐ Anaemia ☐ Malaria ☐ (Suspected) hypertension
☐ Urinary tract infection ☐ Syphilis ☐ HIV
☐ Upper respiratory tract infect. ☐ Diabetes
☐ Reproductive tract infection ☐ Other

15.26.1.1. If other, specify:____

15.27. **Treatment/Plan** prescribed today ☐ yes ☐ no

15.27.1. If yes, specify (≥1 "x")
☐ Coartem/ALU ☐ Quinine ☐ anti-helminth
☐ Iron ☐ Folic acid ☐ B12 ☐ Hemovit
☐ Antibiotics ☐ Anti-HT ☐ Painkillers ☐ Other

15.27.1.1. If painkillers or other, specify: ________________________________

15.27.2. Specify name, dosage and duration of treatment: ________________________
____

15.28. Additional notes:____
____
____

DATA ENTRY:

1st entry done by:______________________________Signature:______________ date:_ _/_ _/_ _ _ _

2nd entry done by:______________________________Signature:______________ date:_ _/_ _/_ _ _ _

16. SAMPLE from NEONATE (PREG)

16.1. Place ☐ Korogwe District Hospital ☐ Home ☐ Other
16.1.1. If other, specify:____
16.2. Date of investigation (dd/mm/yyyy) _ _ / _ _ / _ _ _ _
16.3. Time of investigation __ : __
16.4. Name of midwife/nurse/auxiliary worker: ________________________________
16.5. ID of mother: FN _ _ _ _ _ _ _ _

16.6. **1st newborn (the one delivered first)**

16.6.1.	Sex	☐ M	☐ F	☐ Unknown
16.6.2.	Weight (g) *(50g)*	1st_ _ _ _	2nd _ _ _ _	☐ Not done
16.6.3.	Length (infantometer) (cm) *(7mm)*	1st_ _ , _	2nd _ _ ,_	☐ Not done
16.6.4.	Head circumference (cm) *(5mm)*	1st_ _ , _	2nd _ _ ,_	☐ Not done
16.6.5.	Chest circumference (cm) *(5mm)*	1st_ _ , _	2nd _ _ ,_	☐ Not done
16.6.6.	Abdominal circumference (cm) *(5mm)*	1st_ _ , _	2nd _ _ ,_	☐ Not done
16.6.7.	Umbilical circumference (cm) *(5mm)*	1st_ _ , _	2nd _ _ ,_	☐ Not done
16.6.8.	Upper arm circumference (cm) *(2mm)*	1st_ _ , _	2nd _ _ ,_	☐ Not done
16.6.9.	Skinfold thickness triceps (mm) *(1mm)*	1st_ _ , _	2nd _ _ ,_	☐ Not done
16.6.10.	Skinfold thickness biceps (mm) *(1mm)*	1st_ _ , _	2nd _ _ ,_	☐ Not done
16.6.11.	Skinfold thickness subscapular (mm) *(1mm)*	1st_ _ , _	2nd _ _ ,_	☐ Not done
16.6.12.	Skinfold thickness thigh (mm) *(1mm)*	1st_ _ , _	2nd _ _ ,_	☐ Not done
16.6.13.	Length of foot (cm) *(2mm)*	1st_ _ , _	2nd _ _ ,_	☐ Not done
16.6.14.	Malformations/congenital disease	☐ yes	☐ no	☐ Unspecified

16.6.14.1. If yes, details : ____
16.6.15. Alive ☐ yes ☐ no
16.6.15.1. If no, state date of death _ _ / _ _ / _ _ _ _
16.6.15.2. If no, state cause of death: ____
____
____
____
16.6.16. If alive, any illness since deliver ☐ yes ☐ no
16.6.16.1. If yes, give details:____
____
____
____
16.6.16.2. If yes, state treatment including time period and doses;____________________
____
____
____
16.6.17. Venous sample collected today (4ml EDTA+1ml Eppendorf) ☐ yes ☐ no

16.7. **2nd newborn (if twins) (the one delivered last)**

16.7.1.	Sex	☐ M	☐ F	☐ Unknown
16.7.2.	Weight (g) *(50g)*	1st_ _ _ _	2nd _ _ _ _	☐ Not done
16.7.3.	Length (infantometer) (cm) *(7mm)*	1st_ _ , _	2nd _ _ ,_	☐ Not done
16.7.4.	Head circumference (cm) *(5mm)*	1st_ _ , _	2nd _ _ ,_	☐ Not done
16.7.5.	Chest circumference (cm) *(5mm)*	1st_ _ , _	2nd _ _ ,_	☐ Not done
16.7.6.	Abdominal circumference (cm) *(5mm)*	1st_ _ , _	2nd _ _ ,_	☐ Not done
16.7.7.	Umbilical circumference (cm) *(5mm)*	1st_ _ , _	2nd _ _ ,_	☐ Not done
16.7.8.	Upper arm circumference (cm) *(2mm)*	1st_ _ , _	2nd _ _ ,_	☐ Not done
16.7.9.	Skinfold thickness triceps (mm) *(1mm)*	1st_ _ , _	2nd _ _ ,_	☐ Not done
16.7.10.	Skinfold thickness biceps (mm) *(1mm)*	1st_ _ , _	2nd _ _ ,_	☐ Not done
16.7.11.	Skinfold thickness subscapular (mm) *(1mm)*	1st_ _ , _	2nd _ _ ,_	☐ Not done
16.7.12.	Skinfold thickness thigh (mm) *(1mm)*	1st_ _ , _	2nd _ _ ,_	☐ Not done
16.7.13.	Length of foot (cm) *(2mm)*	1st_ _ , _	2nd _ _ ,_	☐ Not done
16.7.14.	Malformations/ congenital disease	☐ yes	☐ no	☐ Unspecified

16.7.14.1. If yes, details : ____

16.7.15. Alive ☐ yes ☐ no

16.7.15.1. If no, state date of death _ _ / _ _ / _ _ _ _

16.7.15.2. If no, state cause of death: ____

____

____

____

16.7.16. If alive, Any illness since deliver ☐ yes ☐ no

16.7.16.1. If yes, give details:____

____

____

____

16.7.16.2. If yes, state treatment including time period and doses;________________________

____

____

____

16.7.17. Venous sample collected today (4ml EDTA+1ml Eppendorf) ☐ yes ☐ no

DATA ENTRY:

1st entry done by:____Signature:______________ date:_ _/_ _/_ _ _ _

2nd entry done by:____Signature:______________ date:_ _/_ _/_ _ _ _
